# Iron Catalyzed Aryl–Aryl Kumada Cross‐Coupling: A Mechanistic and Computational Investigation

**DOI:** 10.1002/anie.3094782

**Published:** 2026-03-13

**Authors:** Jatin Panda, Magali Gimeno, Amrita Gogoi, Zeqing Chen, Subhash Garhwal, Laura Levy, Alexander Kaushansky, Natalia Fridman, Jos Briggs‐Pritchard, Renana Gershoni‐Poranne, Michael L. Neidig, Graham de Ruiter

**Affiliations:** ^1^ Department: Schulich Faculty of Chemistry Technion – Israel Institute of Technology, Technion City Haifa Israel; ^2^ Inorganic Chemistry Laboratory University of Oxford Oxford UK; ^3^ the Resnick Sustainability Center for Catalysis Technion – Israel Institute of Technology, Technion City Haifa Israel

**Keywords:** computational chemistry, iron, Kumada cross‐coupling, mechanism, Mössbauer spectroscopy

## Abstract

The widespread use of precious metal catalysts in C–C bond‐forming reactions is increasingly challenged by concerns over toxicity, cost, and limited availability. As a sustainable alternative, iron offers distinct advantages in cross‐coupling chemistry, but its broader application has been hindered by limited mechanistic understanding. Here, we report a mechanistically driven investigation of aryl–aryl Kumada cross‐coupling catalyzed by our previously reported iron complex [(PC_NHC_P)FeCl_2_] (**2**). Through a combination of multinuclear NMR, ^57^Fe Mössbauer spectroscopy, single‐crystal X‐ray diffraction, and reactivity studies, we identify and characterize key in situ formed intermediates, including mono‐ and bis‐arylated iron species, along the catalytic pathway. While PCP‐ligated Fe(II) complexes support two‐electron chemistry, our findings uncover a distinct radical mechanism responsible for the efficient formation of the biaryl products. Furthermore, we demonstrate that small coordinating molecules, such as N_2_, significantly influence the speciation and reactivity of the iron catalyst. These insights advance fundamental understanding of iron‐mediated cross‐coupling and provide new design principles for sustainable C(sp^2^)–C(sp^2^) bond construction.

## Graphical abstract

Despite intense interest, the mechanism of iron‐catalyzed aryl–aryl cross‐coupling remains poorly understood. Combining Mössbauer spectroscopy, kinetics analysis, and DFT computations these studies reveal a novel Fe(I)/Fe(II)/Fe(III) catalytic for aryl–aryl cross‐coupling mediated by an Fe(II) PC_NHC_P Pincer complex. These findings close a key knowledge gap and offer design principles for sustainable iron‐mediated cross‐coupling.

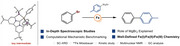

## 1 Introduction

**SCHEME 1:**
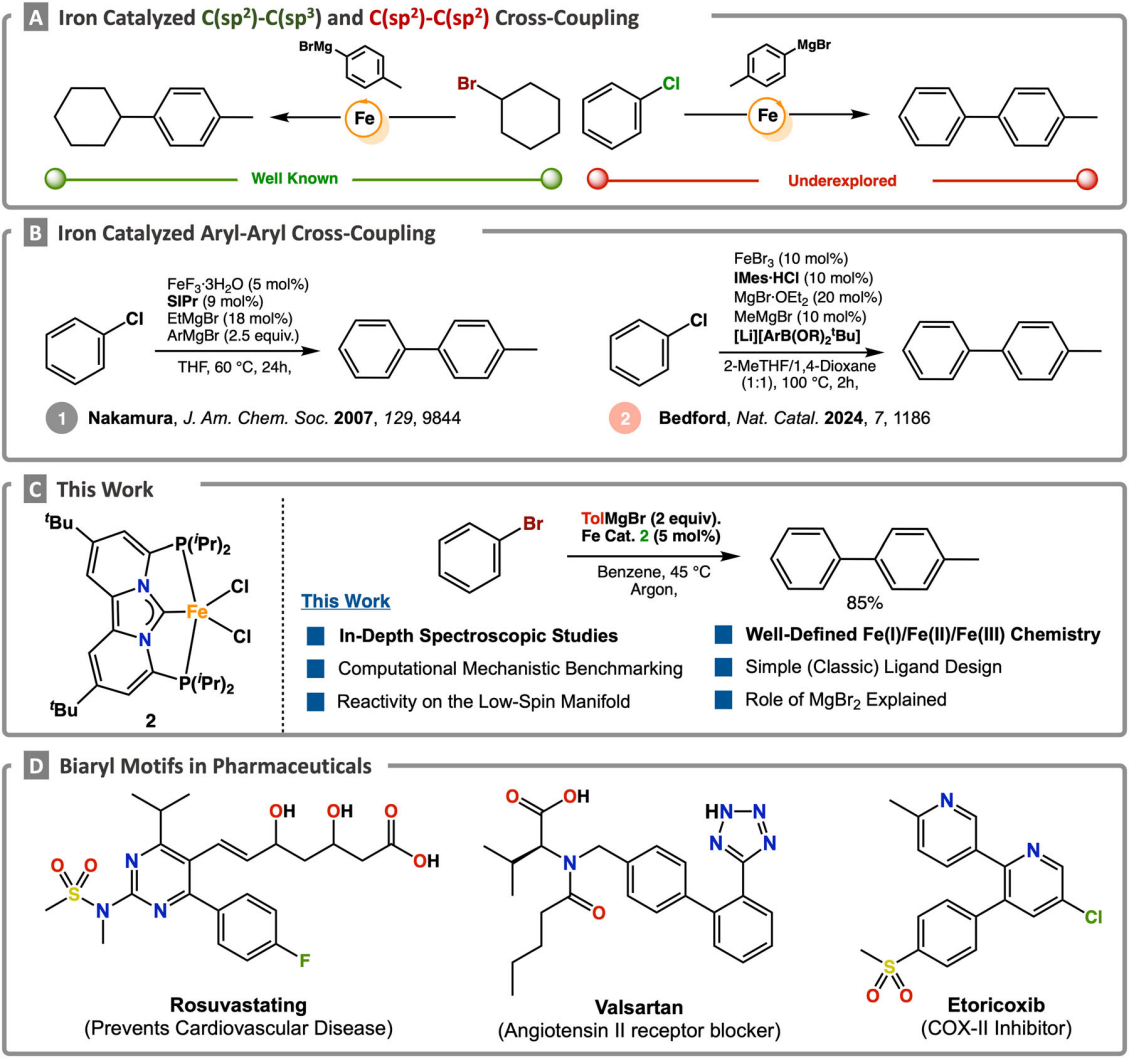
(A) General examples of iron‐catalyzed carbon─carbon bond forming reactions. (B) selected examples of iron‐catalyzed aryl–aryl cross‐coupling reactions. (C) Herein reported mechanistic approach for the aryl–aryl Kumada cross‐coupling reaction catalyzed by a PC_NHC_P iron pincer complex. (D) Selected examples of bisaryl motifs in pharmaceuticals.

Transition‐metal‐catalyzed cross‐coupling reactions represent some of the most powerful and widely applied transformations in modern organic chemistry [1, 2, 3, 4, 5]. Typically mediated by transition metals such as palladium and nickel, these reactions enable the formation of carbon–carbon and carbon–heteroatom bonds from a broad range of readily available precursors [6, 7, 8, 9, 10, 11, 12, 13, 14, 15]. Their profound impact on the chemical sciences is reflected in their widespread use in the synthesis of complex natural products, pharmaceuticals, and agrochemicals [16, 17, 18, 19, 20]. Among these applications, the construction of biaryl motifs holds particular significance [21, 22, 23, 24, 25], as evidenced by their prevalence in numerous therapeutic agents, including Lipitor, Crestor, Nucoxia, and Celebrex (Scheme 1) [20, 26, 27, 28, 29, 30]. While palladium has become nearly synonymous with cross‐coupling catalysis, its high cost and limited natural abundance, as well as related ecological concerns, have prompted a growing interest in more sustainable alternatives [31, 32, 33]. In this context, first‐row, earth‐abundant transition metals, such as iron, offer attractive alternatives, not only due to their low toxicity and ready availability, but also because of their distinctive and potentially complementary reactivity profiles (Scheme 1A) [34, 35, 36, 37, 38, 39, 40].

Indeed, early studies established that simple iron halide salts could promote both the homo‐ and cross‐coupling of aryl nucleophiles with electronically activated aryl electrophiles [41, 42, 43]. However, the first efficient iron‐catalyzed aryl–aryl Kumada cross‐coupling was not realized until the seminal work of Nakamura and co‐workers [44]. Building on this breakthrough, subsequent studies demonstrated that alternative iron salts and a broader range of aryl electrophiles, including aryl tosylates and sulfamates, could be employed under similar conditions [45, 46, 47, 48]. More recently, Bedford and co‐workers reported the first iron‐catalyzed aryl–aryl Suzuki–Miyaura‐type cross‐coupling, also enabled by a privileged NHC ligand, marking a significant expansion in the scope of iron‐mediated biaryl formation. This transformation is proposed to proceed via a non‐classical Fe(I)/Fe(III) redox manifold, highlighting the expanding mechanistic diversity accessible through iron catalysis [49, 50].

Despite these notable advances, iron‐catalyzed cross‐coupling has yet to achieve the maturity and reliability of its precious metal counterparts. Current limitations—including narrow substrate scope, modest product yields, and the frequent requirement for excess ligand, nucleophile, or additives—must be addressed for these transformations to become competitive in practical synthesis. Overcoming these challenges to enable rational method design will require both the development of tailored ligand frameworks and a deeper mechanistic understanding. For example, while diphosphine ligands have been widely employed in iron‐catalyzed C(sp^2^)–C(sp^3^) alkyl–aryl couplings [51, 52, 53, 54, 55, 56, 57, 58, 59, 60, 61, 62], their application in C(sp^2^)–C(sp^2^) biaryl coupling remains largely unexplored. Likewise, although influential contributions by Nakamura, Duong, and Bedford have advanced mechanistic understanding primarily through computational studies, direct experimental insight into the nature of the active iron species in biaryl coupling remains scarce, particularly in contrast to alkyl–aryl cross‐coupling reactions, which have been extensively investigated.

Although evidence for both radical and two‐electron pathways in iron‐catalyzed biaryl coupling reaction has been presented, key questions regarding iron speciation, oxidation states, and the electronic structure of active intermediates remain unresolved. As a result, intermediates spanning Fe(I), Fe(II), and Fe(III) oxidation states have all been proposed, underscoring the mechanistic complexity and the need for direct spectroscopic interrogation of the in situ formed species to enable mechanistically guided, rational method development.

## 2 Results and Discussion

**SCHEME 2:**
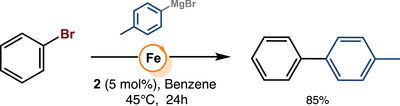
Cross‐coupling of bromobenzene with *p*‐tolylmagnesium bromide catalyzed by complex **2**.

Inspired by the work of Chirik and co‐workers, who demonstrated that Fe(0) CNC pincer complexes undergo facile oxidative addition of aryl halides [63], we hypothesized that our PC_NHC_P‐ligated Fe(0) complex, [(PC_NHC_P)Fe(N_2_)_2_] (**1**) [64, 65], might facilitate aryl–aryl cross‐coupling, provided the reaction is initiated via oxidative addition of the aryl electrophile. Surprisingly, under an atmosphere of dinitrogen, neither complex **1** nor its Fe(II) analog, [(PC_NHC_P)FeCl_2_] (**2**), exhibited any catalytic activity under standard cross‐coupling conditions (see Supporting Information for experimental details). However, when the reaction was performed under argon with 5 mol% of either complex **1** or **2**, in the presence of *p*‐tolylmagnesium bromide (40 equiv.) and bromobenzene (20 equiv.), the desired biaryl product was obtained in 85% yield (Scheme 2).

This pronounced difference in the reactivity of complexes **1** and **2**, both in comparison to Chirik's CNC–iron system and to the monodentate NHC‐based iron catalysts reported by Nakamura, Duong, and Bedford, motivated a comprehensive spectroscopic and computational investigation into the underlying reaction mechanism *(vide infra*). Specifically, we aimed to elucidate: (i) the speciation of iron under catalytic conditions; (ii) the spin states of the relevant iron intermediates; (iii) the influence of the reaction atmosphere (N_2_ vs. Ar) on catalytic performance; and (iv) the operative electronic pathway, namely whether the transformation proceeds via consecutive one‐electron (radical) or two‐electron redox processes.

### 2.1 Independent Synthesis of Potential Intermediates

**SCHEME 3:**
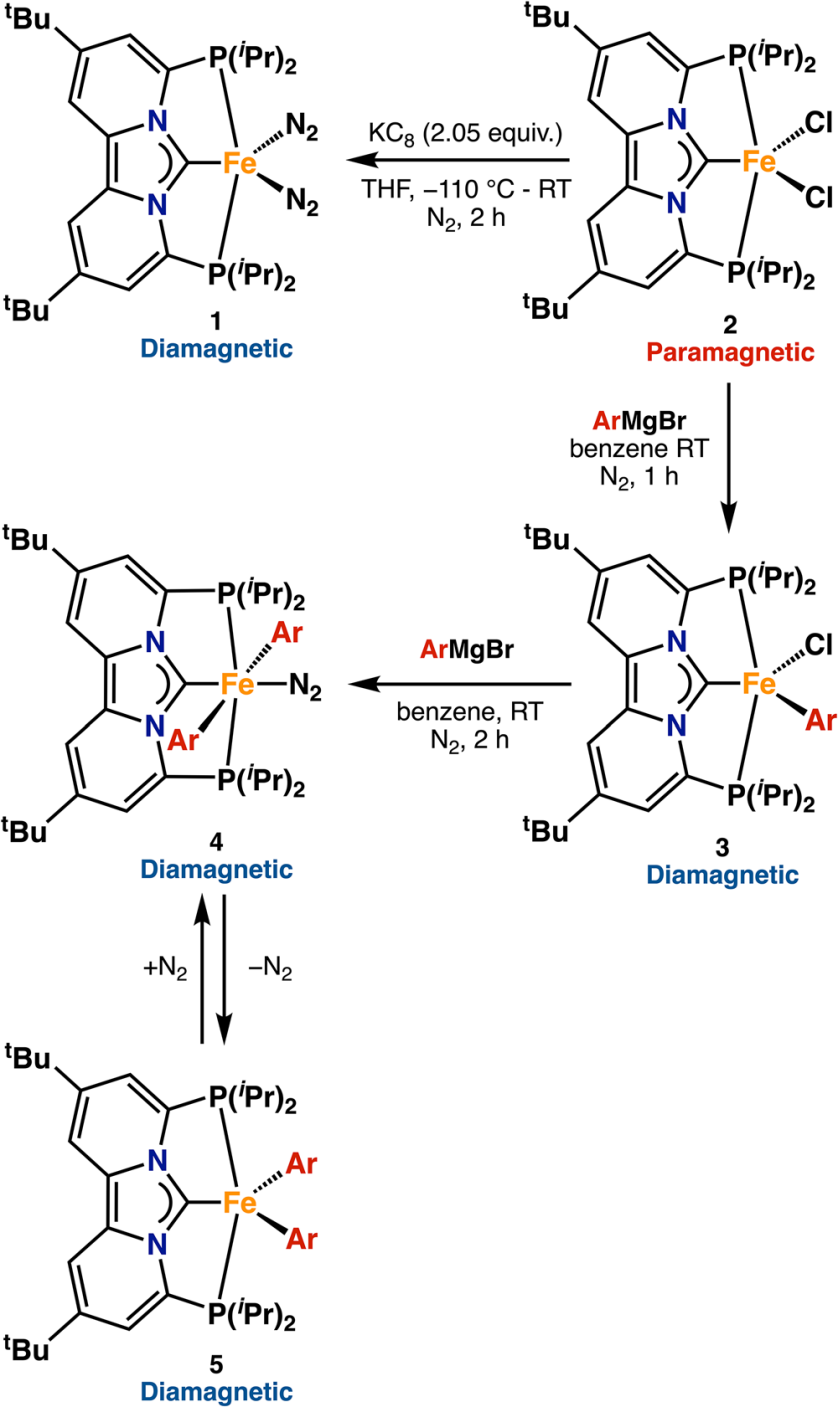
Independent synthesis of complexes **1–5**.

**FIGURE 1:**
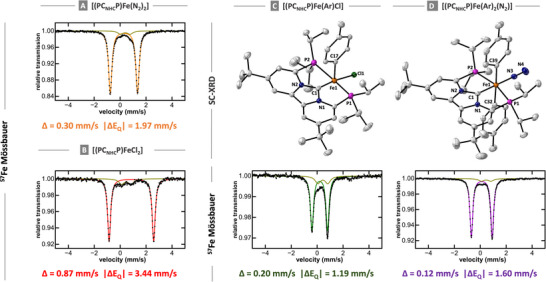
Solid‐state structures and solid 80 K ^57^Fe Mössbauer spectra of (A) [(PC_NHC_P)Fe(N_2_)_2_] (**1**), (B) [(PC_NHC_P)FeCl_2_] (**2**), (C) [(PC_NHC_P)Fe(Ar)Cl] (**3**), and (D) [(PC_NHC_P)Fe(Ar)_2_(N_2_)] (**4**). Co‐crystallized solvents and hydrogen atoms are not shown for clarity. Ellipsoids are plotted at the 30% probability level. ^57^Fe Mössbauer parameters for the impurity (<5%; dark yellow) are *δ* = 0.44 mm/s |Δ*E*_Q_| = 0.74 mm/s.

To better understand iron speciation under catalytic conditions and to benchmark spectroscopic signatures against well‐defined reference compounds, we first sought to independently synthesize a series of Fe(II) PC_NHC_P pincer complexes that are likely to form during catalysis (Scheme 3). To initiate these studies, complexes **1** and **2** were prepared according to previously reported procedures [64, 66]. The solid‐state ^5^ ^7^Fe Mössbauer spectrum of complex **1** (Figure 1A, orange trace) displays a doublet with an isomer shift (*δ*) of 0.30 mm/s and a quadrupole splitting (|Δ*E*_Q_|) of 1.97 mm/s (Table 1). A minor impurity (<5%) is also observed (dark yellow trace), exhibiting *δ* = 0.44 mm/s and |Δ*E*_Q_| = 0.74 mm/s, which is an impurity present in the synthesis of complex **2** that is carried through the synthesis of all the other complexes. As such, the impurity is observable in all Mössbauer spectra throughout this work. Notwithstanding, the Mössbauer parameters of **1** are consistent with a low‐spin Fe(0) complex and are similar to the values reported for other structurally related Fe(0)–N_2_ complexes [63]. Similarly, the solid‐state Mössbauer spectrum of complex **2** exhibits parameters of *δ* = 0.87 mm/s and |Δ*E*_Q_| = 3.44 mm/s (Table 1), consistent with a high‐spin Fe(II) dihalide species [53, 55]. Although the analogous bromide complex, [(PC_NHC_P)FeBr_2_], displays nearly identical Mössbauer parameters (Table 1), its synthesis typically resulted in the formation of large amounts (>10%) of impurities, rendering it unsuitable as a starting material for further mechanistic studies (Figure S16). As a result, subsequent experiments under catalytically relevant conditions were conducted using ^5^ ^7^FeCl_2_ as starting material, which reproducibly afforded clean formation of complex **2**, as confirmed by freeze‐trapped ^5^ ^7^Fe Mössbauer spectroscopy (Figure S15).

**TABLE 1:** Key ^57^Fe Mössbauer parameters for PC_NHC_P iron complexes **1–5** reported in this study.

Category	Complex	*δ* (mm/s)	|Δ*E*_Q_| (mm/s)
**Dihalide**	[(PC_NHC_P)FeCl_2_] (**2**)	0.87	3.44
	[(PC_NHC_P)FeBr_2_]	0.87	2.17
**Mono‐aryl**	[(PC_NHC_P)Fe(Ar)Cl] (**3**)	0.20	1.19
**Bis‐aryl**	[(PC_NHC_P)Fe(Ar)_2_(N_2_)] (**4**)	0.12	1.60
	[(PC_NHC_P)Fe(Ar)_2_] (**5a**)	0.07	1.80
	[(PC_NHC_P)Fe(Ar)_2_] (**5b**)	0.12	2.31
**Iron(0)**	[(PC_NHC_P)Fe(N_2_)_2_] (**1**)	0.30	1.97

Having established reliable synthetic protocols for complex **1** and ^5^ ^7^Fe‐labeled complex **2**, we next investigated whether the corresponding iron mono‐ and bis(aryl) complexes **3** and **4** could be accessed. Indeed, addition of *p*‐tolylmagnesium bromide (1.0 equiv.) to a solution of complex **2** in benzene resulted in an immediate and marked color change from red/brown to dark green. Analysis of the resulting reaction mixture by ^1^H‐NMR and ^31^P‐NMR spectroscopy revealed the clean formation of a new diamagnetic species (Figures S1–S5), which was unambiguously identified as [(PC_NHC_P)Fe(Ar)Cl] (**3**) by single‐crystal X‐ray diffraction (SC‐XRD). The solid‐state structure of **3** is shown in Figure 1C and features an iron metal center in a distorted square pyramidal geometry (*τ* ^5^ = 0.147), with the aryl group in the axial position. The Fe–C_1_ bond distance of 1.842(7) Å is consistent with a low‐spin Fe(II) formulation and comparable to values reported for related aryl–Fe(II) complexes. Interestingly, the change from high‐spin Fe(II) to low‐spin iron Fe(II) not only results in a reduction of the Fe–C_1_ bond distance from 2.064(6) Å in **2** to 1.842(7) Å in **3**, but also results in a significant reduction of the average iron–phosphine (Fe–P) bond distance from 2.774(2) Å in **2** to 2.263(16) Å in **3** (Table S3). This contraction results in a pronounced decrease in the P–C_1_–P bite angle (126.58° in **2** vs. 112.89° in **3**), a structural change likely to impact the rate of transmetalation during catalysis. The solid‐state ^5^ ^7^Fe Mössbauer spectrum of **3** (Figure 1C) features a single quadrupole doublet with *δ* = 0.20 mm/s and |Δ*E*_Q_| = 1.19 mm/s (Table 1), consistent with a low‐spin Fe(II) center [67, 68].

These Mössbauer parameters closely resemble those reported by Chirik and co‐workers for analogous octahedral mono‐aryl Fe(II) complexes supported by strong‐field CNC pincer ligands [63]. A notable distinction, however, is that dinitrogen remains bound throughout the transmetalation event in Chirik's system, while in our case, dinitrogen is only bound at the end of the transmetalation event (*vide infra*).

Encouraged by the successful isolation of **3**, we next explored the synthesis of the diaryl complex **4** by adding two equivalents of *p*‐tolylmagnesium bromide to a solution of **2** in benzene. Akin to complex **3**, complex **4** displays diamagnetic ^1^H and ^3^ ^1^P NMR spectra, including a distinct phosphine resonance at *δ* = 95.2 ppm (Figures S6–S10). Crystals suitable for SC‐XRD were obtained by slow evaporation of a concentrated solution of **4** in benzene. The solid‐state structure of **4** is shown in Figure 1D and features an iron center in an octahedral geometry, with two aryl ligands in the axial positions and a single N_2_ ligand in the equatorial plane. In complex **4**, the iron‐aryl (Fe–C_ar_) distances of 2.075(3) and 2.069(3) Å are significantly elongated compared to monoaryl complex **3** (1.842(7) Å), likely due to the strong *trans*‐influence exerted by the mutually trans aryl ligands. By contrast, the Fe–C_1_ and Fe–P bond lengths remain largely unchanged between **3** and **4** (Table S3), supporting a consistent low‐spin Fe(II) environment for both species. The low‐spin configuration of complex **4** was further confirmed by solid‐state ^5^ ^7^Fe Mössbauer spectroscopy, which revealed a single doublet with *δ* = 0.12 mm/s and |Δ*E*_Q_| = 1.60 mm/s (Table 1).

It is noteworthy that both compounds exhibit considerable thermal stability. Prolonged heating of either complex at 80 °C resulted in no observable decomposition, nor was any biaryl product detected, which suggests no reductive elimination occurs from complex **4**. This lack of reactivity is consistent with the expected difficulty of C–C bond formation from an electron‐rich Fe(II) center, in contrast to the more favorable reductive elimination from Fe(III) species. Additionally, the presence of the equatorial N_2_ ligand in **4** likely inhibits the approach and coupling of the mutually trans aryl ligands, offering a mechanistic rationale for the absence of cross‐coupling under a dinitrogen atmosphere.

To gain deeper mechanistic insight into the reactivity of the iron intermediates under catalytically relevant conditions, we performed a series of stoichiometric experiments under argon atmosphere (*vide infra*). These studies were conducted in benzene‐d_6_ at 45 °C using a catalyst concentration of 42 mM (catalytic conditions). For clarity, the mechanistic discussion is organized into two parts: (i) investigation of the transmetalation step, and (ii) evaluation of the reactivity of iron complexes **1–4** toward the electrophilic aryl halide. Finally, freeze‐trapped ^57^Fe Mössbauer spectroscopy was used to investigate the iron speciation during catalysis, after which a mechanistic proposal will be postulated and computationally evaluated.

### 2.2 Transmetalation From the Iron‐Dichloride

**FIGURE 2:**
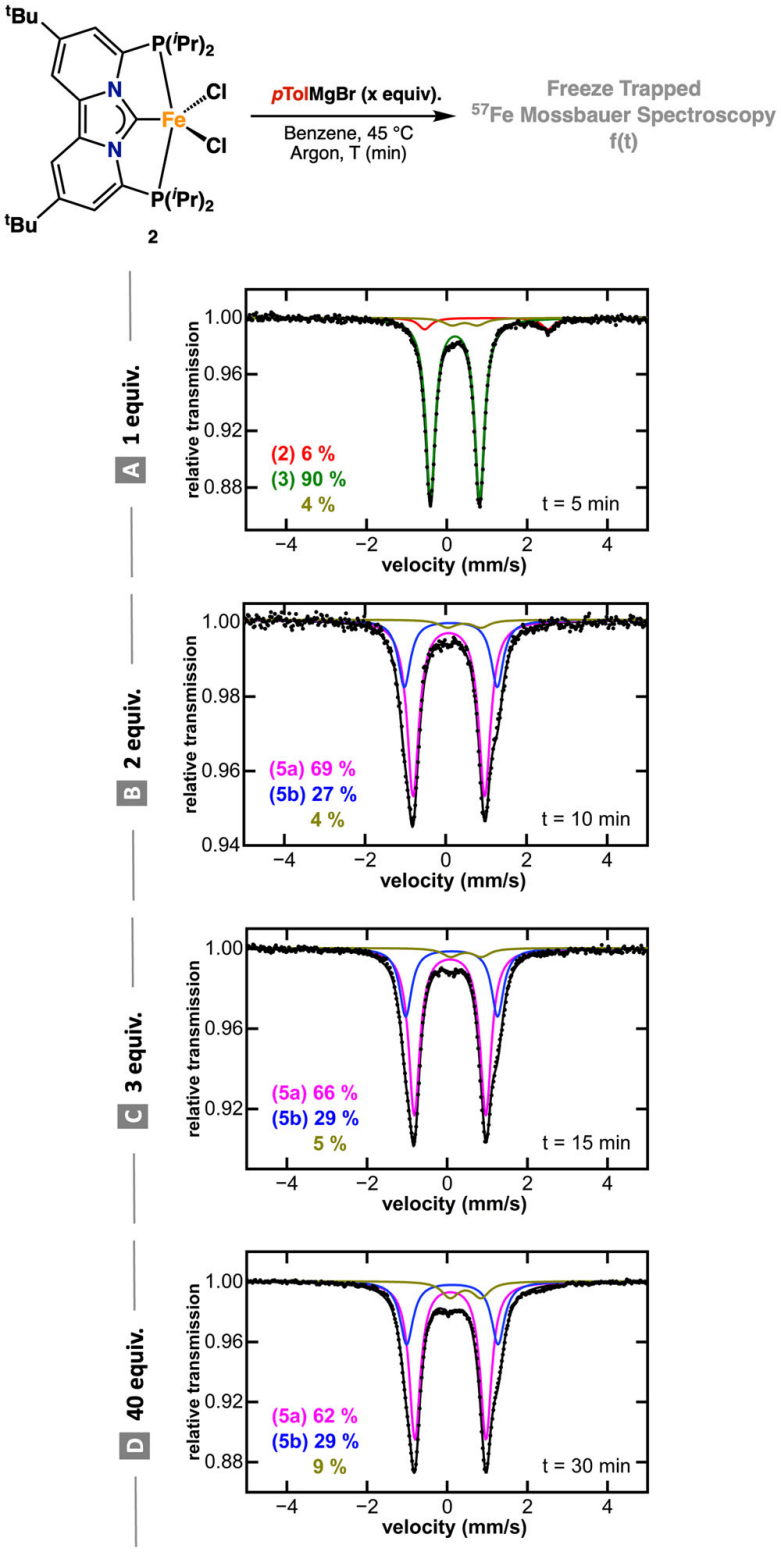
Freeze‐trapped 80 K ^57^Fe Mössbauer spectra of reaction of **2** with different equivalents of *p*‐tolylmagnesium bromide. Each spectrum was obtained from independent solutions of **2**.

With the spectroscopic properties of complexes **1–4** established, we next sought to investigate the iron speciation during transmetalation with different equivalents of *p*‐tolylmagnesium bromide under catalytically relevant conditions. Addition of one equiv. of *p*‐tolylmagnesium bromide to a solution of **2** in benzene at 45 °C under argon led to clean formation of complex **3** (>90%) as indicated by the freeze‐trapped 80K ^57^Fe Mössbauer spectroscopy (Figure 2A). The observed Mössbauer parameters of *δ* = 0.21 mm/s and a quadrupole splitting of |Δ*E*_Q_| = 1.23 mm/s are consistent with those of **3** in the solid state (Table 1 and Figure 1C). Likewise, the ^1^H and ^31^P NMR spectra of the resulting reaction mixture clearly indicate the formation of complex **3** within a few minutes (Figures S36 and S37). These experiments also suggest that under the experimental reaction conditions, the first transmetalation is nearly instantaneous.

By contrast, addition of two equivalents of *p*‐tolylmagnesium bromide to a solution of **2** in benzene at 45°C under argon did not yield **4** under the experimental conditions. Instead, two new species, **5a** and **5b**, were observed (Figure 2B), whose ratio remained unchanged even upon addition of excess Grignard reagent (Figure 2C,D). No additional iron‐containing species were detected by freeze‐trapped ^5^ ^7^Fe Mössbauer spectroscopy, indicating that **5a** and **5b** are stable in the presence of excess nucleophile and that higher‐transmetallates or “ate” complexes (e.g., [Fe(Ar)_3_]^−^) are not formed under these conditions (Figures S43 and S44). Notwithstanding, the ^57^Fe Mössbauer parameters of **5a** (*δ* = 0.07 mm/s and |Δ*E*_Q_| = 1.80; Figure 2B, magenta trace) and **5b** (*δ* = 0.12 mm/s and |Δ*E*_Q_| = 2.31 mm/s; Figure 2B, blue trace), are similar to those of complex **4**, warranting their tentative assignment as low‐spin Fe(II) bis‐arylated species, consistent with our computational data (Tables S12 and S13). The comparable isomer shifts indicate similar electronic environments of the iron metal center in **5a** and **5b**, while their different quadrupole splitting suggests distinct geometric arrangements at iron. Interestingly, room‐temperature ^3^ ^1^P NMR spectroscopy revealed a single phosphine resonance (*δ* = 83.7 ppm), consistent with rapid interconversion of **5a** and **5b** on the NMR timescale (Figure S40). While this behavior primarily supports dynamic exchange between closely related species, it may also reflect an equilibrium between monomeric and dimeric species in solution (Figures S14 and S20). To probe this equilibrium further, we exposed a solution containing a mixture of **5a** and **5b** to one atmosphere of dinitrogen, which resulted in the clean formation of complex **4**, as confirmed by ^1^H, ^3^ ^1^P NMR, and ^5^ ^7^Fe Mössbauer spectroscopy (Figures S45–S47). Conversely, subjecting a benzene solution of complex **4** to repeated freeze–pump–thaw cycles regenerated **5a** and **5b**, demonstrating that, in the absence of N_2_, **5a** and **5b** are bis‐arylated species. Furthermore, our ^1^H and ^31^P NMR data are consistent with low‐spin Fe(II) formulations with C_2_v symmetry suggesting either the presence of geometric isomers (**5a**/**5b**) or a monomer/dimer equilibrium (Figures S11‐S13, and S18–S24). Solvation effects, for example, coordination of residual THF to generate octahedra metal complexes, were excluded, as addition of two equiv. of *p*‐tolylmagnesium bromide to a solution of complex **2** in THF only resulted in the formation of complex **3**, as judged by ^57^Fe Mössbauer spectroscopy (Figure S41).

**FIGURE 3:**
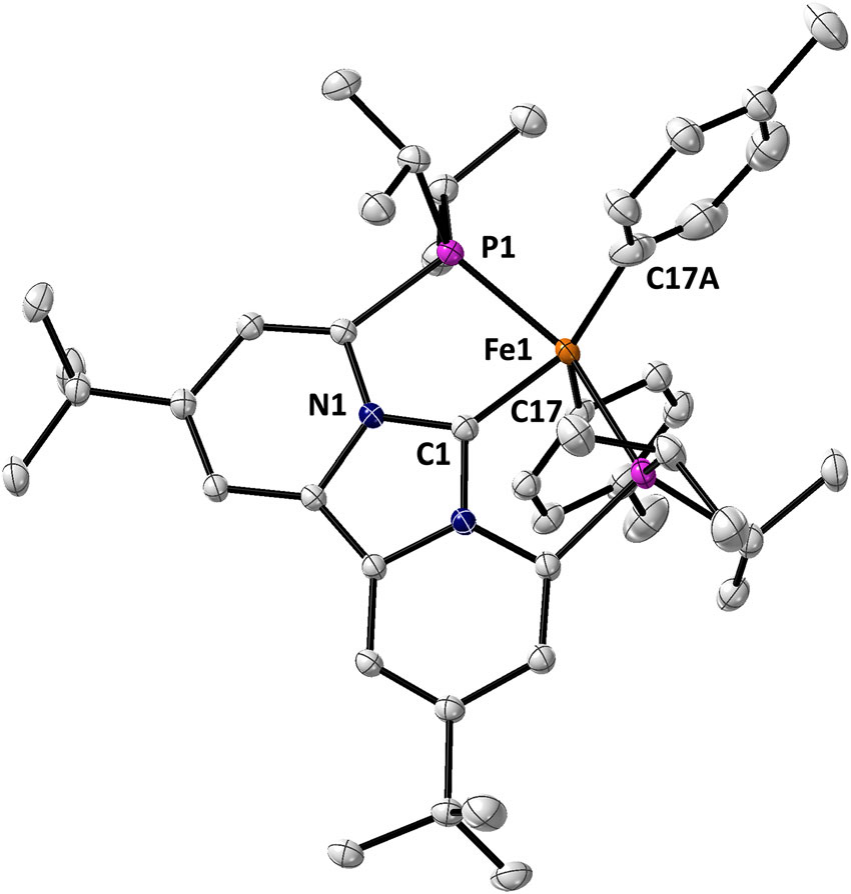
Solid‐state structures of [(PC_NHC_P)Fe(Ar)_2_] (**5**). Co‐crystallized solvents and hydrogen atoms are not shown for clarity. Ellipsoids are plotted at the 30% probability level.

Structural insight into these elusive intermediates was obtained through the independent synthesis of compound **5** (Figure 3), identified by SC‐XRD as a bis‐arylated Fe(II) complex adopting a distorted trigonal bipyramidal (TBP) geometry (Figure 3; *τ*_5_ = 0.673), where the Fe–C_aryl_ and Fe–P bond distances in **5** closely match those of **4** (Table S3). These data support our current assignment of **5a** and **5b** as geometric isomers, likely TBP and square pyramidal (SP), of the same five‐coordinate, low‐spin Fe(II) complex. While SC‐XRD confirms a TBP geometry for **5**, this structure may not directly correspond to the major solution species observed by Mössbauer spectroscopy. Accordingly, definitive assignment of the Mössbauer signals to specific geometries is not yet possible. Overall, these data show that transmetalation of Fe(II) complex **2** with aryl Grignard reagents yields well‐defined low‐spin Fe(II) mono‐ and bis‐arylated intermediates. Reversible interconversion between **4** and **5a/5b** highlights the key role of dinitrogen in stabilizing the bis‐arylated species. These results emphasize the sensitivity of iron speciation under different atmospheres, which greatly influences the cross‐coupling reaction.

### 2.3 Reaction of Complexes 2–5 With Electrophile

**FIGURE 4:**
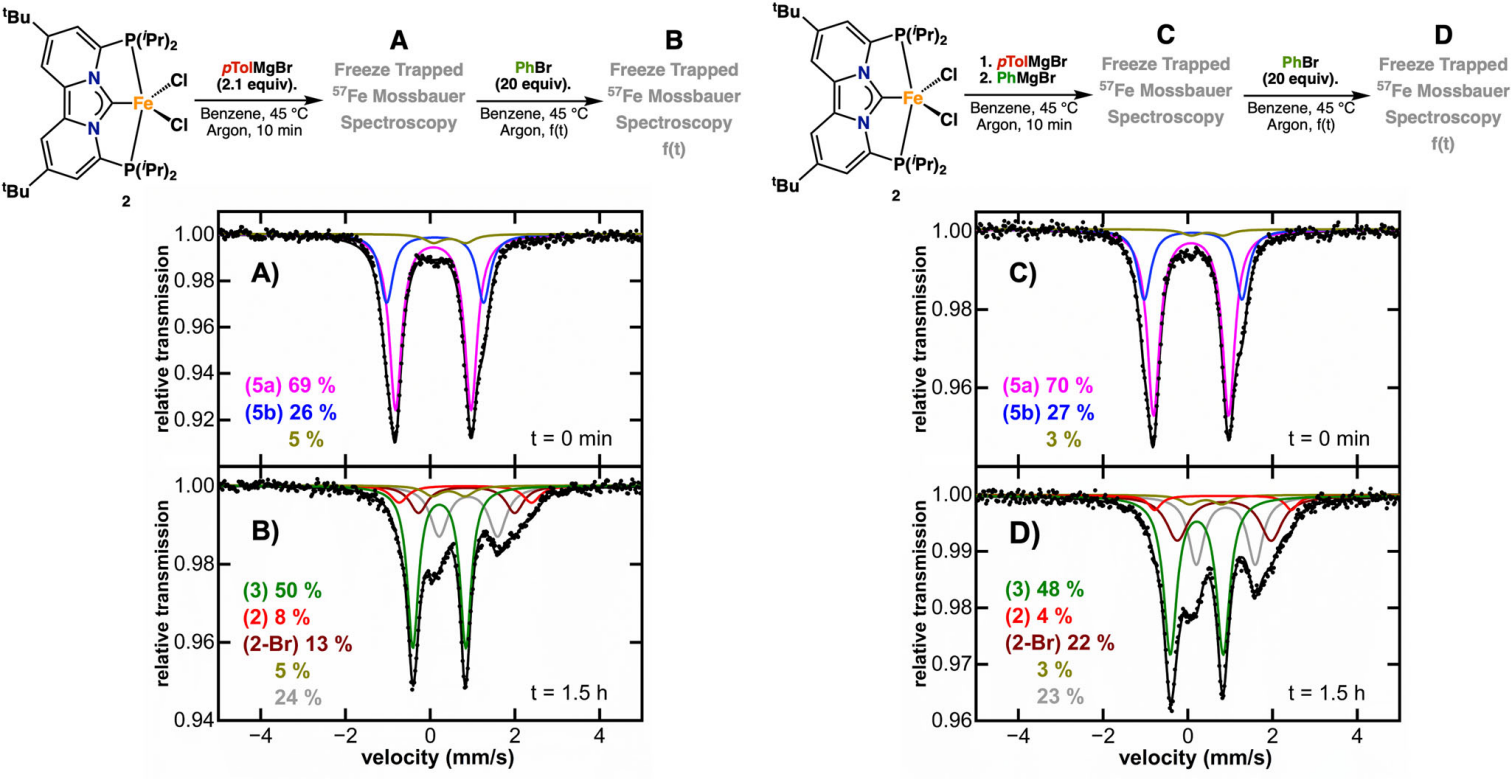
(Left) Freeze‐trapped 80 K ^57^Fe Mössbauer spectra of the in situ formed homo bis‐aryl species **5** before (A) and after addition (B) addition of 20 equiv. of bromobenzene. (Right) Freeze‐trapped 80 K ^57^Fe Mössbauer spectra of the in situ formed hetero bis‐aryl species 5 before (C) and after addition (D) addition of 20 equiv. of bromobenzene.

Having established that bis‐arylated Fe(II) species dominate under transmetalation conditions with excess *p*‐tolylmagnesium bromide, we next examined the reactivity of iron complexes **2–5** in the presence of excess bromobenzene (20 equiv.). Under catalytically relevant conditions, neither Fe(II) precursor complex **2**, mono‐aryl complex **3**, nor bis‐aryl complex **4** showed any reactivity toward bromobenzene, as confirmed by GC‐MS analysis, positioning bis‐aryl species **5** as the key reactive intermediate. To accurately mimic the catalytic experiments (*vide infra*), complex **5** (42 mM), as a mixture of **5a** and **5b**, was synthesized in situ (Figure 4A) and subsequently treated with bromobenzene. Freeze‐trapped ^5^ ^7^Fe Mössbauer spectroscopy revealed complete consumption of **5** within 90 min, accompanied by the formation of **3** (∼50%) and a mixture of FeX_2_ species (**2**, X═Br or Cl), which remained unchanged after 24 h (Figure 4B). Interestingly, before the addition of the electrophile, no evidence of reductive elimination of a bis‐aryl species was detected, indicating that Fe‐mediated C–C bond formation does not occur directly from **5** under these conditions (Figure 4A). However, after addition of the electrophile, analysis of the organic products by GC/MS revealed the formation of ~45% homo‐coupled nucleophile (4,4’‐dimethylbiphenyl) and ~30% homo‐coupled electrophile (biphenyl). The absence of cross‐coupled product is significant as it indicates that reductive elimination occurs directly from the iron metal center, but only after oxidation of the Fe(II) bis‐aryl species **5**. More specifically, reaction of **5** with bromobenzene leads to a one‐electron oxidation to form the Fe(III) complex [(PC_NHC_P)Fe(Ar)_2_Br] (**7**), which subsequently undergoes rapid reductive elimination to yield the homo‐coupled biaryl and the Fe(I) halide species [(PC_NHC_P)FeBr] (**8**). The formed aryl radical can either recombine with **8** to produce the observed mono‐aryl species **3**, or recombine with another radical to produce biphenyl as observed by GC/MS. To directly confirm that cross‐coupling can proceed from a defined Fe(II) bis‐aryl intermediate, we repeated the experiment using a heteroleptic bis‐arylated species formed in situ by sequential addition of PhMgBr and *p*TolMgBr to complex **2**. Treatment of this heteroleptic bis‐aryl species with excess bromobenzene resulted in nearly identical Mössbauer spectra after 90 min (Figure 4D). However, GC/MS analysis now revealed 44% cross‐coupled product (4‐methylbiphenyl), along with 6% 4,4’‐dimethylbiphenyl and ∼50% biphenyl, providing strong evidence that cross‐coupling is initiated by homolytic bond cleavage of the aryl electrophile, which induces reductive elimination from the resulting Fe(III) center. Moreover, these data highlight one of the rare cases in which C(sp^2^)–C(sp^2^) bond formation occurs directly at an iron center through reductive elimination rather than through bond homolysis.

**SCHEME 4:**
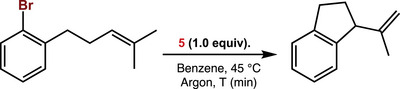
Radical clock experiment confirming that homolytic Ar–Br bond cleavage is initiated from complex **5**.

To further probe the involvement of a homolytic bond cleavage pathway, we performed a radical clock experiment inspired by the work of Hartwig and co‐workers (Scheme 4) [69]. Specifically, treatment of **5** with 1‐bromo‐2‐(4‐methylpent‐3‐enyl)benzene resulted in rapid intramolecular cyclization, as evidenced by diagnostic signals in the ^1^H NMR spectrum and GC‐MS analysis, consistent with a radical‐mediated 5‐exo‐trig ring closure (Figures S29,S30, S33, and S34). Concurrently, ^31^P NMR and ^1^H NMR revealed the formation of mono‐aryl complex **3**, along with additional high‐spin Fe (II) species (Figures S31 and S32). These findings strongly support a mechanism involving homolytic bond cleavage with the concomitant formation of an aryl radical that is subsequently trapped by the formed Fe(I) species after reductive elimination.

### 2.4 Iron Speciation during Catalysis and Mechanism

With the key elementary steps of the aryl–aryl cross‐coupling mechanism established, we next aimed to elucidate the iron speciation under catalytic conditions. In particular, in situ analysis of the iron speciation in the presence of both the nucleophile and electrophile could reveal transient intermediates not observed during our stoichiometric experiments. Moreover, these studies might also offer additional mechanistic insight into the identity and electronic structure of the catalytically active species.

**FIGURE 5:**
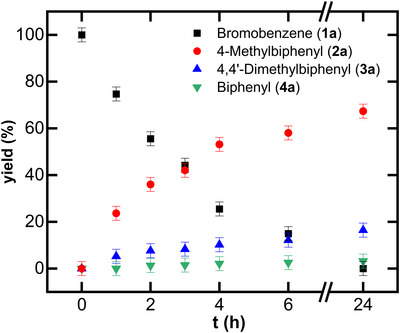
Kinetic profile of the herein reported aryl–aryl Kumada cross‐coupling reaction catalyzed by complex **2** (5 mol%).

We started our investigation by analyzing the product distribution by GC/MS as a function of time (Figure 5). As evident from Figure 5, the data show a steady exponential decrease in substrate and a corresponding increase in cross‐coupled product, with no observable induction period. Notably, ∼25% conversion to the cross‐coupled product is achieved after 1 h, and ∼50% after 4 h.

The total yield of cross‐coupled product (4‐methylbiphenyl) reaches ∼70% after 24 h. Homo‐coupling of the electrophile is minimal throughout the course of the reaction, while homo‐coupling of the nucleophile accounts for ∼17% after 24 h.

**FIGURE 6:**
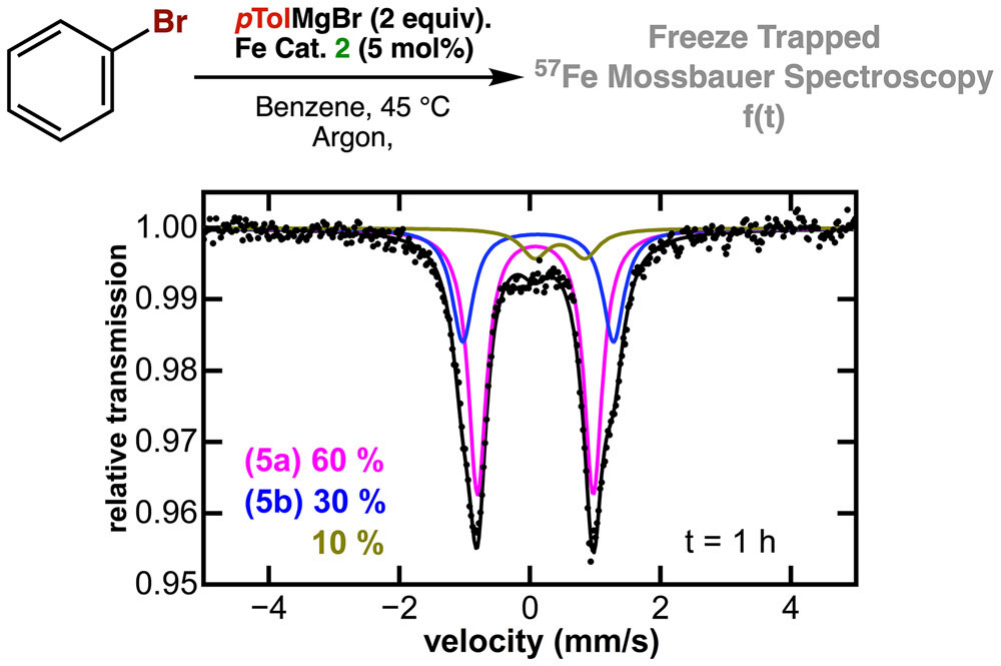
Freeze‐trapped 80 K ^57^Fe Mössbauer spectra of the iron speciation during catalysis with complex 2 after 1 h.

The obtained kinetic profile of the cross‐coupling reaction enabled us to probe the iron speciation at specific time points. Freeze‐trapped ^57^Fe Mössbauer spectra were recorded after 1 and 2 h (Figures 6 and S49). After 1 h, the spectra display two dominant species assigned to **5a** (*δ* = 0.07 mm/s, |Δ*E*_Q_| = 1.78 mm/s) and **5b** (*δ* = 0.12 mm/s, |Δ*E*_Q_| = 2.31 mm/s), together accounting for approximately 90% of the total iron in solution. A minor signal corresponding to a trace impurity was also detected. As the reaction proceeded, up to roughly 40% product formation (2 h), no appreciable spectral changes were observed, indicating that **5a**/**5b** remain the dominant iron species throughout this period. The absence of any high‐spin iron complexes further suggests complete and efficient transmetalation under the experimental reaction conditions. These observations are fully consistent with our stoichiometric studies (*vide supra*) and with previous reports identifying bis‐aryl iron species as the catalyst resting state in iron‐catalyzed C(sp^2^)–C(sp^3^) cross‐coupling reactions. Unfortunately, at longer reaction times (>4 h), extensive accumulation of magnesium salts both diluted the active species and interfered with γ‐ray transmission, preventing reliable Mössbauer measurements. Collectively, these data indicate that the bis‐aryl species **5** is the predominant intermediate under catalytic conditions, and that homolytic Ar–Br bond cleavage of the electrophile constitutes the rate‐limiting step.

**SCHEME 5:**
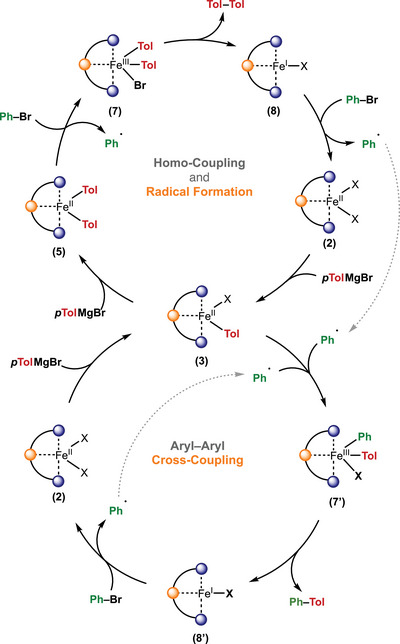
Plausible mechanism for the herein reported iron‐catalyzed aryl‐aryl Kumada cross‐coupling.

With a detailed understanding of the individual mechanistic steps, we can now propose a comprehensive catalytic cycle for the herein reported iron‐catalyzed aryl–aryl Kumada cross‐coupling. The mechanism can be divided into two key parts: (i) activation of the Fe(II) pre‐catalyst **2** to generate the active Fe(I) species **8**, and (ii) productive cross‐coupling of the aryl nucleophile with the aryl electrophile, mediated by heteroleptic Fe(III)–aryl species (Scheme 5).

Activation of the pre‐catalyst is achieved by sequential transmetalation of the Fe(II) pre‐catalyst **2** with two equivalents of *p*‐tolylmagnesium bromide (*p*TolMgBr), affording first the Fe(II) mono‐aryl species **3**, followed by formation of the bis‐aryl Fe(II) complex **5**. Subsequently, the bis‐arylated Fe(II) species [(PC_NHC_P)Fe(Ar)_2_] plays a central role in promoting homolytic Ar–Br bond cleavage of the electrophile to generate the first phenyl radical and a high‐valent Fe(III)(Tol)_2_Br intermediate (**7**). In principle, capture of the phenyl radical by residual **5** could also lead to formation of a tris‐aryl Fe(III) species, Fe(III)(Ar)_2_(Ph). However, reductive elimination from such an intermediate would be expected to afford a statistical mixture of homo‐ and cross‐coupled products, which is not observed experimentally. Instead, reductive elimination from **7** directly furnishes the Fe(I) halide species **8**, which serves as a key catalytic intermediate from which two distinct mechanistic scenarios can evolve (Scheme 5 and Figure S54). In the first scenario, Fe(I)Br (**8**) reacts with the aryl electrophile, regenerating Fe(II) dihalide (**2**) while generating an aryl radical through homolytic C–Br bond cleavage. Such Fe(I)‐mediated halide activation is well documented in iron‐catalyzed cross‐coupling chemistry, and our DFT calculations (*vide infra*) show that this process occurs with a significantly lower activation barrier than from the Fe(II) bis‐aryl species (**5**). The high barrier for homolytic cleavage from Fe(II) rationalizes why **5** is the dominant iron species observed under catalytic conditions and identifies this step as rate‐limiting in the overall reaction. Once formed, **2** undergoes rapid transmetalation with *p*TolMgBr to generate the mono‐aryl Fe(II) complex (**3**), which captures the aryl radical to yield Fe(III)(Ph)(Tol)Br (**7’**). Reductive elimination from **7’** produces the cross‐coupled product (Ph–Tol) and regenerates Fe(I)Br (**8’**), completing the catalytic cycle (Scheme 5). The key feature of this mechanistic framework is the presence of two interconnected catalytic pathways: (i) an initiation cycle, in which the Fe(II) bis‐aryl species generates the first aryl radical through oxidative homolytic PhBr bond cleavage, that accounts for homo‐coupled product formation, and (ii) a propagation cycle, in which the Fe(I) species sustains catalytic turnover by continuously generating aryl radicals to produce the cross‐coupled product.

In the second mechanistic scenario, Fe(I)Br (8) does not activate the electrophile directly but instead captures the phenyl radical to form the mono‐aryl Fe(II) complex Fe(II)(Ph)Br (**3’**). Subsequent transmetalation with *p*‐TolMgBr affords the heteroleptic Fe(II)(Ph)(Tol) species (**5**’), which then reacts with a second equivalent of PhBr through an MgBr_2_‐assisted homolytic Ar–Br bond cleavage to generate Fe(III)(Ph)(Tol)Br species **7′**. Reductive elimination from **7′** furnishes the cross‐coupled product (Ph–Tol) and regenerates Fe(I)Br (**8**), thereby closing the propagation pathway. Although **5′** could, in principle, also engage in radical capture, reductive elimination from the resulting tris‐aryl Fe(III) species would be expected to yield homo‐coupled electrophile, a reactivity pattern that is not observed under the catalytic conditions and is therefore excluded. Consequently, the defining feature of this pathway is that Fe(I)Br (**8**) functions both as the effective radical trap and as the conduit for re‐entry into the catalytic manifold (Figure S54). In contrast to the first scenario, where the initial aryl radical is generated through an energetically demanding homolytic Ph–Br bond cleavage step, here, the same bond activation event governs productive cross‐coupling within the propagation cycle. As a result, this pathway, while mechanistically distinct, is overall less favorable than the proposed pathway in Scheme 5.

Notwithstanding, both mechanistic scenarios are consistent with our experimental findings and align with emerging paradigms in iron‐catalyzed cross‐coupling. The dual role of a low‐valent Fe(I) species, either as a radical initiator in the first scenario or as a propagator in the second, is well‐supported by the seminal studies of Nakamura, Neidig, and Bedford. Moreover, the involvement of Fe(III) in reductive elimination mirrors mechanistic proposals for Fe/Xantphos‐catalyzed alkyl–alkyl couplings, underscoring the broader relevance of this Fe(I)/Fe(III) redox interplay across diverse iron‐mediated transformations. The mechanism proposed herein thus reinforces the growing consensus that iron‐catalyzed C(sp^2^)–C(sp^2^) bond formation proceeds through an Fe(I)/Fe(II)/Fe(III) redox manifold that unites oxidative homolytic bond activation with a genuine two‐electron reductive elimination. In this context, our PC_NHC_P system broadens the mechanistic landscape of iron‐mediated cross‐coupling by demonstrating that a pincer‐ligated Fe catalyst can access and stabilize this Fe(I)/Fe(III) redox interplay during aryl–aryl coupling, effectively bridging the reactivity gap between base‐metal and precious‐metal catalysis while retaining the distinct open‐shell character of iron.

### 2.5 Computational Mechanistic Studies

To gain deeper insight into the mechanism of the iron‐catalyzed aryl–aryl cross‐coupling, we employed density functional theory (DFT) calculations to further complement our experimental studies. As outlined above, the mechanism comprises two key stages: (i) homo‐coupling and radical initiation and (ii) productive cross‐coupling mediated by an Fe(I) mono‐aryl intermediate according to Scheme 5.

### 2.6 Iron Catalyzed Homo‐Coupling and Radical Initiation

**SCHEME 6:**
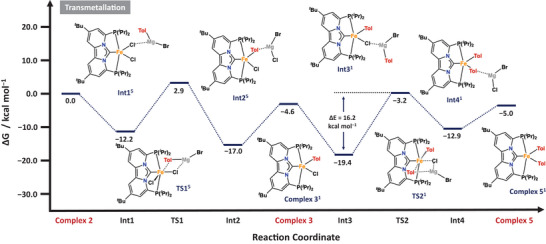
Calculated free energy profile (Δ*G*) in kcal mol^−1^ for the formation of the iron bis‐arylated species **5**, via a stepwise transmetalation process from complex **2** with *p*‐tolylmagnesium bromide. See the Supporting Information for more computational details.

We initiated our DFT study from [(PC_NHC_P)FeCl_2_] (**2**), which exhibits a quintet ground state. The first transmetalation with *p*TolMgBr proceeds via an encounter complex (**Int1^5^**) between complex **2^5^** and *p*TolMgBr, which is overall downhill in energy by 12.2 kcal mol^−1^ (Scheme 6). The subsequent transition state (**TS1^5^**) leads to the MgBr_2_ adduct **Int2^5^** with an overall activation barrier of Δ*G* ^‡^ = 15.1 kcal mol^−1^. The low activation barrier is consistent with the experimentally observed rapid formation of mono‐aryl complex **3^1^**. Furthermore, the involvement of Mg adducts has also been proposed by Nakamura and co‐workers for related FeF_3_‐catalyzed aryl–aryl cross coupling reactions. Dissociation of MgBr_2_ affords complex **3^1^**, rendering the first transmetalation overall exergonic by 4.6 kcal mol^−^^1^ (Scheme 6). Notably, this step is accompanied by a spin‐state change, with the ground state of the iron center switching from quintet in **2** to singlet in **3**. Since the transition‐state for transmetalation is relatively early, the change from high‐spin Fe(II) to low‐spin Fe(II) occurs en route to **Int2^1^**, which exhibits a singlet ground‐state.

The second transmetalation follows an analogous pathway (Scheme 6). The computed transition‐state barrier of Δ*G* ^‡^ = 16.2 kcal mol^−^^1^ (**TS2^1^**) for transmetalation is slightly higher than for the first step, reflecting increased steric congestion around the metal center. This trend aligns well with our experimental observations and other reported literature, which indicate that the second transmetalation is generally more challenging than the first. Overall, conversion of **2^5^** to the diaryl species **5^1^** is exergonic by −5.0 kcal mol^−^^1^, where the low transition state barriers corroborate the experimentally observed rapid formation of the bis‐aryl intermediate. Importantly, these calculations also provide a direct rationale for our Mössbauer and kinetic studies, which consistently identified bis‐aryl complexes as the dominant resting state under catalytic conditions.

**SCHEME 7:**
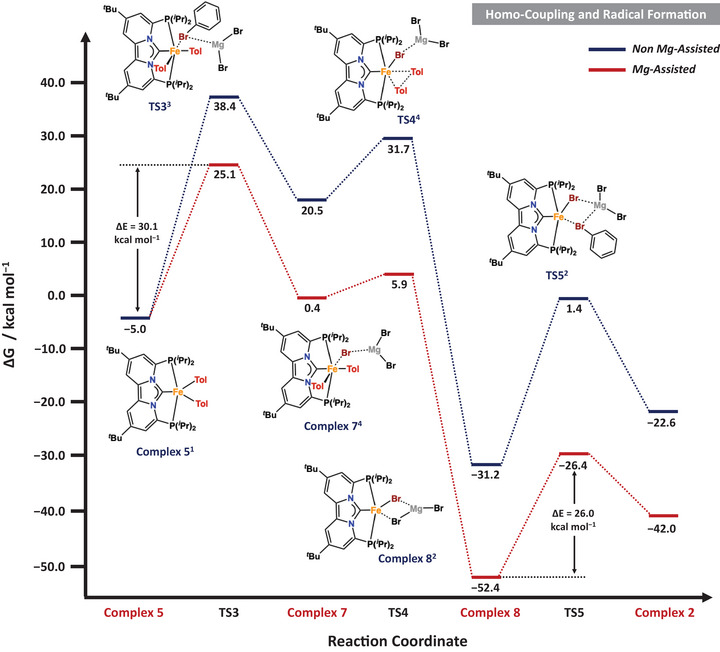
Calculated free energy profile (Δ*G*) in kcal mol^−1^ for iron catalyzed homo‐coupling and initial radical formation. Reported energies are relative to the energy of **complex 2^5^** at 0.0 kcal/mol. See the Supporting Information for more computational details.

**SCHEME 8:**
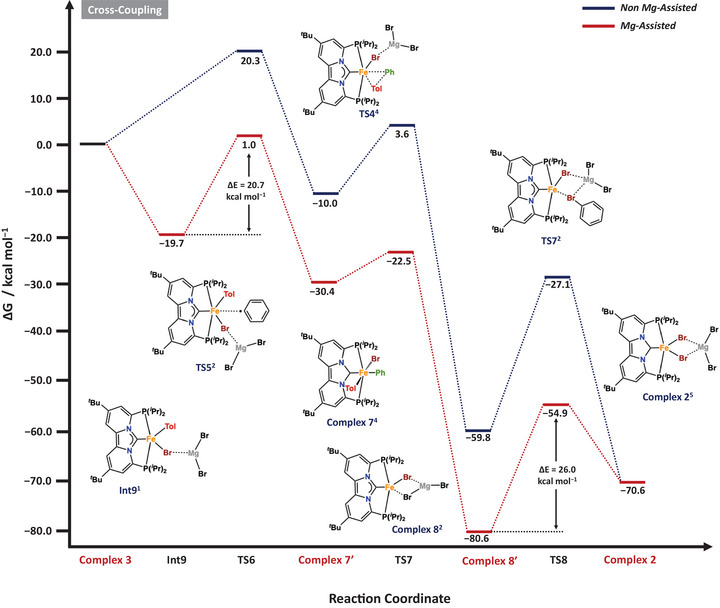
Calculated free energy profile (Δ*G*) in kcal mol^−1^ for the iron catalyzed aryl‐aryl cross‐coupling. Reported energies are relative to the energy of **Complex 3** set at 0.0 kcal/mol. See the Supporting Information for more computational details.

Upon formation of the bis‐aryl complex **5**, homolytic cleavage of the Ph–Br bond, to generate an aryl radical, occurs via **TS3^3^** with a transition state barrier of Δ*G* ^‡^ = 43.4 kcal mol^−^^1^, which is prohibitively high under the experimental reaction conditions (Scheme 7, **blue trace**). Inclusion of magnesium salts (e.g., MgBr_2_) substantially lowers this barrier by ~13.3 kcal mol^−^^1^ (Scheme 7, **red trace**). Although the resulting transition state barrier of ∼30 kcal mol^−^^1^ remains considerable, it is comparable to values reported for other iron‐mediated homolytic bond activation processes and is consistent with our experimental observation that the bis‐aryl species **5** acts as the resting state during catalysis. It is important to note that the same homolytic C–Br bond cleavage from Fe(I)Br (**8’**) in the cross‐coupling cycle is calculated to occur with a significantly lower MgX_2_‐assisted barrier (Scheme 8 **;** Δ*G* ^‡^ = 26.0 kcal mol^−^^1^), highlighting the distinct reactivity within the propagation pathway.

Following the homolytic bond cleavage, the nascent aryl radical escapes the radical cage, yielding the magnesium‐stabilized Fe(III) derivative of **complex 7^4^**, which is best described as an intermediate spin Fe(III) (*S* = 3/2) species; [(PC_NHC_P)Fe(Ar)_2_Br][MgBr_2_]. From this intermediate, reductive elimination occurs with a nearly negligible barrier **(TS4^4^** Δ*G* ^‡^ = 5.5 kcal mol^−^^1^), affording the low‐spin (*S* = 1/2) Fe(I) derivative of complex, [(PC_NHC_P)FeBr][MgBr_2_] (**8**), together with the organic homo‐coupled bis‐aryl product (Scheme 7). Finally, Mg‐assisted homolytic cleavage of the C–Br bond from a second aryl electrophile proceeds with a transition‐state barrier of ΔG^‡^ = 26.0 kcal mol^−^^1^, regenerating the Fe(II) dihalide species **Complex 2** ^5^ that subsequently re‐enters the catalytic cycle via transmetalation (*vide supra*). These results also explain the formation of the iron dihalide in stoichiometric reactions between complex **5** and the aryl electrophile. The concurrent observation of mono‐aryl complex **3** in these experiments can be rationalized by aryl radical capture of Fe(I)Br complex **8**, which remains present under stoichiometric conditions. Depending on the kinetic regime, Fe(I)Br (**8**) can therefore participate in two complementary processes, either (i) by reaction with the aryl electrophile to regenerate the Fe(II) dihalide, or (ii) by radical capture to yield the Fe(II) mono‐aryl species **3**. Both pathways ultimately converge to the same bis‐aryl intermediate after successive transmetalation steps. Importantly, these calculations also elucidate the previously unclear role of magnesium salt additives. Coordination of MgBr_2_ to the iron center markedly lowers the activation barrier for homolytic C–Br bond cleavage, which is the rate‐limiting step in most iron‐catalyzed cross‐coupling reactions. These findings indicate that homolytic bond activation is more favorable from an “ate”‐like Fe(I) intermediate, stabilized by Mg^2^^+^, than from a genuine neutral Fe(I) halide species.

The herein proposed mechanistic picture is consistent with earlier proposals for iron‐catalyzed cross‐coupling, in which Nakamura and co‐workers highlighted the critical role of Mg salts in promoting carbon–halide bond activation, while Neidig and colleagues identified high‐spin Fe(III) species as key precursors to reductive elimination in C(sp^2^)–C(sp^3^) systems. Our results extend these concepts to aryl–aryl bond formation, providing rare combined experimental and computational evidence for an Fe(I)/Fe(II)/ Fe(III) redox manifold in iron‐mediated biaryl coupling. In sharp contrast to the classical two‐electron Pd(0)/Pd(II) or Ni(0)/Ni(II) cycles, the iron pathway relies on radical intermediates and spin‐state crossings, underscoring the distinct reactivity profile of base‐metal catalysis.

### 2.7 Iron Catalyzed Cross‐Coupling

Having established the formation pathway of the initial radical, our attention next turned to elucidating the mechanism of the cross‐coupling step (Scheme 8). Central to this process is complex **3**, which may either undergo transmetalation to afford complex **5** or capture an aryl radical to initiate cross‐coupling. The reaction is thus triggered by the addition of the aryl radical to the Fe(II) center of **3^1^**, leading to oxidation of the metal and formation of the mixed‐aryl Fe(III) intermediate [(PC_NHC_P)Fe(Ar)(Ph)Br] (**7’^4^**), which is a structural analog of the bis(aryl) complex **7^4^** (Ph vs. Tol). The computed activation barrier for this radical capture step is Δ*G* ^‡^ = 20.7 kcal mol^−^^1^ and approximately 4 kcal mol^−^^1^ higher than that of the competing transmetalation pathway. Following formation of Fe(III) species **7’^4^**, cross‐coupling and radical propagation proceed via steps analogous to those established for the homo‐coupling pathway. In line with that mechanism, Fe(I) species **8’^2^** is generated through reductive elimination of the cross‐coupled bis(aryl) product from the heteroleptic Fe(III) complex **7’^4^**, a process associated with a modest transition state barrier of Δ*G* ^‡^ = 5.5 kcal mol^−^^1^ (TS7). Radical propagation is then initiated by homolytic bond cleavage of the aryl electrophile, regenerating the Fe(II) dihalide species through a rate‐limiting transition state with Δ*G* ^‡^ = 26.0 kcal mol^−^^1^ (Scheme 8 **;** TS8). As shown previously, the dihalide complex can readily undergo transmetalation to regenerate the mono‐aryl species (III) (Scheme 6), thereby closing the catalytic cycle. The key distinction between the homo‐ and cross‐coupling pathways arises from the energetics of their respective energy spans, which are calculated as 44.5 kcal mol^−^^1^ and 26.0 kcal mol^−^^1^ for radical initiation (ΔΔ*G* = **Int3^1^** − **TS3^3^**) and radical propagation (ΔΔ*G* = **8^2^ – TS8^2^**), corresponding to the Fe(II) and Fe(I) mediated homolytic bond‐cleavage processes. Consequently, only a small fraction of the mono‐aryl intermediate **3** needs to enter the cross‐coupling manifold to sustain catalysis, consistent with the predominant formation of bis(aryl) products under catalytic conditions. (Scheme 5). The alternative mechanistic pathway (*vide supra*), in which cross‐coupling is initiated by homolytic C–Br bond cleavage from the Fe(II) bis‐aryl species, is depicted in Figure S54.

Overall, the present work advances iron‐catalyzed cross‐coupling with pincer‐based ligands, outlining a well‐defined mechanistic roadmap containing guiding principles for controlling open‐shell pathways on the low‐spin manifold. Our strategy of using bespoke strong‐field PC_NHC_P pincer ligands offers a foundation for expanding iron catalysis to more diverse substrates and reaction classes. As such, this mechanistic understanding will be critical for the rational design of next‐generation sustainable cross‐coupling systems, which are under current evaluation.

## 3 Conclusions

In summary, this work provides a detailed mechanistic framework for the iron‐catalyzed aryl–aryl Kumada cross‐coupling mediated by the PC_NHC_P pincer complex [(PC_NHC_P)FeCl_2_]. Through a combination of multinuclear NMR spectroscopy, freeze‐trapped ^5^ ^7^Fe Mössbauer analysis, single‐crystal x‐ray diffraction, kinetic studies, and DFT computations, we have identified and characterized key intermediates, including mono‐ and bis‐aryl Fe(II) complexes, and have established that bis‐aryl species represent the dominant catalyst resting state under catalytic conditions.

Mechanistically, the cross‐coupling proceeds through three key steps: (i) sequential transmetalation of the Fe(II) pre‐catalyst to generate a bis‐aryl Fe(II) intermediate, (ii) homolytic Ar–Br bond activation leading to aryl‐radical formation, and (iii) reductive elimination from a high‐spin Fe(III) species to furnish the cross‐coupled product together with an Fe(I) complex. Notably, this reductive elimination step represents a rare example of direct C(sp^2^)–C(sp^2^) bond formation at an iron center. The resulting Fe(I) halide species acts as the crucial catalytic linchpin, serving both as the initiator of radical formation and as the propagator of subsequent turnover through Mg^2^^+^‐assisted homolytic bond activation.

These findings provide direct experimental and computational evidence for an Fe(II)/Fe(III)/Fe(I) redox‐manifold in iron‐catalyzed aryl–aryl cross‐coupling, which is fundamentally distinct from the classical two‐electron Pd(0)/Pd(II) and Ni(0)/Ni(II) pathways in traditional cross‐coupling reactions. The necessity for spin‐state changes and radical intermediates highlights the unique reactivity of iron, while the key role of Mg^2^^+^ salts in facilitating C–Br bond cleavage underscores the importance of secondary coordination effects in modulating reactivity. By integrating radical and organometallic steps, the proposed mechanism aligns iron catalysis more closely with established cross‐coupling paradigms, while simultaneously showcasing its unique reactivity.

## Author Contributions

**Michael L. Neidig, and Graham de Ruiter**: conceptualization. **Jatin Panda, Magali Gimeno, Zeqing Chen, Subhash Garhwal, and Laura Levy**: methodology. **Jatin Panda, Magali Gimeno, Zeqing Chen, Subhash Garhwal, and Laura Levy**: investigation. **Jatin Panda, Magali Gimeno, Alexander Kaushansky, Renana Gershoni‐Poranne, Michael L. Neidig, and Graham de Ruiter**: writing—original draft. **Renana Gershoni‐Poranne, Michael L. Neidig, and Graham de Ruiter**: visualization. **Amrita Gogoi., Alexander Kaushansky, and Renana Gershoni‐Poranne**: computation. **Renana Gershoni‐Poranne, Michael L. Neidig, and Graham de Ruiter**: funding acquisition. **Magali Gimeno, Jatin Panda, Jos Briggs‐Pritchard, and Michael L. Neidig**: spectroscopy. **Renana Gershoni‐Poranne, Michael L. Neidig, and Graham de Ruiter**: resources. **Renana Gershoni‐Poranne, Michael L. Neidig, and Graham de Ruiter**: supervision. The final manuscript was written through contributions of all authors. All authors have given approval to the final version of the manuscript.

## Conflicts of Interest

The authors declare no conflict of interest.

## Supporting information




**Supporting File 1**: The data underlying this study are available at the published article and its Supporting Information. The authors have cited additional references within the Supporting Information.

**Supporting File 2**: anie71772‐sup‐0002‐Data.zip.

## Data Availability

The data that support the findings of this study are available from the corresponding author upon reasonable request.
