# Supplementary material for: Iron Catalyzed Aryl–Aryl Kumada Cross‐Coupling: A Mechanistic and Computational Investigation
Source: Angew Chem Int Ed Engl. 2026 Mar 13;65(17):e3094782. doi: 10.1002/anie.3094782 (PMC13098315; doi:10.1002/anie.3094782)
Supplement: Supplementary file 1 — Supporting File 1: The data underlying this study are available at the published article and its Supporting Information. The authors have cited additional references within the Supporting Information. [file ANIE-65-e3094782-s002.pdf]

# Supporting Information

# Iron Catalyzed Aryl-Aryl Kumada Cross-Coupling: A Mechanistic and Computational Investigation

Jatin Panda,[a]+ Magali Gimeno,[b]+ Amrita Gogoi,[a] Zeqing Chen,[a] Subhash Garhwal,[a] Laura Levy,[a] Alexander Kaushansky,[a] Natalia Fridman, [a] Jos Briggs-Pritchard,[b] Renana Gershoni-Poranne,[a][c]* Michael L. Neidig[b]* and Graham de Ruiter[a][c]*

*[a]Schulich Faculty of Chemistry, Technion – Israel Institute of Technology, Technion City, 3200008 Haifa, Israel. [b]Inorganic Chemistry Laboratory, University of Oxford, South Parks Road, OX1 3QR, Oxford, United Kingdom. [c]The Resnick Sustainability Center for Catalysis, Technion – Israel Institute of Technology, Technion City, 3200008 Haifa, Israel.*

## Table of Contents

**Experimental Section** .......... **S3**
General Considerations .......... S3
Physical Methods .......... S3
Mössbauer Spectroscopy .......... S3
X-Ray Crystallography .......... S4
Synthetic Procedures .......... S5
Synthesis of [(PC$_{NHC}$P)Fe(Cl)(Tol)] (**3**) .......... S5
NMR Spectra .......... S7
Mössbauer Spectra .......... S15
Radical Trapping Experiments .......... S16
The Reaction of Complex **2** with Nucleophile (pTolMgBr or PhLi) .......... S28
Reaction of Complexes **5a**/**5b** with Electrophile .......... S39
Procedure for the Catalytic Cross-Coupling. .......... S40
Analysis of Product Yield by Gas Chromatography .......... S42
X-Ray Crystallography .......... S44

**Computational Details** .......... **S48**
General Computational Details .......... S48
Input Templates .......... S49
Benchmarking .......... S51
Energetics of the Proposed Catalytic Cycle .......... S56
Energetics of the Alternative Mechanistic Proposal .......... S58

## Experimental Section

### General Considerations

All chemical reagents and solvents were purchased from commercial sources. All air and moisture-sensitive manipulations were carried out in an MBraun inert-atmosphere [$N_2$ or Argon(Ar) according to the required experiment] dry box equipped with a direct liquid nitrogen inlet line. Glassware was oven dried at 150 °C for at least 2h prior to use and allowed to cool under vacuum. All anhydrous solvents were further dried using activated alumina/4Å molecular sieves and stored under $N_2$ or Ar atmosphere over 4Å molecular sieves. The $PC_{NHC}P$ carbene, and [($PC_{NHC}P$)$FeCl_2$] (**1**),[1] $KC_8$,[2] [(($PC_{NHC}P$)Fe($N_2$))$_2$-μ-$N_2$)],[3] 1-bromo-2-(4-methylpent-3-en-1-yl) benzene,[4] 1-Bromo-2-[(3-methyl-2-buten-1-yl) oxy] benzene,[5] Aryl Grignards,[6] *p*Tolyl lithium,[7] was synthesized according to literature procedures. The $^{1}H$, $^{13}C$, and $^{31}P$ NMR spectra were recorded on Bruker AVANCE III, 300, 400, 500 and 600 NMR spectrometers at room temperature unless mentioned otherwise. All chemical shifts (δ) are reported in ppm, and coupling constants (J) are in Hz. The $^{1}H$ and $^{13}C\{^{1}H\}$ NMR spectra were referenced using residual solvent peaks in the deuterated solvent. The $^{31}P$ chemical shifts are reported relative to the internal lock signal. Deuterated solvents ($CDCl_3$, benzene-$d_6$, toluene-$d_8$ and THF-$d_8$) were purchased from Cambridge Isotope Laboratories, dried over calcium hydride, degassed by three freeze-pump- thaw cycles and vacuum-transferred prior to use. $^{57}FeCl_2$, $^{57}FeBr_2$, and the corresponding THF adduct salts were synthesized following literature procedures[8] starting from $^{57}Fe$ metal (95% enriched) purchased from Isoflex. Temperature dependent reactions (45 °C) were performed in the glovebox using a Huber ministat 230-cc-NR recirculating bath combined with a Syrris hot/cold plate fit with a PT100 thermocouple for direct measurement and control of the reaction temperature. Dry gloveboxes equipped with cold wells provided additional infrastructure for low-temperature reactions and manipulations (- 80 to - 100 °C). Appropriate solvent-dry ice or liquid $N_2$ formulations were utilized to achieve temperatures as low as - 80 °C in the cold well. GC-MS measurements were done in an Agilent 5977B GC/MSD (University of Oxford). Elemental Analysis were performed by Kolbe Microanalytical laboratory in Oberhausen (Germany).

### Physical Methods

### Mössbauer Spectroscopy

All samples were prepared under either Argon or Nitrogen atmosphere in a glovebox equipped with a liquid nitrogen fill port to enable sample freezing at 77 K within the glovebox, or in the cold well at liquid nitrogen temperatures in the case of the Ar atmosphere glovebox. Samples were loaded in Derlin Mössbauer sample cups and subsequently frozen in liquid $N_2$ or frozen in the cold well. Zero field 80 K Mössbauer

measurements were performed using a SeeCo MS4 Mössbauer spectrometer integrated with a Janis SVT-400 T He/$N_2$ cryostat. Isomer shifts reported were determined relative to α-Fe at 298 K. In addition, reported $\Delta E_Q$ parameters for zero field correspond to absolute values. All Mössbauer spectra were fit using the program WMoss (SeeCo). Freeze-trapped solution Mössbauer samples were prepared utilizing $^{57}$Fe enriched catalyst $PCPFeCl_2$. Experiments were carried out under catalytically relevant conditions as reported for the method (Fe concentration, temperature, and solvent). Solid Mössbauer samples were prepared by isolating crystalline material from crystallization procedures performed starting from $PCPFeCl_2$.

**X-Ray Crystallography**

For compounds **3**, **4** and **6** low temperature (100 K) diffraction data were collected using a Rigaku XtaLab$^{Pro}$ X-ray diffractometer equipped with a 4-circle Kappa goniometer, a Pilatus Dectris 3R S200K-A detector, and a micro-focus sealed tube with microCMF-VHF. The data were collected with either CuKα (λ = 1.54184) radiation (**6**) or MoKα (λ = 0.71073 Å) radiation (**3**, and **4**). All Rigaku diffractometer manipulations, including data collection, integration, and scaling were carried out using the CrysAlisPro 1.171.42.54a software package (Rigaku Oxford Diffraction, 2022). Spherical absorption correction was applied using equivalent radius and absorption coefficients. Empirical absorption correction was applied using spherical harmonics, implemented in the SCALE3 ABSPACK scaling algorithm in CrysAlisPro software package. All structures were solved by direct methods using SHELXS and refined against $F^2$ on all data by full-matrix least squares with SHELXL-2018 using established refinement techniques. All non-hydrogen atoms were refined anisotropically. All hydrogen atoms were included into the model at geometrically calculated positions and refined using a riding model. The isotropic displacement parameters of all hydrogen atoms were fixed to 1.2 times the $U$ value of the atoms they are linked to (1.5 times for methyl groups). All air- and moisture-sensitive manipulations were carried out using standard Schlenk and cannula techniques

**Synthetic Procedures**

**Synthesis of [($PC_{NHC}P$)Fe(Cl)(Tol)] (3)**. In an $N_2$-filled glovebox, to a suspension of [($PC_{NHC}P$)$FeCl_2$ (**1**) (64 mg, 0.1 mmol) in benzene (5 mL) was added, dropwise, a suspension of *p*TolMgBr (0.99 mmol, 99 μL, 1 M solution in THF; the THF was removed under vacuum and the residue re-dissolved in benzene to obtain a THF-free solution) in benzene (5 mL) at room temperature. The resulting reaction mixture was stirred for 1h at 25 °C. During the course of the reaction, the color of the reaction mixture changed from reddish to green and became homogenous. Hereafter, the solvent was concentrated under reduced pressure (2 mL) and filtered through a pad of *Celite*, which was washed with an additional amount of benzene (1 mL). The benzene solution was concentrated under vacuum, and the title compound was obtained as dark green crystals upon crystallization upon slow evaporation of concentrated solution of (**3**) in benzene. Yield: 60 mg (85 %). **$^1$H NMR (600 MHz, $C_6D_6$)**: δ (ppm) 7.21 (s, 2H, *m*-bpy-*H*), 7.16 (m, 4H, **$C_6H_4$** $CH_3$), 6.89 (s, 2H, *m*-bpy-*H*), 3.13 (m, 2H, $(CH_3)_2$C**H**), 2.58 (m, 2H, $(CH_3)_2$C**H**), 2.00 (s, 3H, $C_6H_4$ **$CH_3$**), 1.68 (dd, 6H, (**$CH_3$**)$_2$CH), 1.18 (s, 18H, $^t$Bu), 1.15 (dd, 6H, (**$CH_3$**)$_2$CH), 0.85 (dd, 6H, (**$CH_3$**)$_2$CH), 0.52 (dd, 6H, (**$CH_3$**)$_2$CH). All the peaks were little broadened due to the merging of peaks of both Fe-Br and Fe-Cl complexes. **$^{31}$P {$^1$H} NMR (243 MHz, $C_6D_6$)**: δ (ppm) 82.27 (s). **$^{13}$C NMR (126 MHz, $C_6D_6$)**: δ(ppm) 142.85, 134.79, 128.60, 128.36, 128.17, 127.97, 125.45, 118.66, 112.76, 35.02, 30.34, 26.92, 24.77, 20.48, 19.65, 18.78, 17.93, 17.53. Elemental analysis: Anal. Calcd. for [$C_{38}H_{58}BrFeN_2P_2$]: C, 61.63; H, 7.89; N, 3.78. Found: C, 61.27; H, 7.86; N, 3.68.

**Synthesis of [(($PC_{NHC}P$)Fe(Tol)$_2$$N_2$] (4).** In the $N_2$ filled glovebox, to a suspension of [($PC_{NHC}P$)Fe(Cl)(Tol)] (**3**) (37 mg, 0.05 mmol) in benzene (2 mL) was added drop wise a suspension of *p*TolMgBr (0.049 mmol, 49 μL, 1 M solution in THF; the THF was removed under vacuum and the residue re-dissolved in benzene to obtain a THF-free solution) in benzene (5 mL). The resulting reaction mixture was stirred for 1 h at 25 °C. During the course of the reaction, the color of the reaction mixture changed from green to purple and became homogenous. After 1 hour, the volatiles were removed under reduced pressure and the residue was washed with pentane (2× 2 mL). Hereafter, the residue was dissolved in benzene (2 mL) and filtered through a pad of *Celite*, which was washed with an additional amount of benzene (1 mL). The benzene solution was concentrated under vacuum, and the title compound was obtained as dark purple crystals upon crystallization upon slow evaporation of concentrated solution of (**3**) in benzene. Yield: 34 mg (90 %). *Note: The color of the solution changes under vacuum to green ($N_2$ decoordinated complex).* **$^1$H NMR (600 MHz, $C_6D_6$)**: δ (ppm) 8.88 (d, *J* = 7.7 Hz, 2H, *o*-CH of **$C_6H_4$** $CH_3$), 7.44 (s, 2H, *m*-bpy-*H*), 7.16 (merged with solvent peak, 2H, *m*-CH of **$C_6H_4$** $CH_3$), 6.85 (s, 2H, *m*-bpy-*H*), 6.29 (d, *J* = 6.6 Hz, 2H, *m*-CH of **$C_6H_4$** $CH_3$), 5.30 (d, *J* = 6.6 Hz, 2H, *o*-CH of **$C_6H_4$** $CH_3$), 2.13 (s, 6H,

$C_6H_4$ **$CH_3$**), 2.08 (m, 4H, $(CH_3)_2$**CH**), 1.23 (dd, *J* = 15.6, 6.9 Hz, 12 H, **$(CH_3)_2$**CH), 1.12 (s, 18H, $^t$Bu), 0.81 (dd, *J* = 16.2, 7.2 Hz, 12H, **$(CH_3)_2$**CH). **$^{31}$P {$^1$H} NMR (243 MHz, $C_6D_6$)**: δ (ppm) 95. 20 (s). **$^{13}$C NMR (126 MHz, $C_6D_6$)**: δ (ppm) 144.00, 143.28, 142.07, 138.92, 137.51, 136.68, 129.79, 128.59, 128.35, 128.16, 127.97, 127.45, 127.41, 127.24, 121.27, 112.36, 34.98, 30.26, 26.90, 21.07, 20.98, 20.22, 18.92. Elemental analysis: Anal. Calcd. for [$C_{45}H_{65}FeN_2P_2$]: C, 71.89; H, 8.71; N, 3.73. Found: C, 71,61; H, 8,63; N, 3,65.

**Synthesis of [(($PC_{NHC}P$)Fe(Tol)$_2$] (5).** In an Argon-filled glovebox, in a 4 mL scintillation vial, [($PC_{NHC}P$)$FeCl_2$] (1) (0.042 mmol, 27 mg) was dissolved in benzene (1 mL) at 45 °C, followed by addition of 2.0 equivalents of *p*TolMgBr (0.084 mmol) in benzene (1 mL). The reaction was stirred for 1 hour at RT, filtered through *Celite,* whereafter the *Celite* plug was washed with an extra amount of benzene (1 mL). The benzene solution was concentrated under vacuum, and the title compound was obtained as dark green crystals upon crystallization upon slow evaporation of concentrated solution of (**5**) in benzene. Yield: 25 mg (78%). **$^1$H NMR (500 MHz, $C_6D_6$)** δ 7.20(s, 2H, *m*-bpy-*H*), 6.83(s, 2H, *m*-bpy-*H*), 6.79(d, *J* = 5 Hz, 4H, **$C_6H_4$** $CH_3$), 6.56(d, *J* = 10 Hz, 4H, **$C_6H_4$** $CH_3$), 2.56(m, 4H, $(CH_3)_2$**CH**), 2.20(s, 6H, $C_6H_4$ **$CH_3$**), 1.20(s, 18H, $^t$Bu), 1.07(dd, 12H, **$(CH_3)_2$**CH), 0.84(dd, 12H, **$(CH_3)_2$**CH). **$^{31}$P{$^1$H} NMR (202 MHz, $C_6D_6$)** δ 83.47. **$^{13}$C NMR (126 MHz, $C_6D_6$)** δ 142.55, 134.91, 129.80, 128.36, 128.16, 127.97, 127.23, 126.13, 124.89, 117.22, 112.30, 35.03, 34.95, 30.40, 26.19, 25.13, 21.15, 18.34.

## NMR Spectra

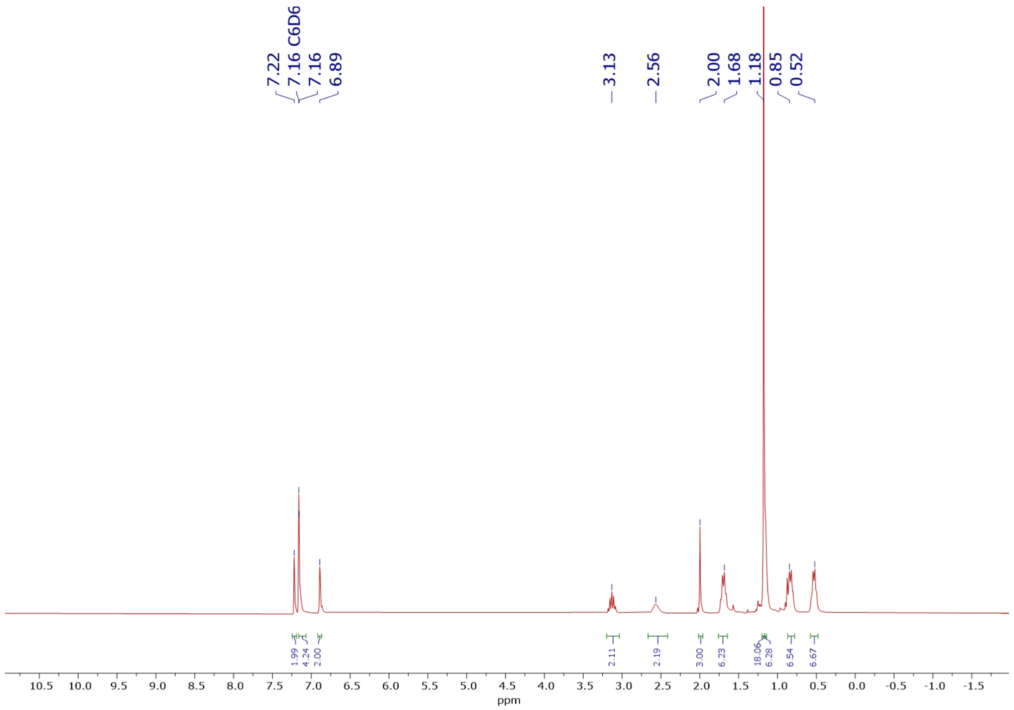


**Figure S1.** $^1$H NMR spectrum (600 MHz) of **3** in benzene-$d_6$.

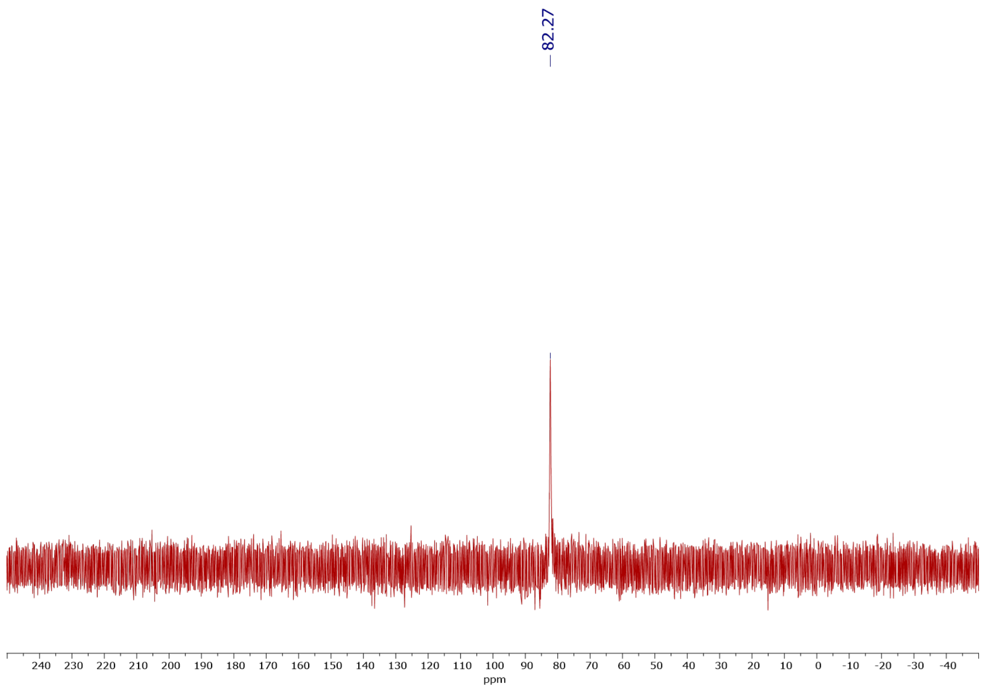


**Figure S2.** $^{31}$P {$^1$H} NMR spectrum (243 MHz) of **3** in benzene-$d_6$.

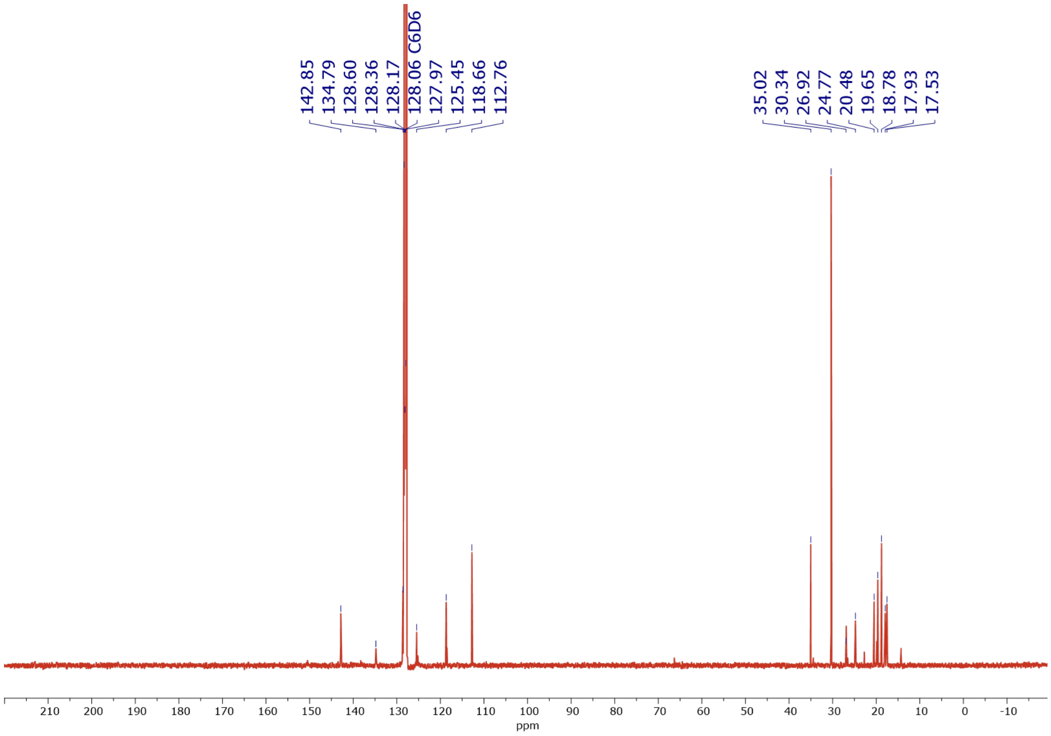


**Figure S3.** $^{13}C\{^{1}H\}$ NMR spectrum (126 MHz) of **3** in benzene-$d_6$.

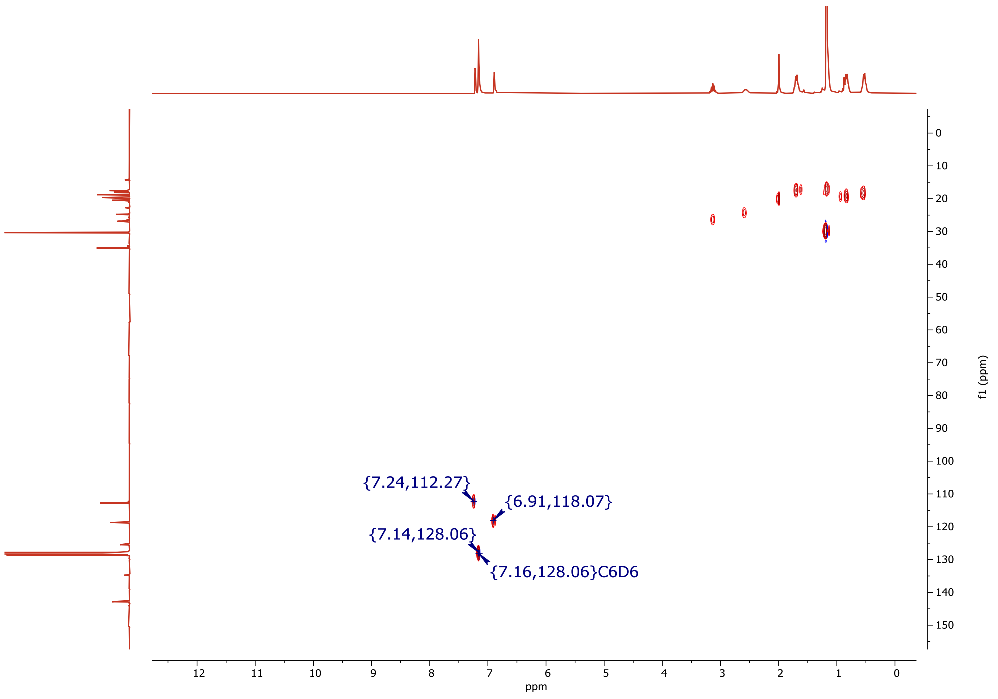


**Figure S4.** HSQC NMR spectrum (500 MHz) of **3** in benzene-$d_6$.

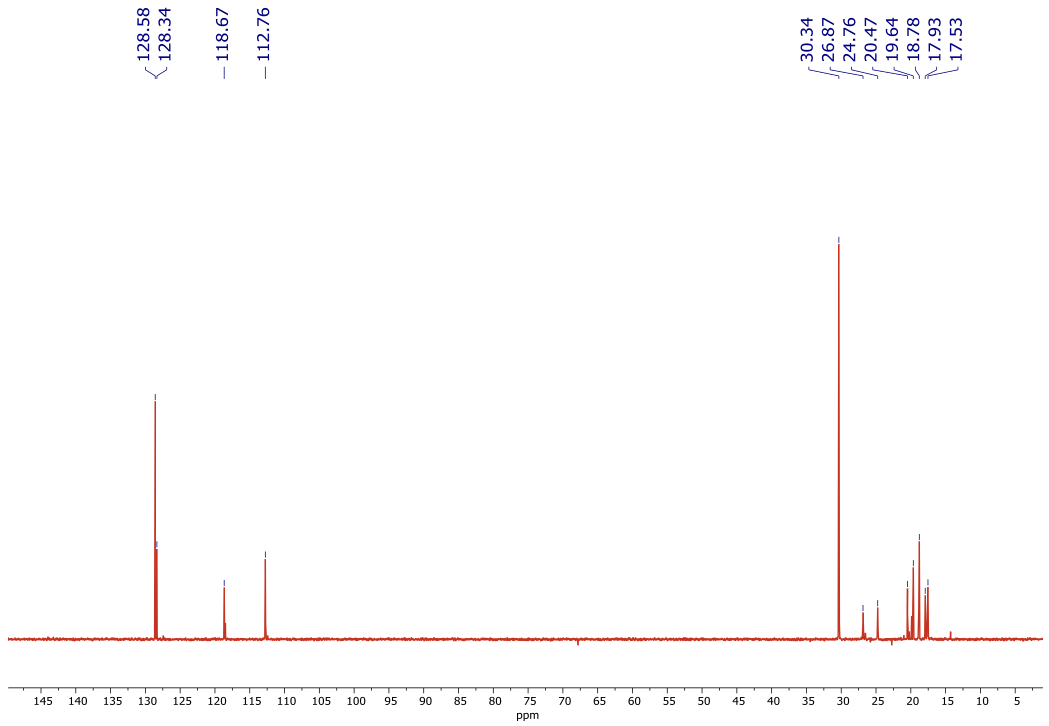


**Figure S5.** DEPT-135 spectrum (500 MHz) of **3** in benzene-$d_6$.

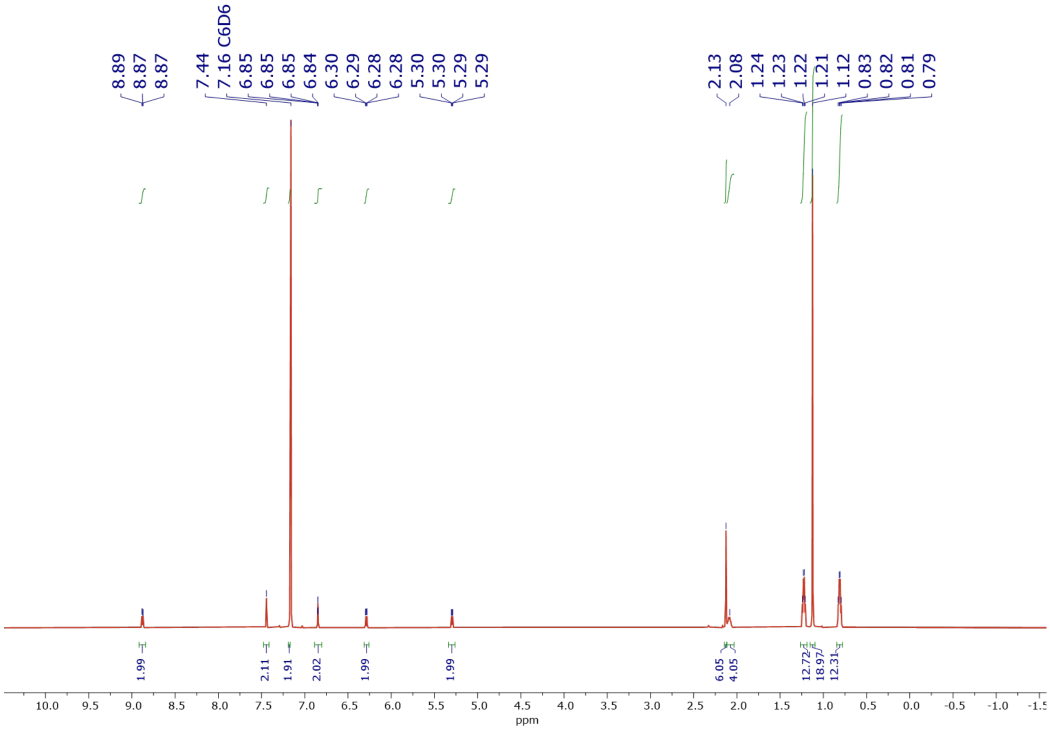


**Figure S6.** $^{1}H$ NMR spectrum (600 MHz) of **4** in benzene-$d_6$.

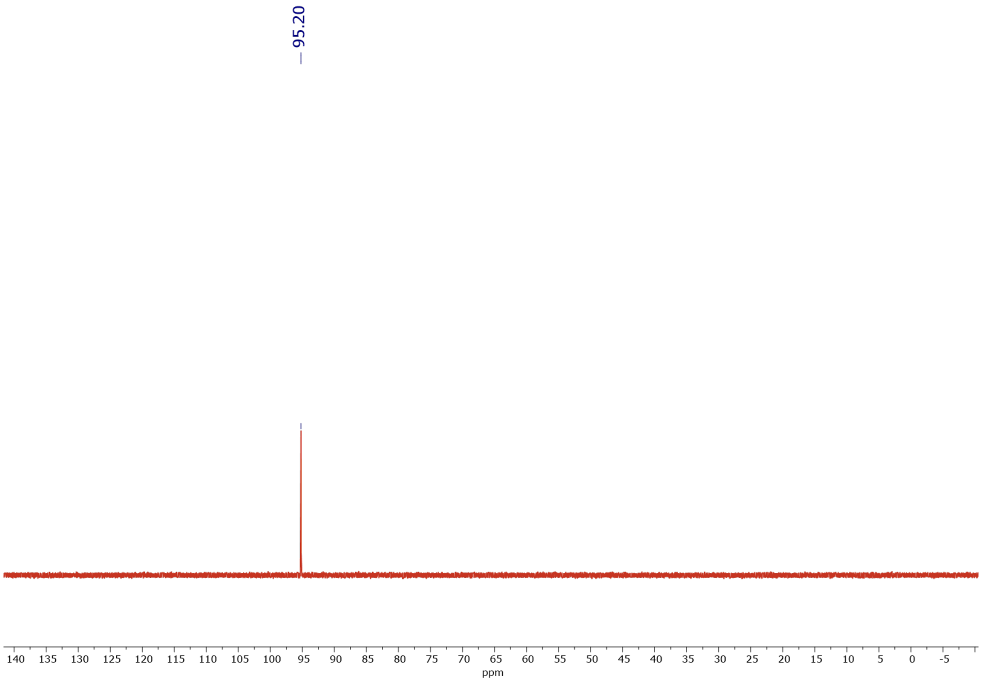


**Figure S7.** $^{31}P\{^{1}H\}$ NMR spectrum (243 MHz) of (**4**) in benzene-$d_6$.

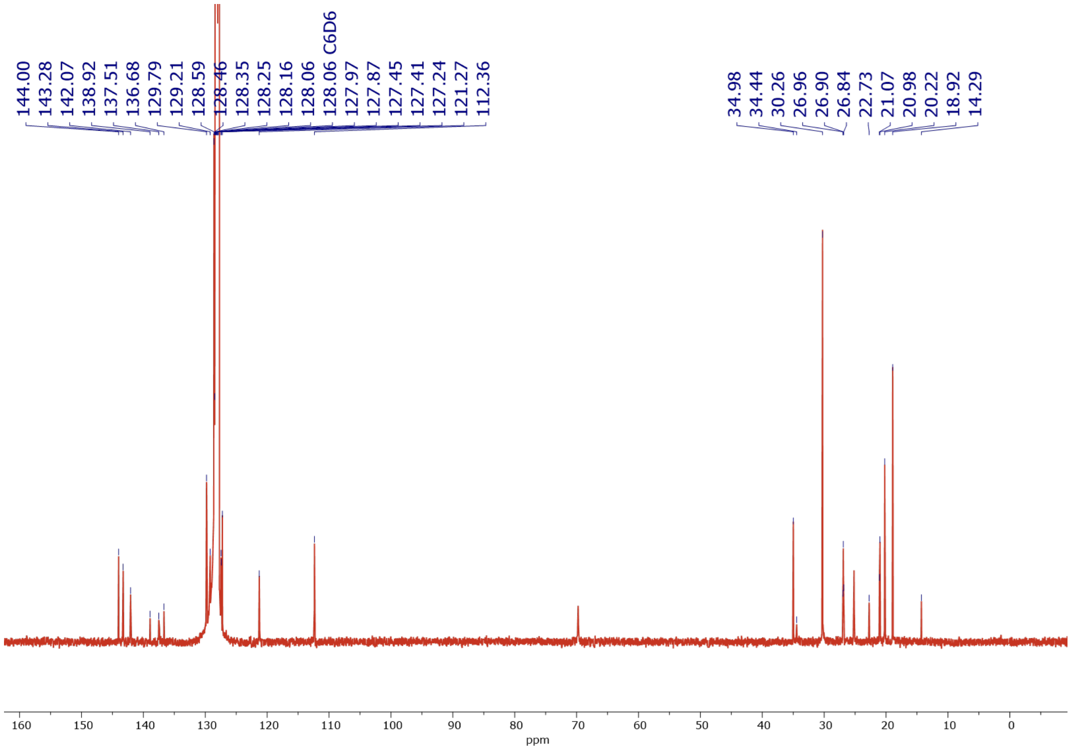


**Figure S8.** ${}^{13}C\{{}^{1}H\}$ NMR spectrum (126 MHz) of **4** in benzene-$d_6$.

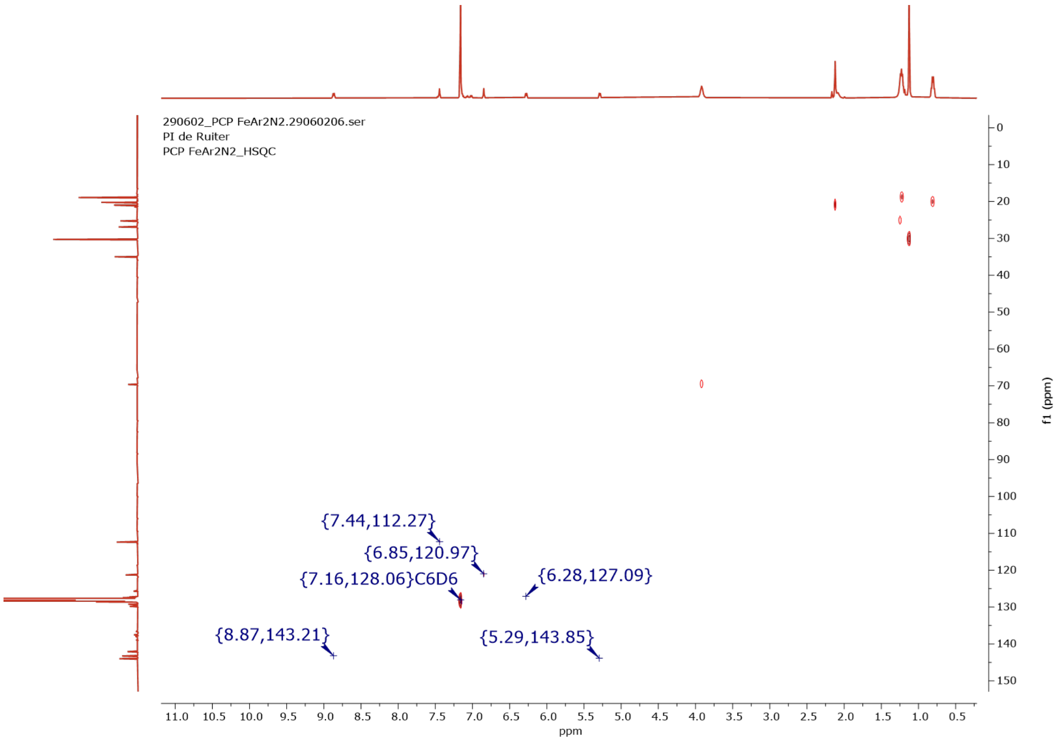


**Figure S9.** HSQC spectrum (500 MHz) of **4** in benzene-$d_6$.

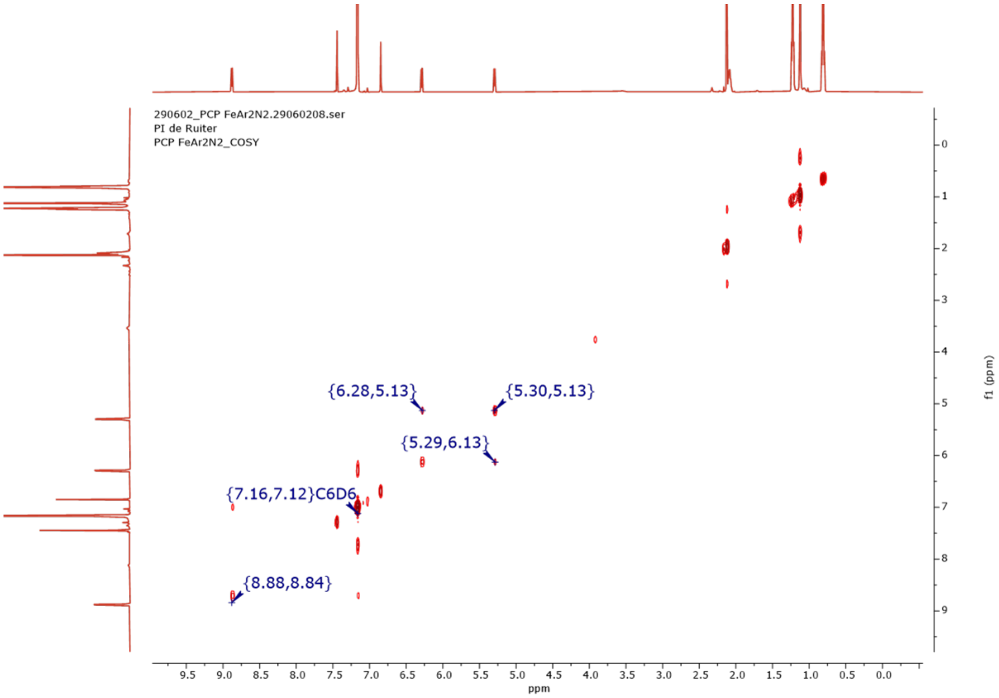


**Figure S10.** COSY spectrum (500 MHz) of **4** in benzene-$d_6$.

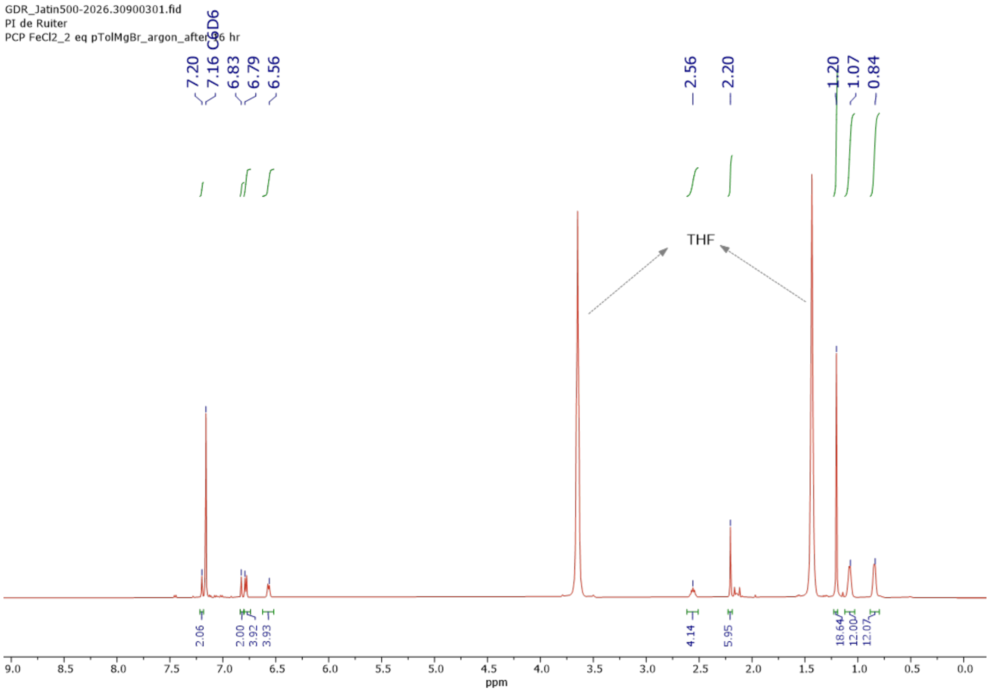


**Figure S11.** [1]H NMR spectrum (500 MHz) of **5** in benzene-$d_6$.

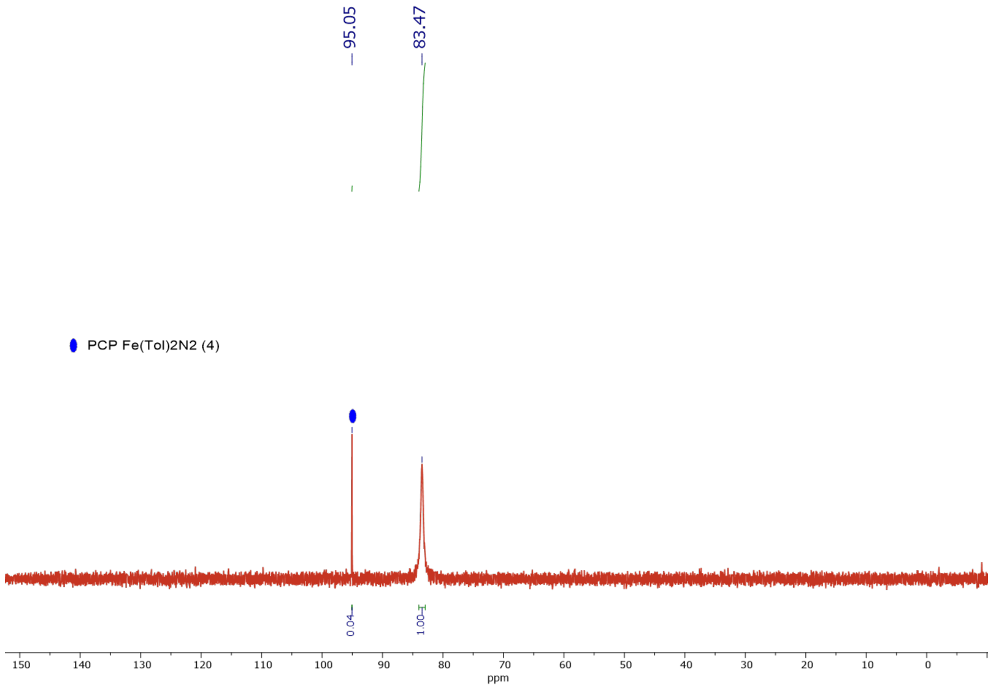


**Figure S12**. $^{31}P\{^{1}H\}$ NMR spectrum (202 MHz) of **5** in benzene-$d_6$.

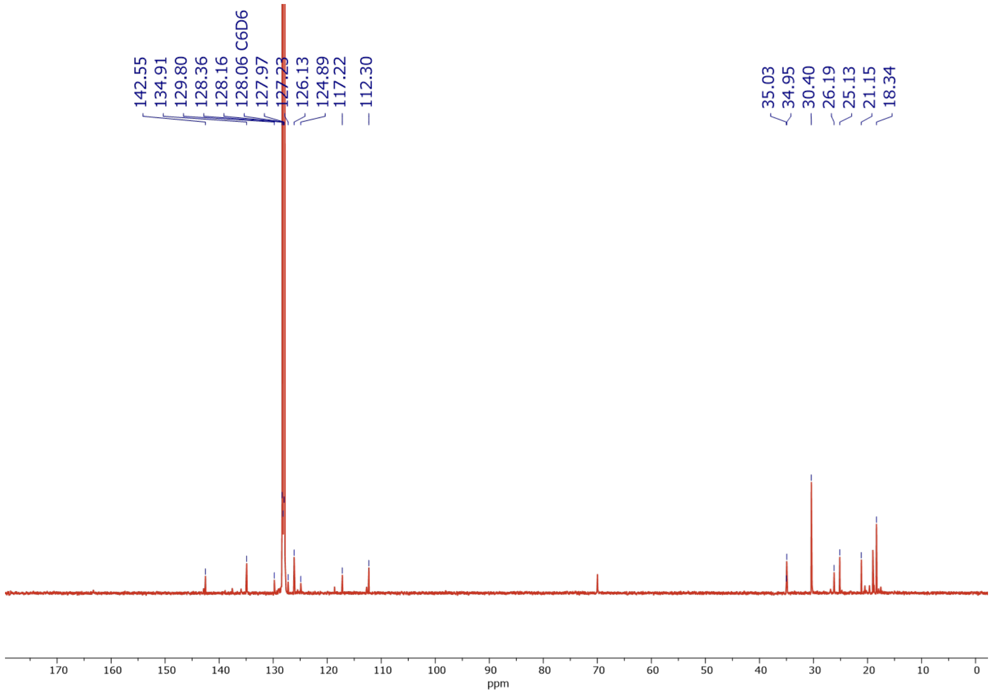


**Figure S13**. $^{13}C\{^{1}H\}$ NMR spectrum (126 MHz) of **5** in benzene-$d_6$.

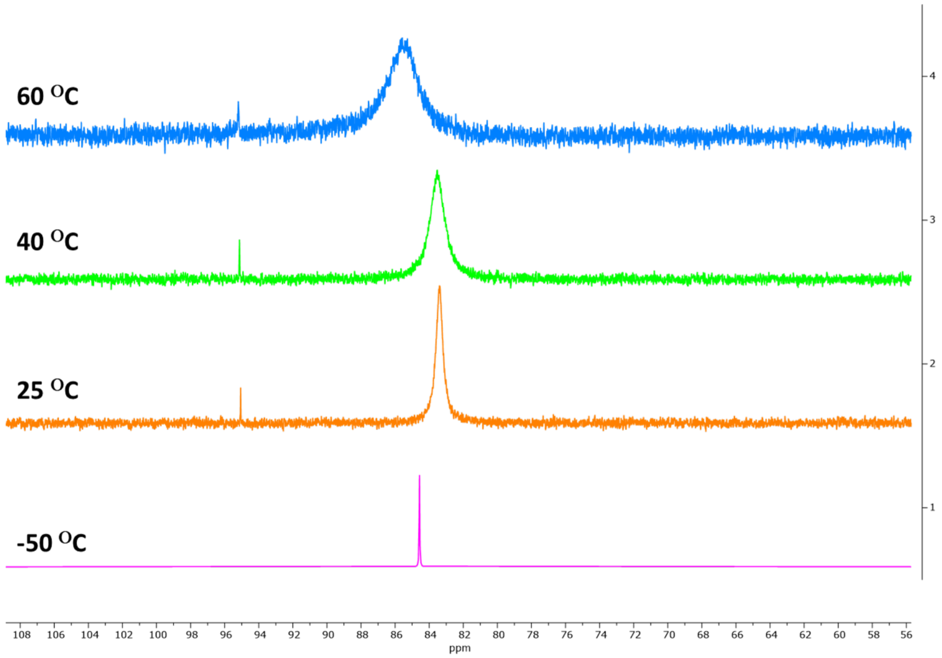


**Figure S14**. Variable temperature $^{31}P\{^{1}H\}$ NMR spectrum (202 MHz) of **5** in Toluene-$d_8$.

**Mössbauer Spectra**

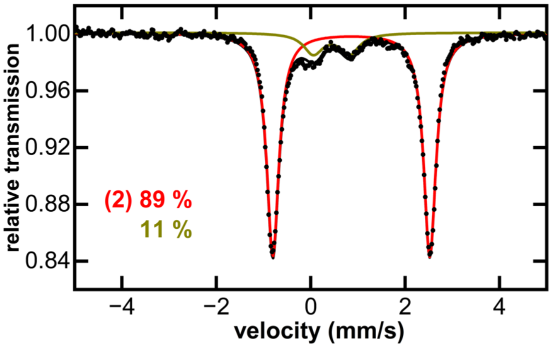


**Figure S15.** 80 K $^{57}$Fe Mössbauer spectra of frozen THF solution of *in-situ* generated [(PC$_{NHC}$)P)FeCl$_2$] (**2**) with Mössbauer parameters of $\delta$ = 0.86 mm/s $|\Delta E_Q|$ = 3.29 mm/s. The parameters for the species in dark yellow (impurity) are $\delta$ = 0.44 mm/s $|\Delta E_Q|$ = 0.74 mm/s respectively.

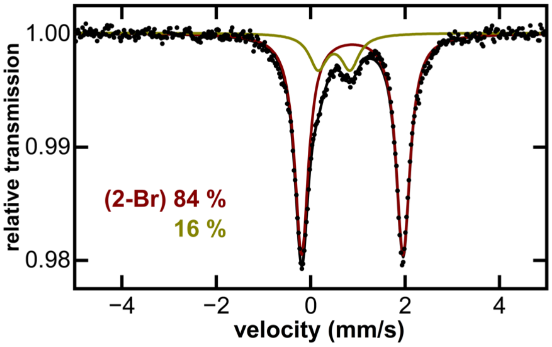


**Figure S16**. 80 K $^{57}$Fe Mössbauer of isolated crystals of [(PC$_{NHC}$)P)FeBr$_2$] (**2-Br**), with Mössbauer parameters of $\delta$ = 0.88 mm/s $|\Delta E_Q|$ = 2.15 mm/s.

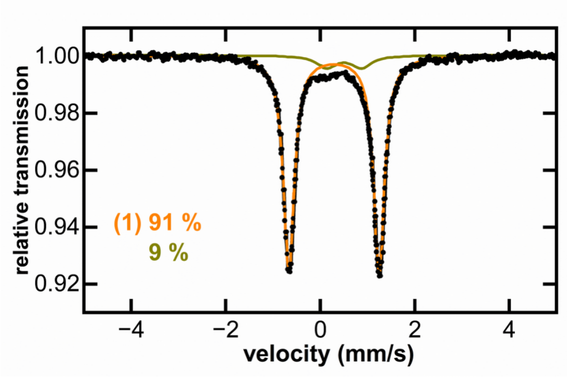


**Figure S17**. 80 K $^{57}$Fe Mössbauer spectra of frozen benzene solution of [(PC$_{NHC}$)P)Fe(N$_2$)$_2$] (**1**), with Mössbauer parameters of $\delta$ = 0.28 mm/s $|\Delta E_Q|$ = 2.10 mm/s. The parameters for the species in dark yellow (impurity) are $\delta$ = 0.44 mm/s $|\Delta E_Q|$ = 0.74 mm/s

### Reaction of Complex 2 with *p*TolMgBr in Toluene

In an argon-filled glovebox, a J-Young NMR tube was charged with [(PC$_{NHC}$P)FeCl$_2$] (**2**) (12 mg, 0.019 mmol), and Toluene-d$_8$ (0.4 mL) was added. To this suspension, was added 2.1 equivalents of pTolMgBr (0.040 mmol, 1.0 M solution in THF). The J. Young tube was sealed and heated at 45 °C for 1 h. After this period, the reaction mixture was analyzed by $^1$H and $^{31}$P NMR spectroscopy, confirming complete conversion to a diamagnetic PCP Fe(Tol)$_2$ (5).

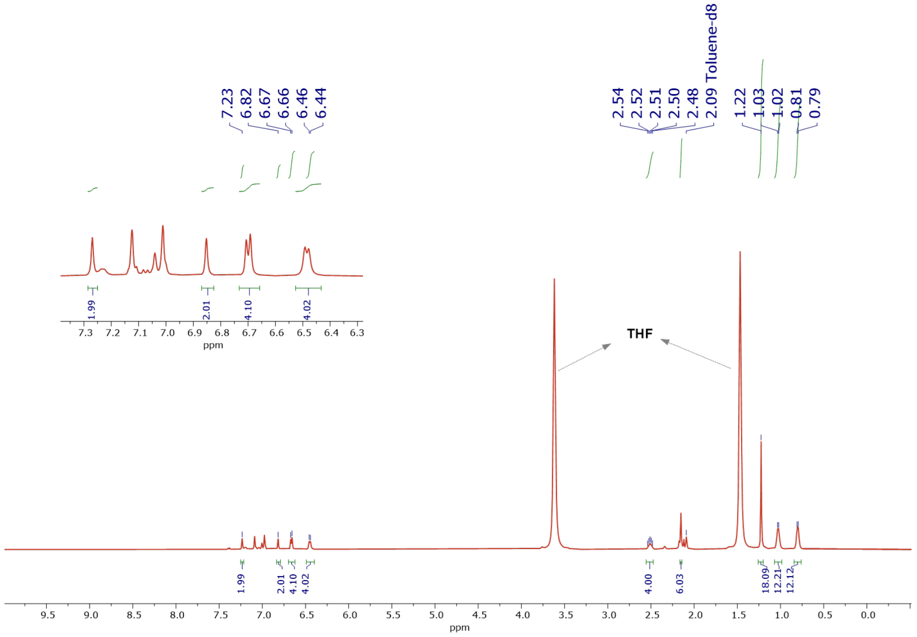


**Figure S18.** $^1$H NMR spectrum (500 MHz) of **5** in Toluene-$d_8$.

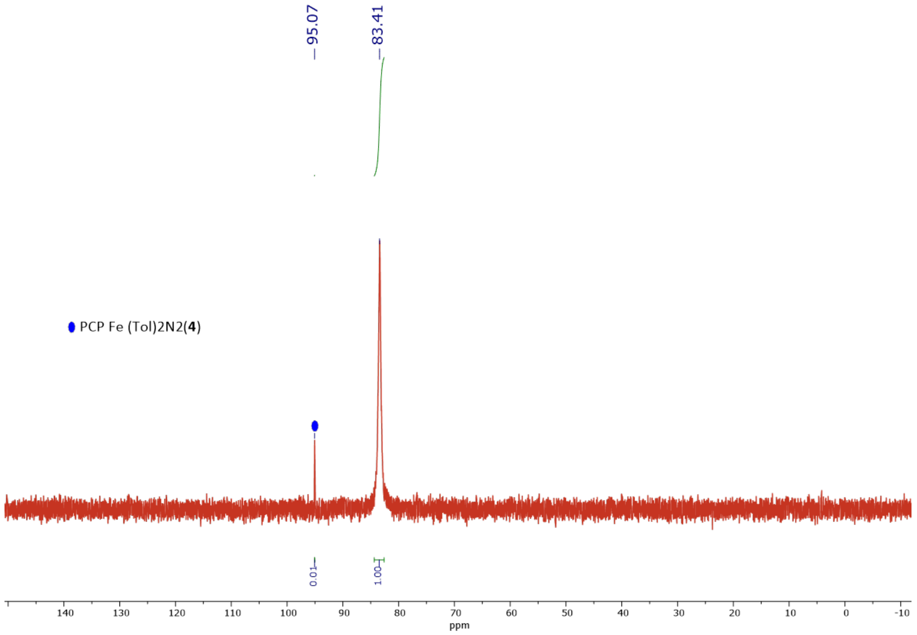


**Figure S19**. $^{31}$P{$^1$H} NMR spectrum (202 MHz) of **5** in Toluene-$d_8$.

**Solvent Dependence in the Reaction of Complex 2 with *p*TolMgBr**

In an argon-filled glovebox, a J-Young NMR tube was charged with [($PC_{NHC}P$)$FeCl_2$] (**2**) (12 mg, 0.019 mmol), and $C_6D_6$ (0.4 mL) was added. To this suspension was added 2.1 equivalents of pTolMgBr (0.040 mmol, 1.0 M solution in THF). The J. Young tube was sealed and heated at 45 °C for 1 h. After this period, the reaction mixture was analyzed by $^1$H NMR spectroscopy, confirming complete conversion to PCPFe(Tol)$_2$ (5). Following completion of the reaction, the solvent was removed under reduced pressure, and the residue was redissolved in $C_6D_6$ under an argon atmosphere for further NMR analysis. The resulting $^1$H NMR spectrum exhibited partially merged resonances in the aromatic region, indicative of dynamic behavior in solution. The solvent was again removed under vacuum, and the residue was subsequently dissolved in toluene-$d_8$ under an argon atmosphere. In contrast, the $^1$H NMR spectrum recorded in toluene-$d_8$ displayed sharp and well-resolved resonances. This solvent-dependent spectral behavior is consistent with weak intermolecular interactions in benzene, which are suppressed in toluene-$d_8$.

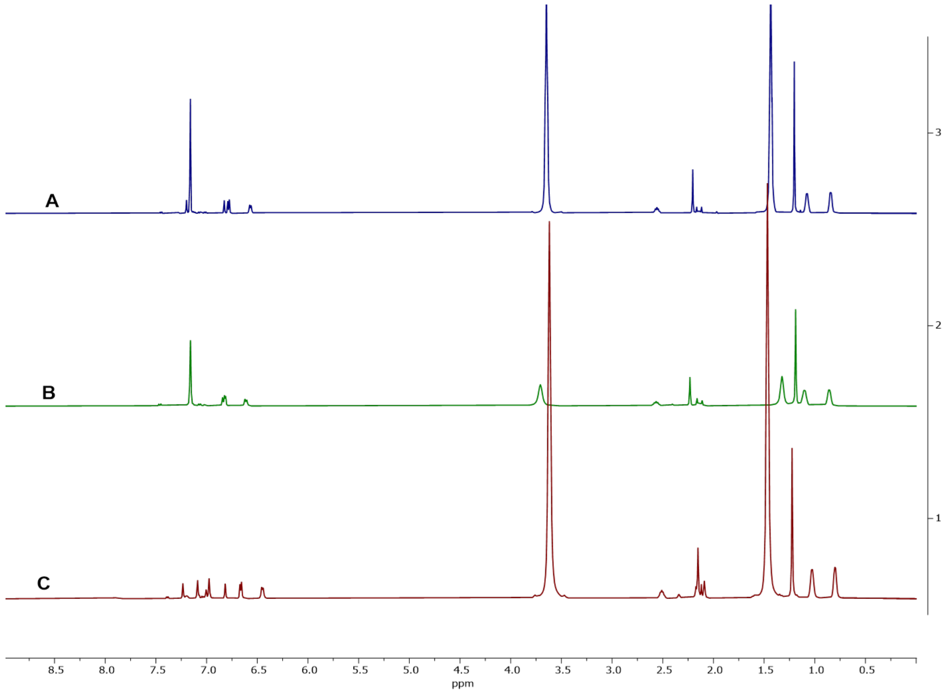


**Figure S20**. Stacked $^1$H NMR spectra of PCPFe(Tol)$_2$ (**5**) under an argon atmosphere: (A) in $C_6D_6$; (B) after solvent removal under vacuum and redissolution in fresh $C_6D_6$; and (C) thereafter solvent removal under vacuum and redissolution in toluene-$d_8$, illustrating solvent-dependent spectral behavior.

**Reaction of Complex 2 with 4-Fluorophenylmagnesium Bromide: Formation of a Low-Spin Fe (II) Symmetric Bis(aryl) Complex**

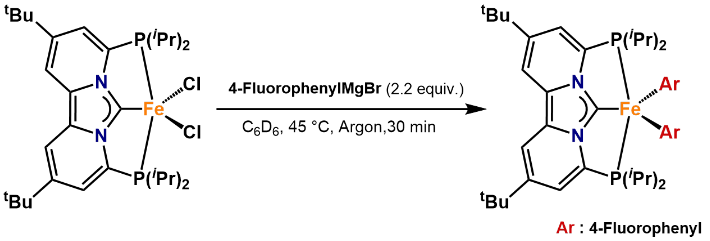


In an argon-filled glovebox, a J-Young NMR tube was charged with [(PC$_{NHC}$P)FeCl$_2$] (**2**) (12 mg, 0.019 mmol), and $C_6D_6$ (0.4 mL) was added. To this suspension was added 2.1 equivalents of 4-FluorophenylMgBr (0.040 mmol, 2.0 M solution in $Et_2O$). The J. Young tube was sealed and heated at 45 °C for 30 min. After this period, the reaction mixture was analyzed by $^{1}$H, $^{31}$P, $^{19}$F NMR spectroscopy. The $^{1}$H NMR spectrum indicates formation of a symmetric low-spin Fe(II) biaryl complex. In addition, the resulting $^{31}$P NMR exhibits a single resonance at δ = 79.52 ppm, while the $^{19}$F NMR spectrum shows a single signal at δ = −127.93 ppm, aside from minor resonances attributable to residual fluorobenzene present in the Grignard reagent. Given that paramagnetically shifted $^{19}$F signals are observed even for high-spin Fe(II) fluorinated aryl complexes, their absence here strongly supports the lack of any significant paramagnetic iron species in solution.

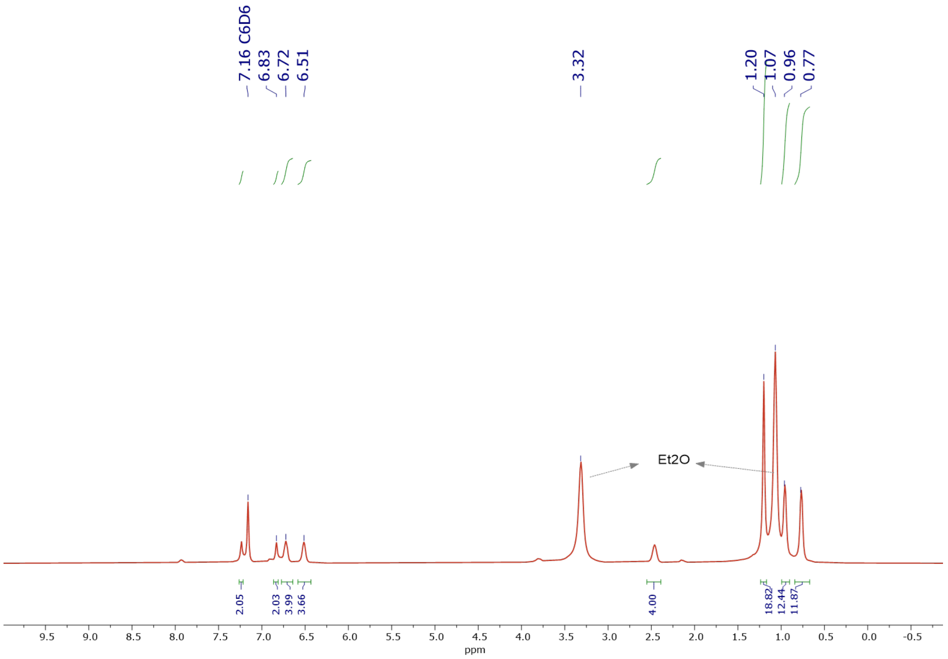


**Figure S21.** $^{1}$H NMR spectrum (500 MHz) of the crude reaction mixture upon addition of two equiv. of 4-FluorophenylMgBr to **2** under an atmosphere of argon in benzene-$d_6$

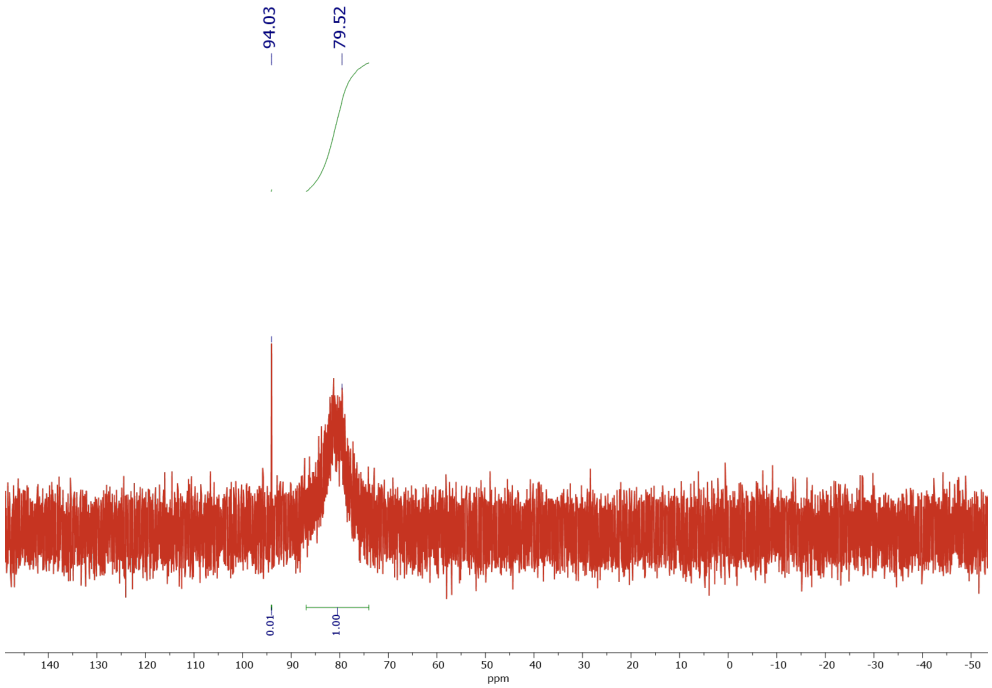


**Figure S22.** $^{31}P\{^{1}H\}$ NMR spectrum (202 MHz) of the crude reaction mixture upon addition of 2 equivalent of 4-FluorophenylMgBr to **2** under an atmosphere of argon in benzene-$d_6$

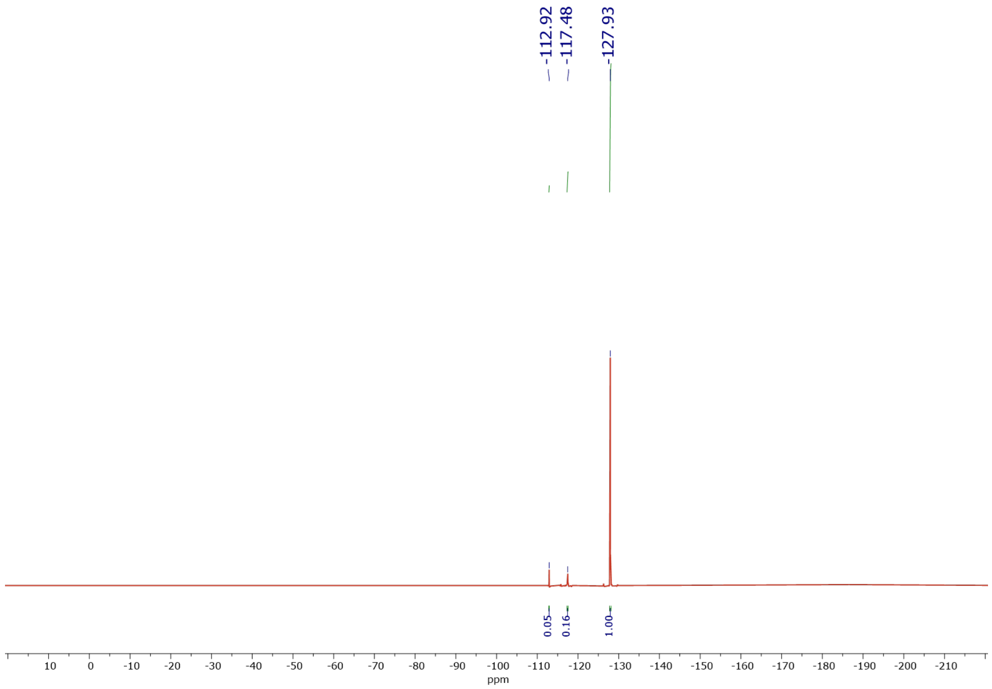


**Figure S23.** $^{19}F$ NMR spectrum ((471 MHz) of the crude reaction mixture upon addition of 2 equivalent of 4-FluorophenylMgBr to **2** under an atmosphere of argon in benzene-$d_6$

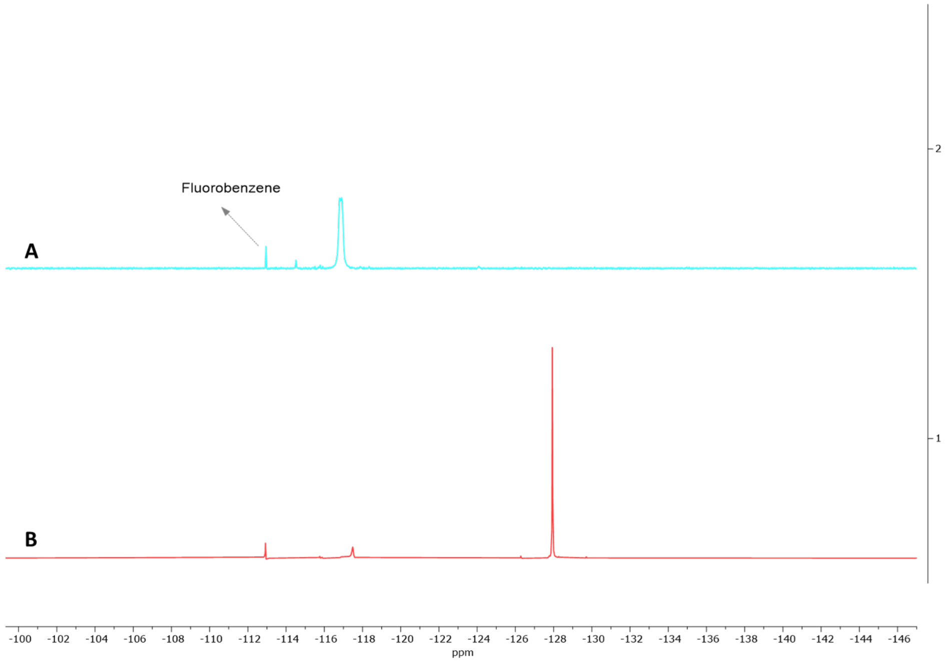


**Figure S24**. Stacked [19]F NMR of A) 4-FluorophenylMgBr (2M in Et2O), B) the reaction between ($PC_{NHC}P$)$FeCl_2$ (**2**) with 2 equivalent 4-FluorophenylMgBr in $C_6D_6$, indicating formation of diamagnetic complex (5) only.

**Reactivity of PCP Fe $(Tol)_2N_2$(4) with electrophile in $N_2$**

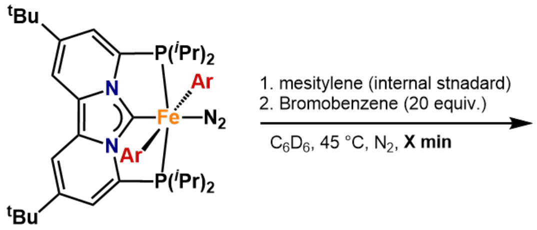


In an $N_2$-filled glovebox, a J-Young NMR tube was charged with [($PC_{NHC}P$)$FeTol_2N_2$] (**4**) (14 mg, 0.018 mmol), and $C_6D_6$ (0.4 mL) was added. To this solution was added 1 equivalent of mesitylene (0.018mmol) as internal standard and 20 equivalent of bromobenzene (0.36 mmol, 37 µL). The J. Young tube was sealed and heated at 45 °C for 2 h. After this period, the reaction mixture was analyzed by $^{1}H$, and $^{31}P$ NMR spectroscopy. The data indicates in nitrogen atmosphere, [($PC_{NHC}P$)$FeTol_2N_2$] (**4**) does not react with electrophile. We attribute this inhibition to coordination of the equatorial $N_2$ ligand in **4**, which both stabilizes the Fe(II) bis-aryl species and sterically/electronically blocks the approach required for productive aryl–aryl coupling.

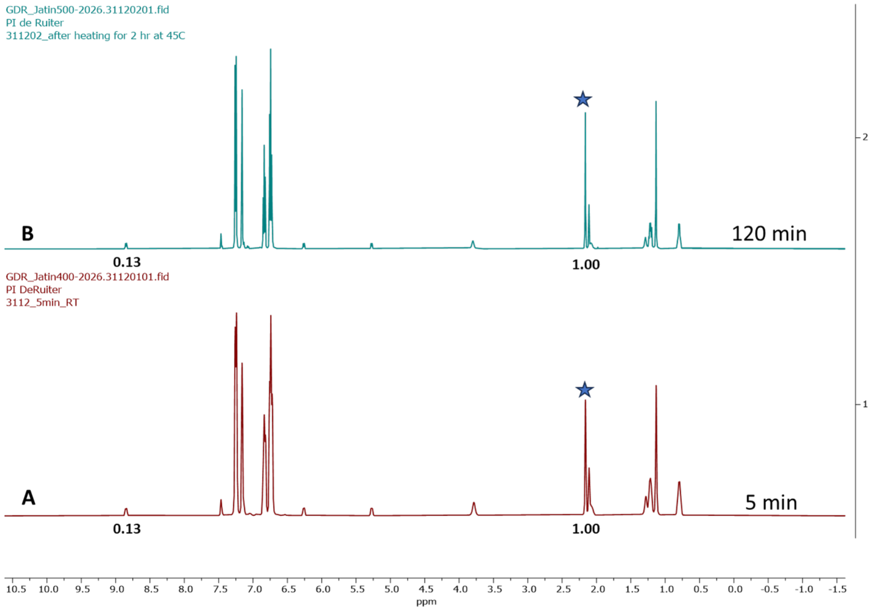


**Figure S25**. Stacked [1]H NMR of the reaction between [(PC$_{NHC}$P)FeTol$_2$N$_2$] (**4**) and bromobenzene (20 equivalent) in presence of mesitylene (as internal standard), under a nitrogen atmosphere after (A) 5 min and (B) after 2 hours, showing that no reaction happened. The peak marked with an asterisk corresponds to mesitylene (internal standard) and the other assigned peak correspond to (*o*-CH of **C$_6$H$_4$** CH$_3$) of complex **4**.

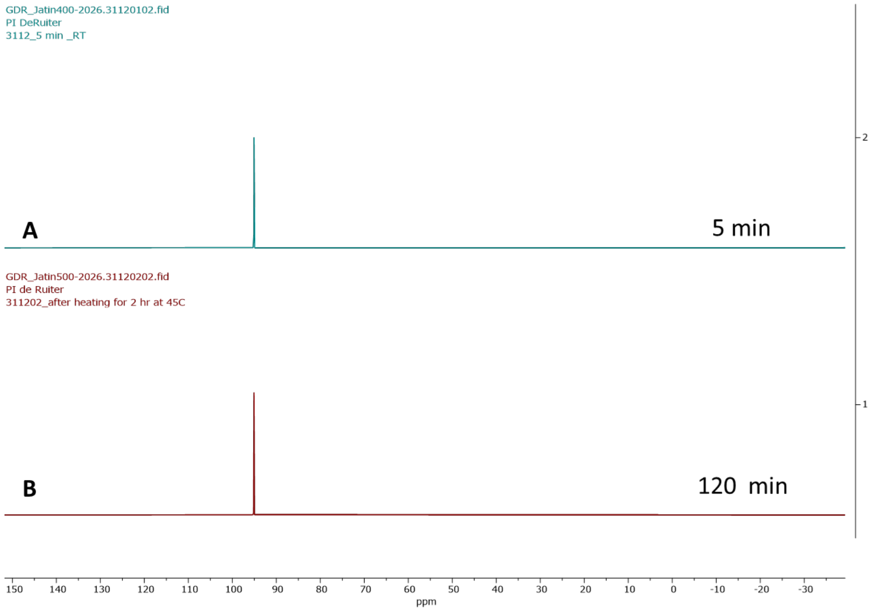


**Figure S26**. Stacked [31]P NMR of the reaction between [(PC$_{NHC}$P)FeTol$_2$N$_2$] (**4**) and bromobenzene (20 equivalent) in presence of mesitylene (as internal standard), under a nitrogen atmosphere after (A) 5 min and (B) after 2 hours, showing that no reaction happened.

## Reactivity of PCP Fe $(Tol)_2N_2$(4) with electrophile in argon

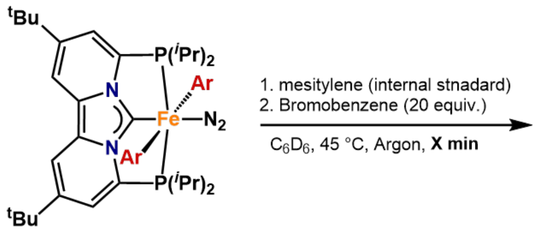


In an argon filled glovebox, a J-Young NMR tube was charged with [$(PC_{NHC}P)FeTol_2N_2$] (**4**) (14 mg, 0.018 mmol), and $C_6D_6$ (0.4 mL) was added. To this solution was added 1 equivalent of mesitylene (0.018mmol) as internal standard and 20 equivalent of bromobenzene (0.36 mmol, 37 µL). The J. Young tube was sealed and heated at 45 °C for 2 h. After this period, the reaction mixture was analyzed by $^1$H, and $^{31}$P NMR spectroscopy. These data indicate, complex 4 remains unchanged with 10% conversion to PCP FeTolCl (**3**).

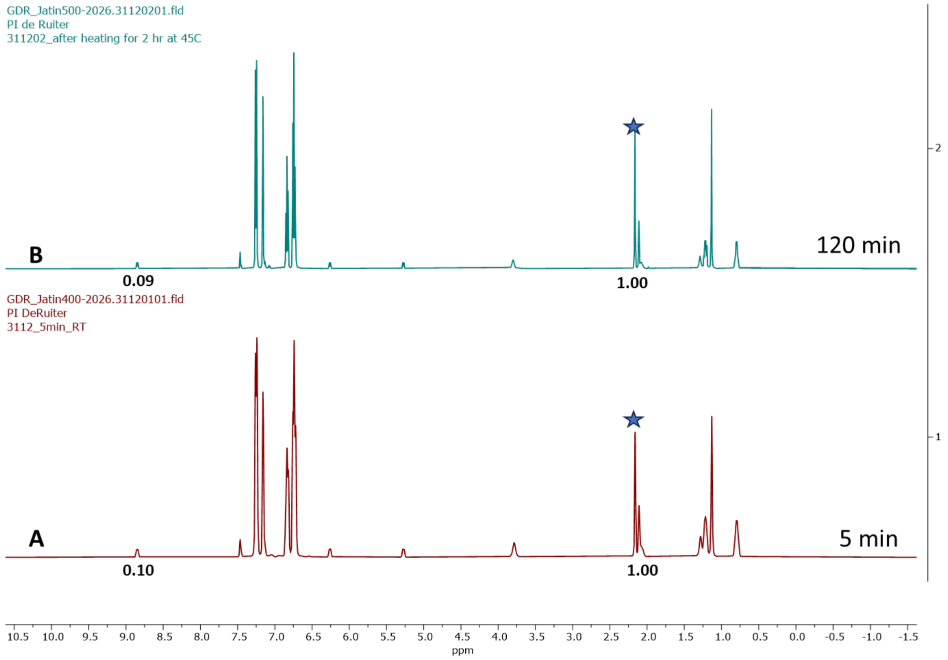


**Figure S27**. Stacked $^1$H NMR of the reaction between [$(PC_{NHC}P)FeTol_2N_2$] (**4**) and bromobenzene (20 equivalent) in presence of mesitylene (as internal standard), under a argon atmosphere after 5 min (A) and after 2 hours (B), showing that no reaction happened. The peak marked with an asterisk corresponds to mesitylene (internal standard) and the other assigned peak correspond to (*o*-CH of $\mathbf{C_6H_4}$ $CH_3$) of complex **4**.

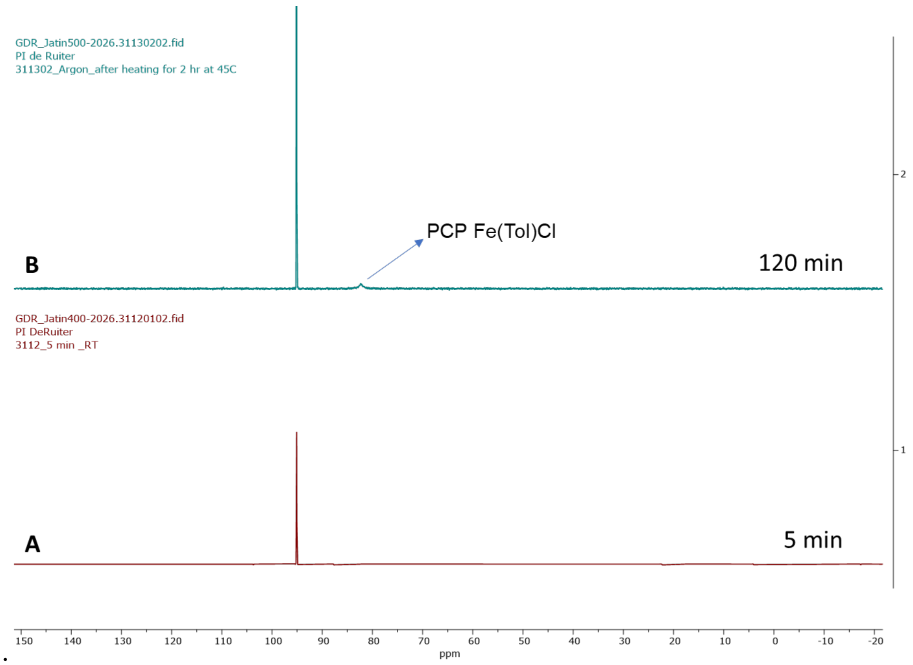


**Figure S28**. Stacked $^{31}$P NMR of the reaction between [(PC$_{NHC}$P)FeTol$_2$N$_2$] (**4**) and bromobenzene (20 equivalent) in presence of mesitylene (as internal standard), under a argon atmosphere after 5 min (A) and after 2 hours (B), showing the non-reactivity of complex **4** with electrophile.

## Radical Trapping Experiments

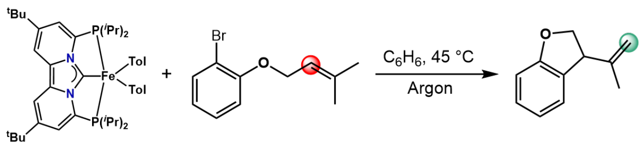


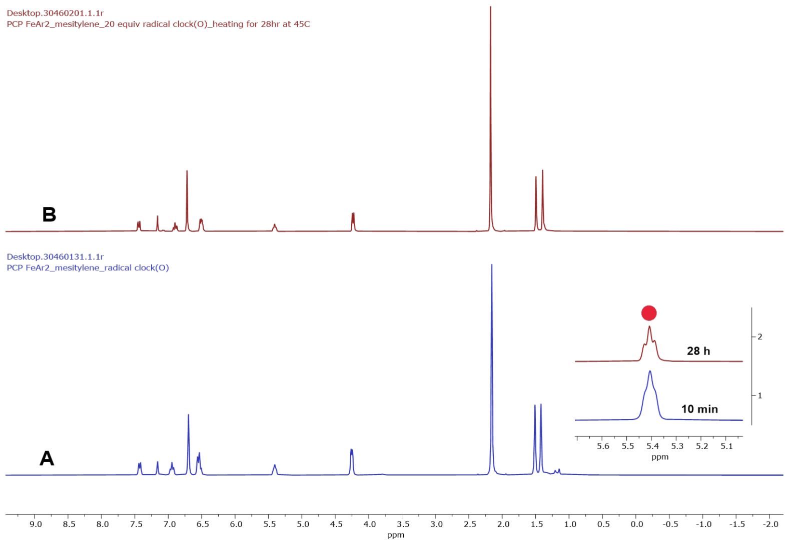


**Figure S29**. Stacked $^1$H NMR of the reaction between [(PC$_{NHC}$P)Fe(Tol)$_2$] (**5**) and 1-bromo-2-((3-methylbut-2-en-1-yl)oxy) benzene under an argon atmosphere after 10 min (A) and after 28 hours (B), showing that no reaction happened.

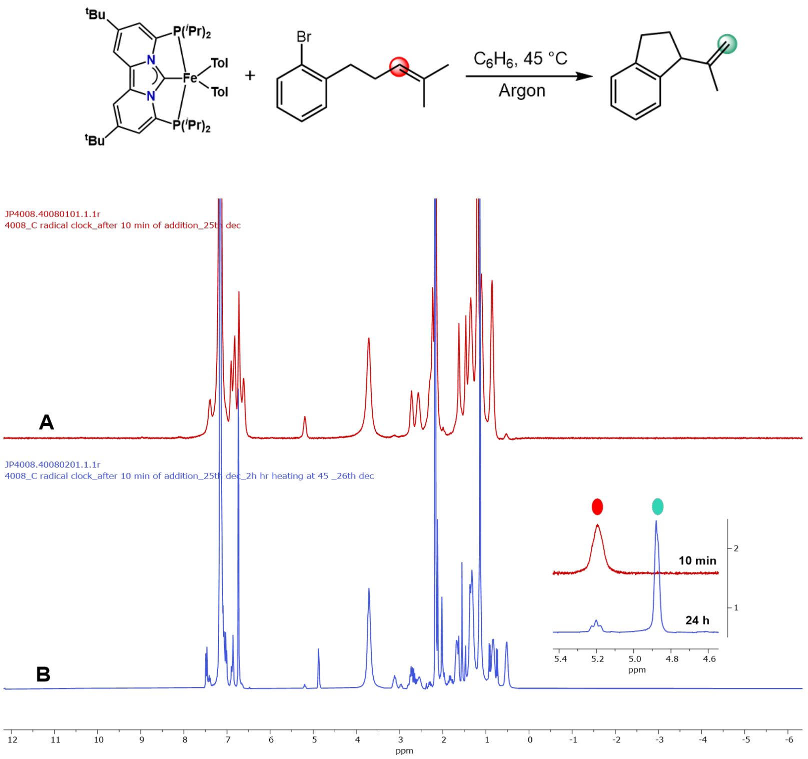


**Figure S30**. Stacked [1]H NMR of the reaction between [(PC$_{NHC}$P)Fe(Tol)$_2$] (**5**) and 1-bromo-2-(4-methylpent-3-en-1-yl)benzene under an argon atmosphere after 10 min (A) and after 28 hours (B), showing evidence for a homolytic radical bond cleavage from **5**.

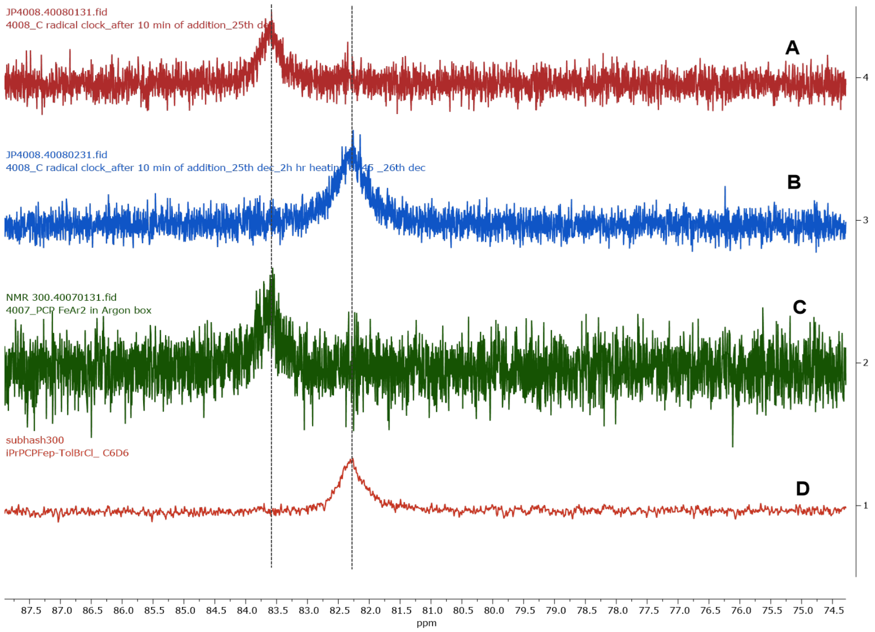


**Figure S31.** Stacked [31]P NMR of the reaction between of the reaction between [(PC$_{NHC}$P)Fe(Tol)$_2$] (**5**) and -bromo-2-(4-methylpent-3-en-1-yl)benzene in argon atmosphere after 10 min (A) and after 28 hours (B). (C and D) [31]P NMR spectra of complexes **5** and **3** showing conversion of **5** to 3 in spectra A and B.

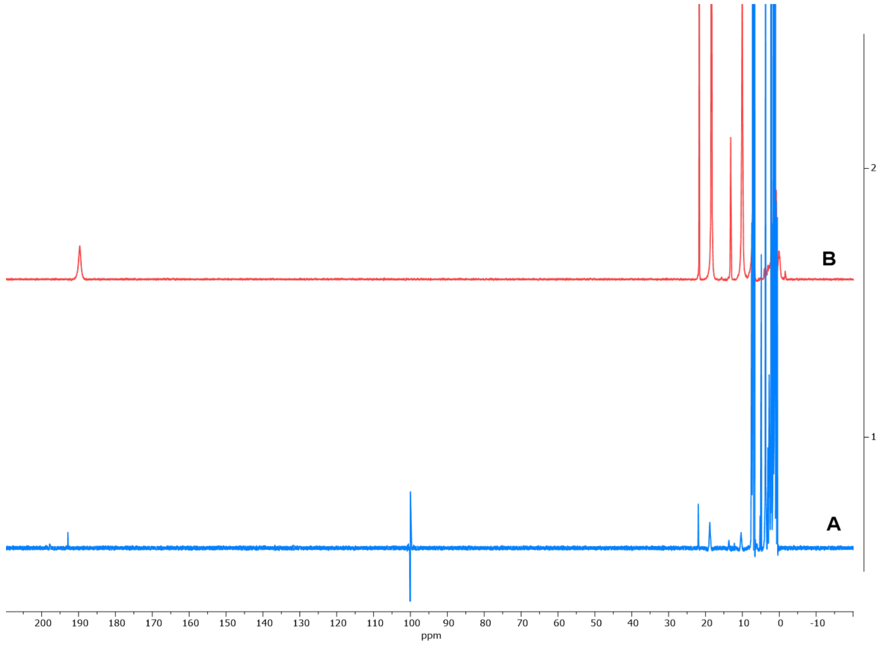


**Figure S32**. Stacked [1]H NMR spectrum (300 MHz) of the crude reaction mixture of the reaction between $[(PC_{NHC}P)Fe(Tol)_2]$ (**5**) and 1-bromo-2-(4-methylpent-3-en-1-yl)benzene under an argon (bottom) and that of $[(PC_{NHC}P)FeBr_2]$ (top) , showing presence of $[(PC_{NHC}P)FeBr_2]$ (**1**) in the crude reaction mixture.

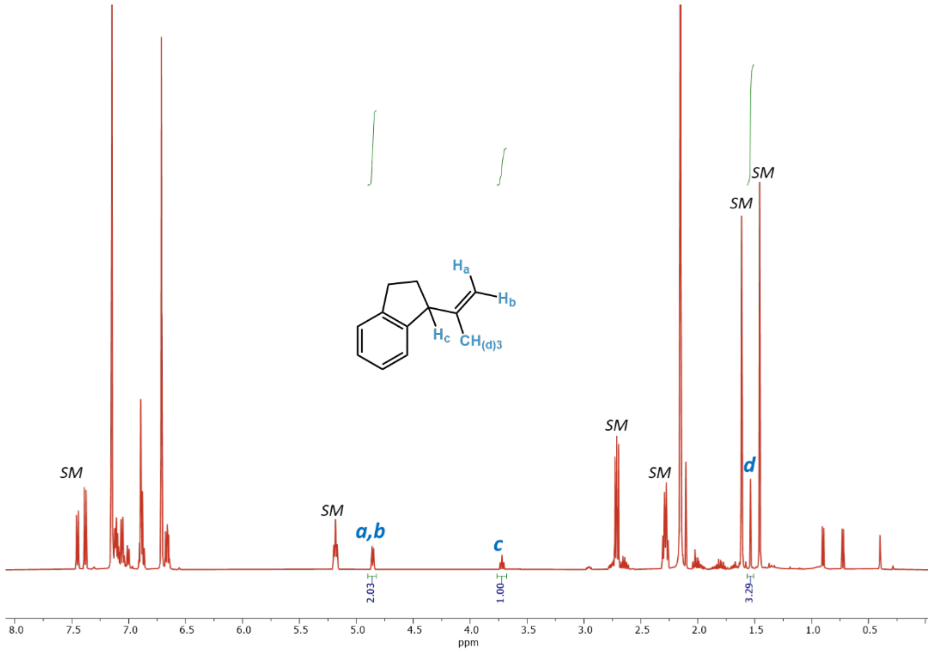


**Figure S33**. [1]H NMR of the organic fraction of the obtained from the reaction between $[(PC_{NHC}P)Fe(Tol)_2]$ (**5**) and 1-bromo-2-(4-methylpent-3-en-1-yl)benzene under an argon atmosphere. SM represents unreacted 1-bromo-2-(4-methylpent-3-en-1-yl) benzene.

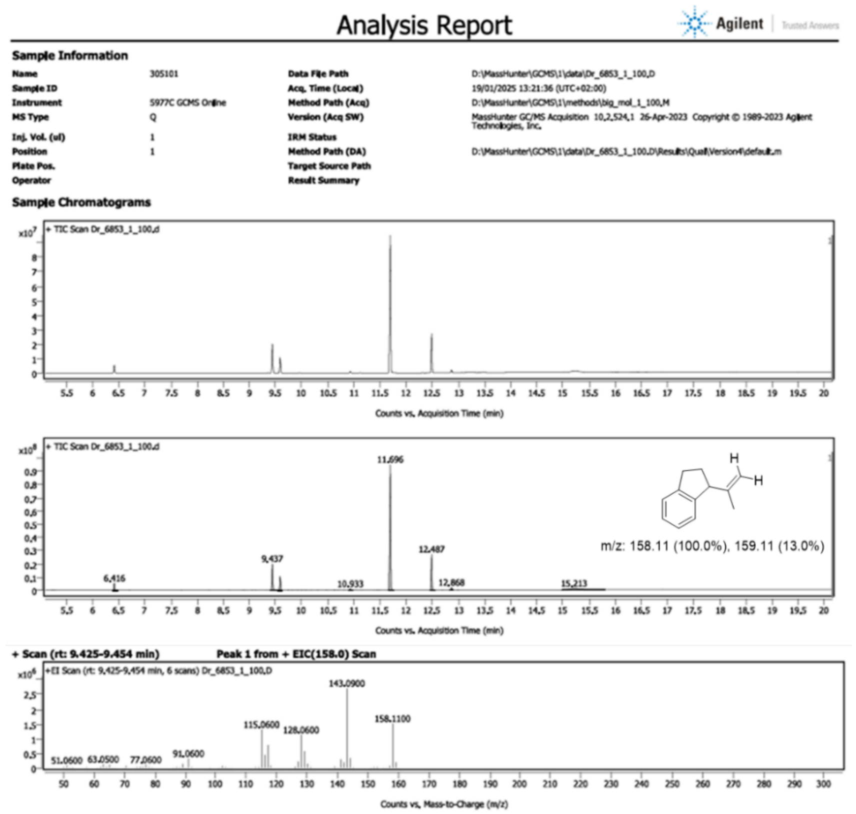


**Figure S34**. GS-trace and mass spectra of the organic fraction of the obtained from the reaction between [(PC$_{NHC}$P)Fe(Tol)$_2$] (**5**) and 1-bromo-2-(4-methylpent-3-en-1-yl)benzene under an argon atmosphere, showing the formation of the cyclized product.

**The Reaction of Complex 2 with Nucleophile (*p*TolMgBr or PhLi)**

**General Procedure for the reaction of [(PC$_{NHC}$P)FeCl$_2$] (2) with nucleophile (*p*TolMgBr)**. In an Ar-filled glovebox, to a stirred solution of [(PC$_{NHC}$P)FeCl$_2$] (0.042 mmol, 7.5 mg of $^{57}$Fe labeled complex, and 19.5 mg of the unlabeled complex) in benzene (1 mL) in a 4 mL scintillation vial, was added, 1, 2, 3, or 40 equivalents of a suspension of Grignard reagent (0.042 mmols, 32 μL, from 1.33 mM stock solution in benzene. The stock solution of the Grignard reagent was prepared by removing the THF under vacuum and dissolving the resulting residue in benzene which upon titration, give a final concentration of 1.33 mM.) or solid PhLi (2 equiv. 0.084 mmol, 8 mg), at 45 °C. After 5 minutes, an aliquot of approximately 0.5 mL was transferred into a Delrin Mössbauer cup and immediately freeze-trapped by placing the cup into a cold well containing condensed argon generated by cooling argon gas with liquid nitrogen. Samples collected at later time points, as well as those prepared with different equivalents of *p*TolMgBr (2, 3, and 40 equiv), were handled in the same manner. For experiments conducted under a nitrogen atmosphere, the Mössbauer samples were freeze-trapped by direct immersion of the Delrin cup into liquid nitrogen. For comparison a table containing the Mössbauer parameters for the iron species identified in these studies is given in Table S1.

**Table S1**. Summary of 80 K $^{57}$Fe Mössbauer parameters for PC$_{NHC}$P-Fe complexes identified in this work. Frozen benzene solution and solid samples.

| Complex | Sample | Geometry | δ (mm/s) | \|ΔE$_Q$\| (mm/s) |
|---|---|---|---|---|
| PCPFeCl$_2$ (**1**) | solid | TBP | 0.87 | 3.44 |
| | frozen solution | TBP | 0.86 | 3.29 |
| PCPFeBr$_2$ (**1-Br**) | solid | TBP | 0.88 | 2.15 |
| PCPFe(N$_2$)$_2$ (**2**) | solid | TBP | 0.30 | 1.97 |
| | frozen solution | TBP | 0.28 | 2.10 |
| PCPFeTolCl (**3**) | solid | TBP | 0.20 | 1.19 |
| | frozen solution | TBP | 0.21 | 1.23 |
| PCPFeTol$_2$N$_2$ (**4**) | solid | OH | 0.12 | 1.60 |
| | frozen solution | OH | 0.11 | 1.61 |
| PCPFeTol$_2$ (**5a**) | frozen solution | | 0.07 | 1.80 |
| PCPFeTol$_2$ (**5b**) | frozen solution | | 0.12 | 2.31 |

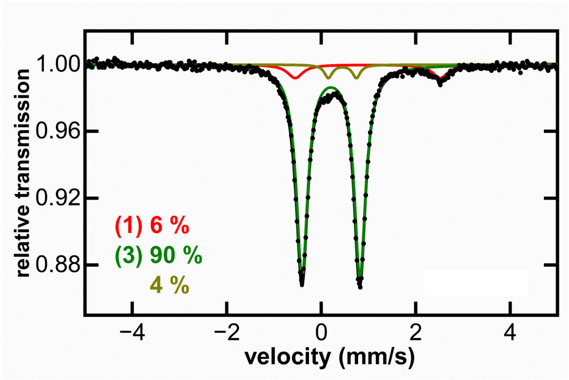


**Figure S35**. 80 K [57]Fe Mössbauer spectra of frozen benzene solution of [(PC$_{NHC}$P)Fe(Tol)(Cl)] (**3**), generated *in-situ* upon addition of one equiv. of *p*TolMgBr to a 42mM solution of **2**, with Mössbauer parameters of $\delta$ = 0.21 mm/s $|\Delta E_Q|$ = 1.23 mm/s. The parameters for the species in dark yellow (impurity) are $\delta$ = 0.44 mm/s $|\Delta E_Q|$ = 0.74 mm/s.

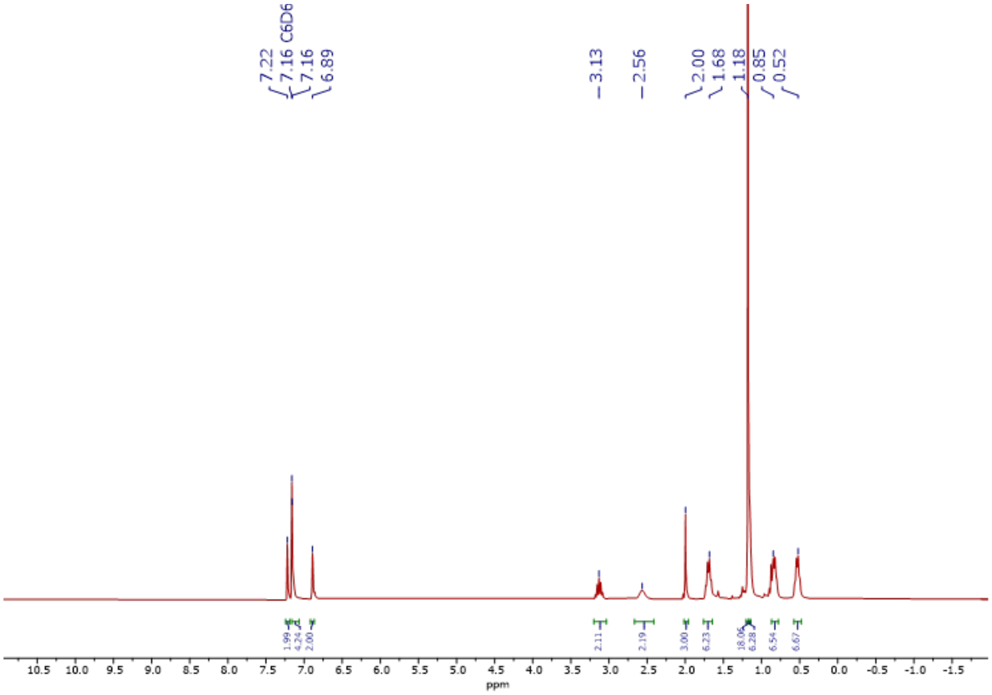


**Figure S36**.[1]H NMR spectra of *in-sit*u generated [(PC$_{NHC}$P)Fe(Tol)(Cl)] (**3**).

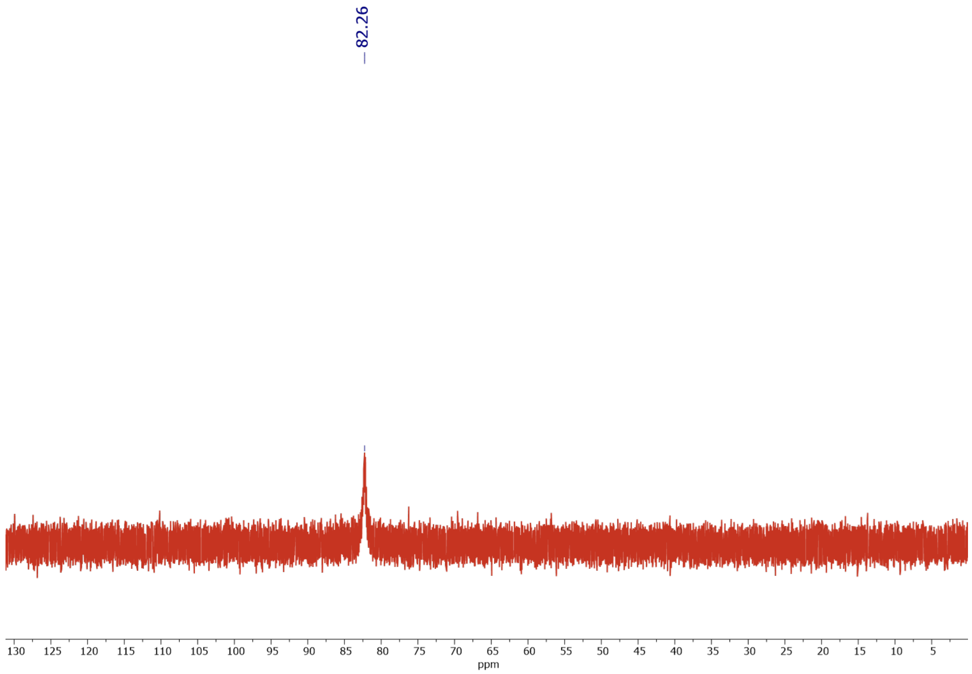


**Figure S37**. $^{31}$P NMR spectra of *in-sit*u generated [(PC$_{NHC}$P)Fe(Tol)(Cl)] (**3**).

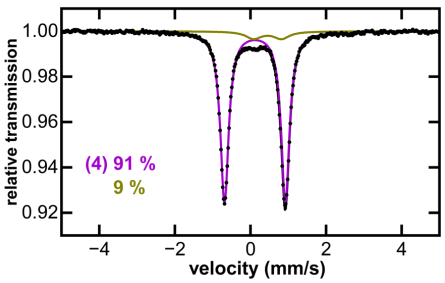


**Figure S38**. 80 K $^{57}$Fe Mössbauer spectra of frozen benzene solution of [($PC_{NHC}P$)Fe(Tol)$_2$($N_2$)] (**4**), generated *in-situ* upon addition of two equiv. of *p*TolMgBr to a 42mM solution of **2** under an atmosphere of $N_2$, with Mössbauer parameters of $\delta$ = 0.11 mm/s $|\Delta E_Q|$ = 1.61 mm/s. The parameters for the species in dark yellow (impurity) are $\delta$ = 0.44 mm/s $|\Delta E_Q|$ = 0.74 mm/s, respectively

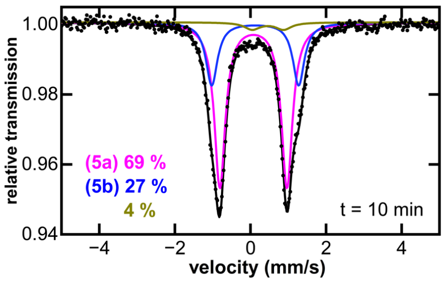


**Figure S39**. 80 K $^{57}$Fe Mössbauer spectra of frozen benzene solution of [($PC_{NHC}P$)Fe(Tol)$_2$] (**5a/5b**), generated *in-situ* upon addition of two equiv. of *p*TolMgBr to a 42mM solution of **2** under an atmosphere of Ar, with Mössbauer parameters magenta component (**5a**) $\delta$ = 0.07 mm/s $|\Delta E_Q|$ = 1.80 mm/s; and blue component (**5b**) $\delta$ = 0.12 mm/s $|\Delta E_Q|$ = 2.31 mm/s . The parameters for the species in dark yellow (impurity) are $\delta$ = 0.44 mm/s $|\Delta E_Q|$ = 0.74 mm/s.

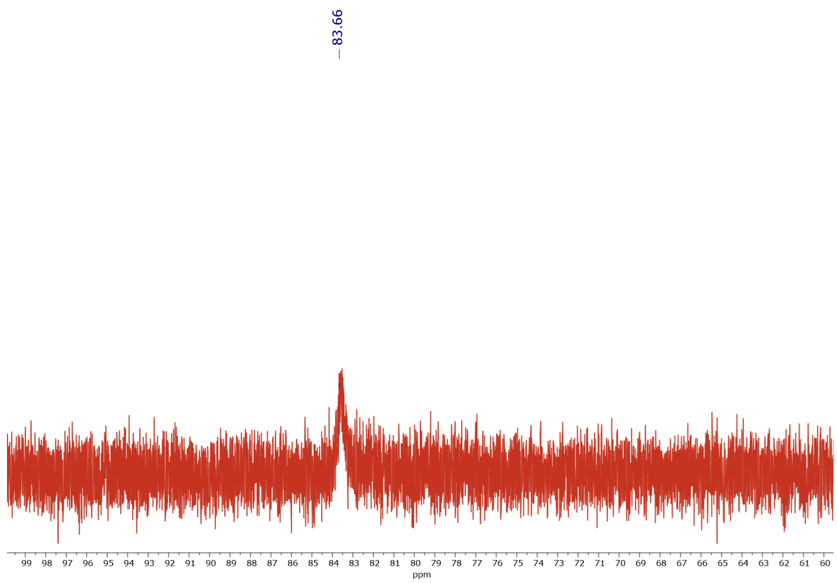


**Figure S40**. $^{31}$P NMR spectra of *in-sit*u generated [(PC$_{NHC}$P)Fe(Tol)$_2$] (**5a/5b**).

**Reaction of [(PC$_{NHC}$P)FeCl$_2$] (2) with nucleophile (*p*TolMgBr) in THF**.

In an Ar-filled glovebox, to a stirred solution of [(PC$_{NHC}$P)FeCl$_2$] (0.042 mmol, 7.5 mg of $^{57}$Fe labeled complex, and 19.5 mg of the unlabeled complex) in THF (1 mL) in a 4 mL scintillation vial, was added, 2 equivalents of a suspension of Grignard reagent (0.084 mmols, 84 µL, from 1 mM solution in THF) at 45 °C. After 30 minutes, an aliquot of approximately 0.5 mL was transferred into a Delrin Mössbauer cup and immediately freeze-trapped by placing the cup into a cold well containing condensed argon generated by cooling argon gas with liquid nitrogen.

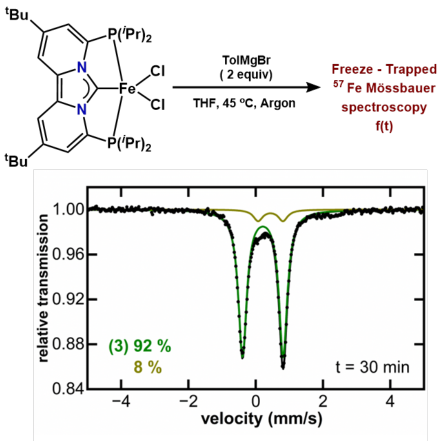


**Figure S41**. Freeze-trapped 80 K $^{57}$Fe Mössbauer spectra of reaction of **2** with two equiv. of *p*TolMgBr in THF under an argon atmosphere, suggesting 2$^{nd}$ transmetalation not happening in THF. Mössbauer parameters of $\delta$ = 0.21 mm/s $|\Delta E_Q|$ = 1.23 mm/s are equivalent to those observed for compound **3**. The parameters for the species in dark yellow (impurity) are $\delta$ = 0.44 mm/s $|\Delta E_Q|$ = 0.74 mm/s.

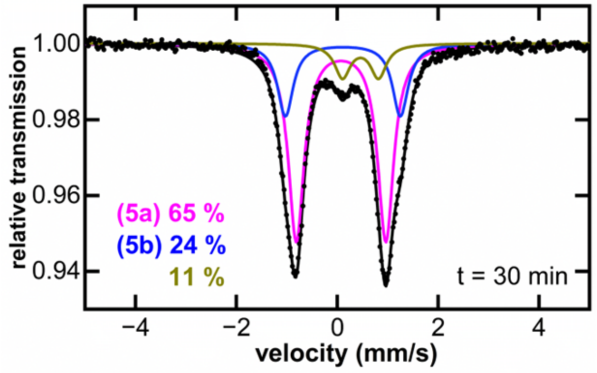


**Figure S42**. Freeze-trapped 80 K $^{57}Fe$ Mössbauer spectra of reaction of **2** with two equivalents of solid phenyl lithium (PhLi) in benzene under argon atmosphere. Mössbauer parameters green component magenta component $\delta = 0.07$ mm/s $|\Delta E_Q| = 1.77$ mm/s (**5a**) and blue component $\delta = 0.12$ mm/s $|\Delta E_Q| = 2.28$ mm/s (**5b**).

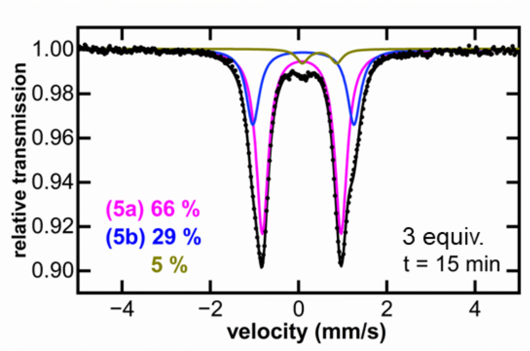


**Figure S43.** Freeze-trapped 80 K $^{57}Fe$ Mössbauer spectra of the crude reaction mixture upon addition of three equiv. of *p*TolMgBr to a 42mM solution of **2** under an atmosphere of Ar. Mössbauer parameters for magenta component δ = 0.07 mm/s $|\Delta E_Q|$ = 1.80 mm/s (**5a**), blue component δ = 0.12 mm/s $|\Delta E_Q|$ = 2.31 mm/s (**5b**) and dark yellow (impurity) δ = 0.44 mm/s $|\Delta E_Q|$ = 0.74 mm/s, showing there is no formation of any iron ate complexes.

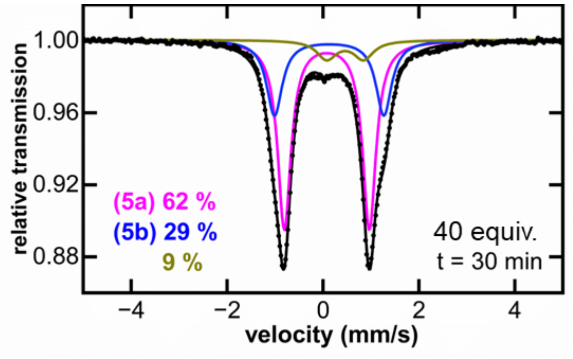


**Figure S44.** Freeze-trapped 80 K $^{57}Fe$ Mössbauer spectra of the crude reaction mixture upon addition of forty equiv. of *p*TolMgBr to a 42mM solution of **2** under an atmosphere of Ar. Mössbauer parameters for magenta component δ = 0.07 mm/s $|\Delta E_Q|$ = 1.80 mm/s (**5a**), blue component δ = 0.12 mm/s $|\Delta E_Q|$ = 2.31 mm/s (**5b**) and dark yellow (impurity) δ = 0.44 mm/s $|\Delta E_Q|$ = 0.74 mm/s, showing there is no formation of any iron ate complexes.

**Reaction of [(PC$_{NHC}$P)FeCl$_2$] (2) with nucleophile (*p*TolMgBr) under argon and nitrogen:**

In an Ar-filled glovebox, to a stirred solution of [(PC$_{NHC}$P)FeCl$_2$] (0.126 mmol, 22.5 mg of $^{57}$Fe labeled complex, and 58.5 mg of the unlabeled complex) in benzene (3 mL) in a 20 mL scintillation vial, was added, 2 equiv. of a suspension of *p*TolMgBr (0.252 mmols, 190 μL, from 1.33 mM stock solution in benzene. The stock solution of the Grignard reagent was prepared by removing the THF under vacuum and dissolving the resulting residue in benzene which upon titration, give a final concentration of 1.33 mM.) at 45 °C and allowed to stir. After 1 hour, an aliquot of approximately 0.5 mL was transferred into a Delrin Mössbauer cup and immediately freeze-trapped by placing the cup into a cold well containing condensed argon generated by cooling the cold well with liquid nitrogen. Another aliquot of 1.5 mL was then transferred to a 4 mL scintillation vial which was subsequently sealed and moved in the $N_2$ box, where it was allowed to sit under a nitrogen atmosphere. As soon as it got exposed to $N_2$, the colour changed from dark green to purple. After 1 hour, another aliquot of approximately 0.5 mL was transferred into a Delrin Mössbauer cup and immediately freeze-trapped by placing the cup into liquid nitrogen. The remaining 1 mL of solution was sealed and moved to the argon glovebox. It was then subjected to three freeze-pump-thaw cycles, after which the colour changed again to dark green. The remaining reaction mixture was subsequently transferred into a Delrin Mössbauer cup and immediately freeze-trapped by placing the cup into a cold well containing condensed argon generated by cooling the cold well with liquid nitrogen.

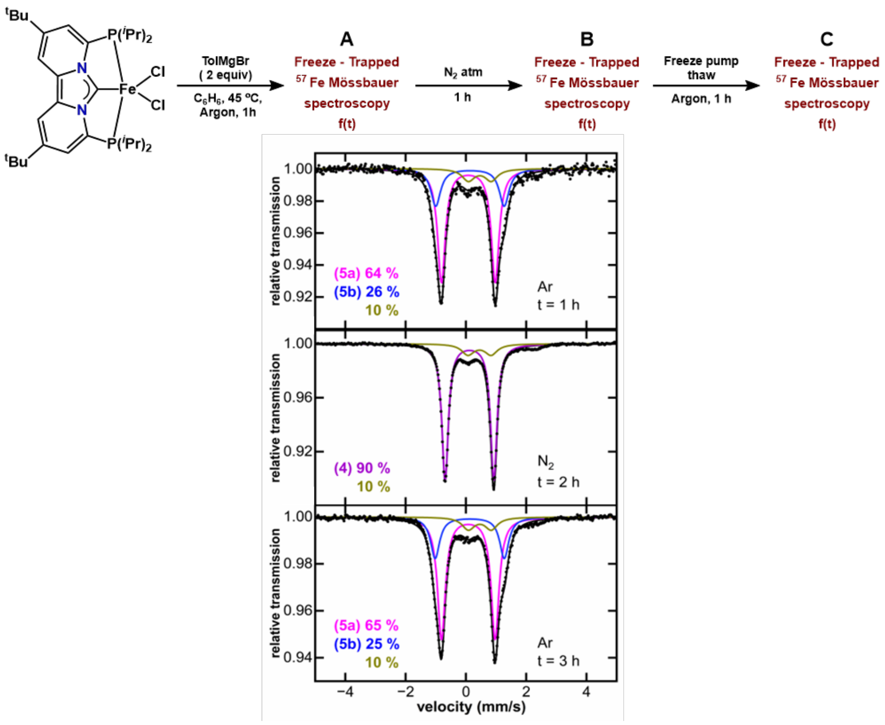


**Figure S45**. Freeze-trapped 80 K $^{57}$Fe Mössbauer spectra of the reaction of **2** with two equiv. of *p*TolMgBr after; (A) one hour under argon atmosphere, (B) one additional hour under $N_2$ atmosphere, and (C) another hour under an argon atmosphere after performing three freeze-pump-thaw cycles to remove excels $N_2$. Mössbauer parameters magenta component $\delta = 0.07$ mm/s $|\Delta E_Q| = 1.80$ mm/s (**5a**) and blue component $\delta = 0.12$ mm/s $|\Delta E_Q| = 2.31$ mm/s (**5b**).

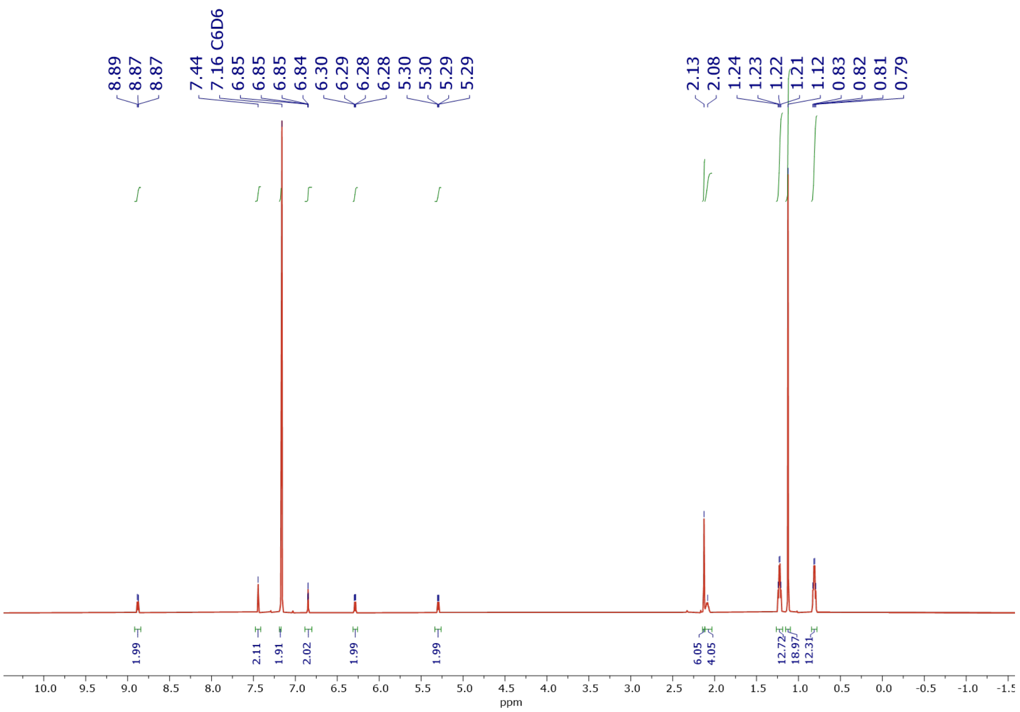


**Figure S46**. $^{1}$H NMR spectra of the reaction of **2** with two equivalents of *p*TolMgBr after exposure of the reaction mixture an atmosphere of $N_2$ for 1 hour.

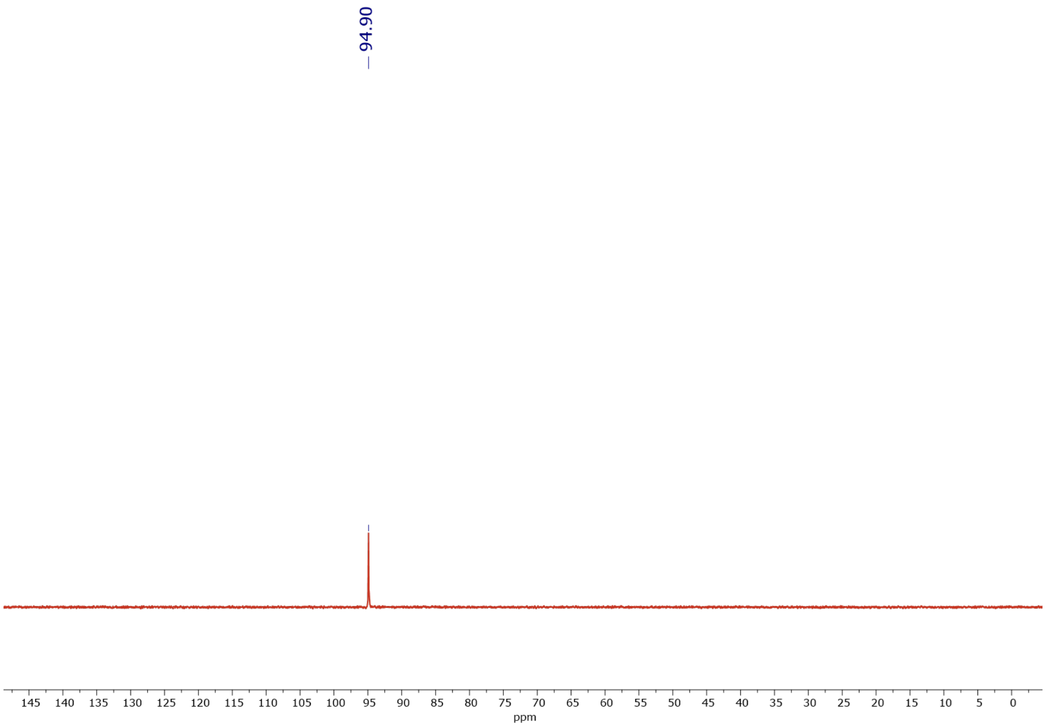


**Figure S47**. $^{31}$P NMR spectra of the reaction of **2** with two equivalents of *p*TolMgBr after exposure of the reaction mixture an atmosphere of $N_2$ for 1 hour.

**Reaction of Complexes 5a/5b with Electrophile**

**General Procedure**. As an example of the used general procedure, the reaction of [(PC$_{NHC}$P)FeCl$_2$] (**2**) with two equiv. of *p*TolMgBr and twenty equiv. of bromobenzene is described. In an Ar-filled glovebox, to a stirred solution of [(PC$_{NHC}$P)FeCl$_2$] (0.042 mmol, 7.5 mg of $^{57}$Fe labeled complex, and 19.5 mg of the unlabeled complex) in benzene (1 mL) in a 4 mL scintillation vial, was added, 2 equivalents of a suspension of *p*TolMgBr (0.084 mmols, 64 µL, from 1.33 mM stock solution in benzene. The stock solution of the Grignard reagent was prepared by removing the THF under vacuum and dissolving the resulting residue in benzene which upon titration, give a final concentration of 1.33 mM.) at 45 °C. The reaction was stirred at 45 °C for 10 minutes, whereafter an aliquot was added (0.5 mL) to Delrin Mössbauer cup to verify the formation of complexes **5a**/**5b** via Freeze trapped $^{57}$Fe Mössbauer spectroscopy. Upon successful formation of **5**a/**5**b, bromobenzene (0.8 mmol, 125.6 mg, 84.0 µL) and dodecane (0.8 mmol, 136.3 mg, 181.8 µL) were added to the reaction mixture. After addition of the electrophile and internal standard, freeze-trapped aliquots were taken at selected time points for Mössbauer analysis.

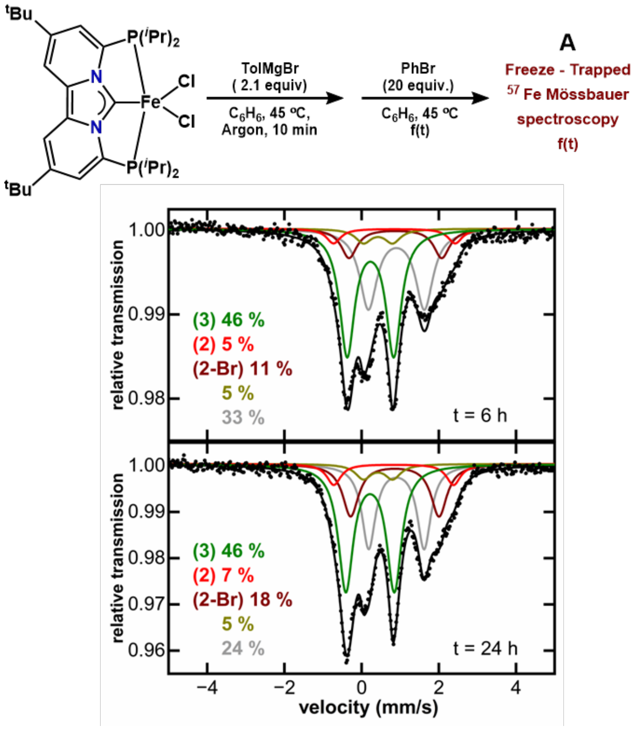


**Figure S48**. Freeze-trapped 80 K $^{57}$Fe Mössbauer spectra of the *in situ* formed iron species upon reaction of **2** with 2 equiv. of *p*TolMgBr for 10 min, followed by the addition of bromobenzene (20 equiv.).

**Procedure for the Catalytic Cross-Coupling.**

Inside an Argon glovebox, an oven-dried J-Young tube was charged with Grignard (1 mmol, 1 M THF, THF was removed under vacuum and the residue redissolved in $C_6D_6$ to obtain a THF-free solution) in $C_6D_6$ (300 μL) and aryl halide (0.5 mmol). [($PC_{NHC}P$)$FeCl_2$] (**2**) or [($PC_{NHC}P$)Fe(0)($N_2$)$_2$)] (**1**) (5 mol%, 0.025 mmol, 100 μL from a 0.25 M stock solution in benzene) was added to reaction mixture and heated at 45 °C for 24 h with occasional shaking. Completion of reaction was monitored by $^1$H NMR spectroscopy. After completion of the reaction, the J-Young tube was brought inside the glovebox, and the reaction mixture was quenched by slow addition of methanol (400 μL). Hereafter the volatiles were removed under reduced pressure, and the obtained residue was redissolved in chloroform (400 μL). The obtained solution was filtered through sort plug silica (2.0 cm) on *Celite* (0.5 cm) to remove the iron catalyst and any other insoluble particulates and salts. The silica was then washed with an additional ethyl acetate (5.0 mL) to collect all the remaining organic products. Dodecane (0.5 mmol) was added to the solution as an internal standard, and an aliquot was taken for GC analysis to determine the yield and selectivity.

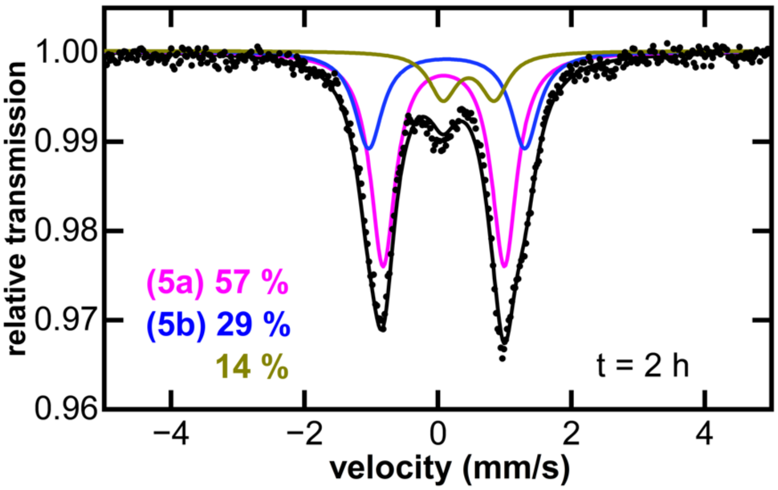


**Figure S49.** Freeze-trapped 80 K $^{57}$Fe Mössbauer spectrum of the iron speciation during catalysis recorded after 2 hours. Mössbauer parameters for magenta component δ = 0.07 mm/s $|\Delta E_Q|$ = 1.80 mm/s (**5a**), blue component δ = 0.12 mm/s $|\Delta E_Q|$ = 2.31 mm/s (**5b**) and dark yellow (impurity) δ = 0.44 mm/s $|\Delta E_Q|$ = 0.74 mm/s, showing there is no formation of any iron ate complexes.

**Table S2**. Reaction optimization.

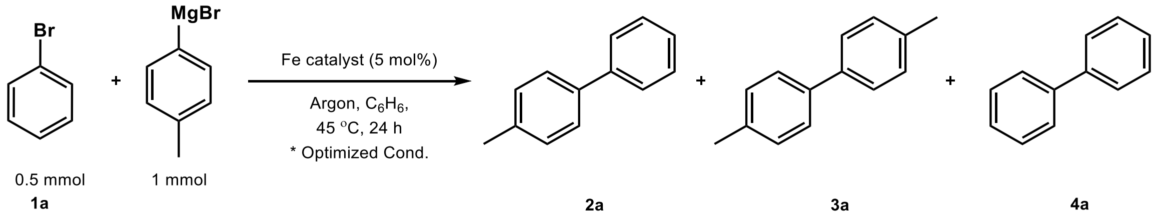


| Entry | Catalyst | Aryl halide | Conditions | Yield | | |
|---|---|---|---|---|---|---|
| | | | | **2a** | **3a** | **4a** |
| **1**[a] | **2** (4 mol%) | Bromobenzene **1a** | Vacuum, 400 mL $C_6D_6$, RT, 24 h | 80 % | 20 % | nd |
| **2**[a] | **2** (4 mol%) | Iodobenzene | Vacuum, 400 mL $C_6D_6$, RT, 45 min | 79 % | 20 % | nd |
| **3**[a] | **2** (5 mol%) | Bromobenzene **1a** | $\mathbf{N_2}$, $C_6H_6$, 45 °C, 24 h | -- | -- | -- |
| **4**[b] | **2** (5 mol%) | Bromobenzene **1a** | *Optimized conditions | 80 % | 15 % | 5% |
| **5**[b] | **2-Br** (5 mol%) | Bromobenzene **1a** | *Optimized conditions | 74 % | 16 % | 7% |
| **6**[b] | **1** (5 mol%) | Bromobenzene **1a** | *Optimized conditions | 83 % | 7 % | 8% |
| **7**[b] | - | Bromobenzene **1a** | *Optimized conditions | -- | -- | -- |
| **8**[b] | **3** (5 mol%) | Bromobenzene **1a** | *Optimized conditions | 75 % | 15 % | 9% |
| **9**[b] | **4** (5 mol%) | Bromobenzene **1a** | *Optimized conditions | 70 % | 13 % | 7% |
| **10**[b] | **5** (5 mol%) | Bromobenzene **1a** | *Optimized conditions | 80 % | 15 % | 6% |
| **11**[a] | **2** (5 mol%) | Bromobenzene **1a** | *Optimized conditions (absence of light) | 75 % | 13 % | nd |

[a]Yields determined by $^1H$ NMR. [b]Yields determined by GC-MS analysis.

## Analysis of Product Yield by Gas Chromatography

**Determination of Response Factor (Rf) from GC Calibration Curve:**

To determine the response factors used for GC-based yield quantification, five calibration solutions were prepared containing equimolar amounts of the independently synthesized biaryl product (obtained via Pd-catalyzed Suzuki coupling) and the internal standard (dodecane). The final concentrations of each analyte in these solutions were 1 mM, 2 mM, 3 mM, 4 mM, and 5 mM, respectively. Ethyl acetate was used as the solvent for all preparations. Each calibration solution was analyzed by gas chromatography under identical conditions. The peak area of the biaryl product was plotted against the peak area of dodecane to generate a calibration curve, and the slope of the resulting linear regression was taken as the response factor ($R_f$). For example, in the case of 4-methylbiphenyl **(3a)**, plotting the peak area at t(r) = 11.6 min against the internal standard at t(r) = 7.02 min across the calibration series yielded a slope of 1.058, giving an $R_f$ value of 1.058 for 4-methylbiphenyl.

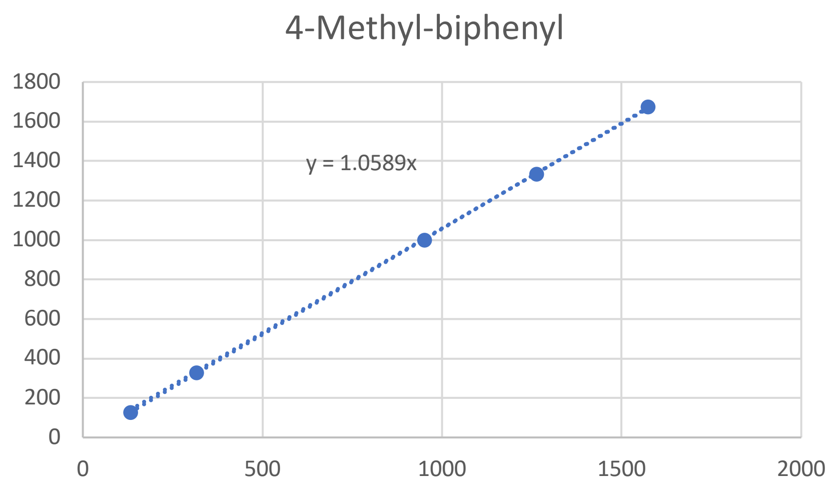


**Figure S50**. GC calibration of 4-methylbiphenyl with dodecane as internal standard

**Calculation of yield from crude reaction mixture**

After completion of the reaction, the organic products were separated from the catalyst according to the general work-up procedure. To the resulting organic phase, 0.5 mmol of dodecane was added as an internal standard, and the total volume was adjusted to 20 mL. The product yield was then determined using the response factor obtained from the GC calibration curve, according to the following relationship:

$$\frac{Area(analyte)}{mmol(analyte)} = Rf \times \frac{Area(Int. sandard)}{mmol(int. standard)}$$

***Rearranging the above equation gives,***

$$mmol(analyte) = Area(analyte) \times \frac{mmol(Int. standard)}{Rf \times Area(Int. standard)}$$

***Substituting the values for 4-Methyl-Biphenyl (3a)***

$$mmol(\text{3a}) = Area(analyte) \times \frac{0.5}{1.058 \times Area(Int. standard)}$$

$$= 1.759\hat{e}9 \times \frac{0.5}{1.058 \times 1.978\hat{e}9} = 0.43$$

***Therefore % yield of 3a*** $= \frac{\mathbf{100}}{\mathbf{0.5}} \times \mathbf{0.43} = \mathbf{86\%}$

**X-Ray Crystallography**

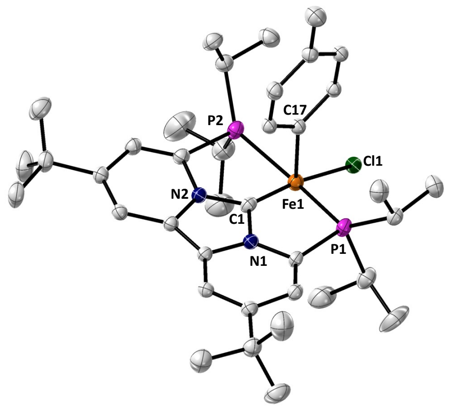


**Figure S51.** Solid state structure of [(PC$_{NHC}$P)Fe(Tol)Cl] (**3**). Ellipsoids are shown at the 30% probability level. Hydrogen atoms and co-crystallized solvent molecules are not shown for clarity.

**Special Refinement Details for 3.** Compound **3** crystallizes in the orthorhombic space group p n m a, with one molecule in the asymmetric unit. The structure was refined as a mixed halide with a single halogen site modeled as disordered between chloride and bromide atoms. The refined occupancies converged to 0.58 (Cl) and 0.42 (Br) . Moreover, iron is also distorted over two independent positions.

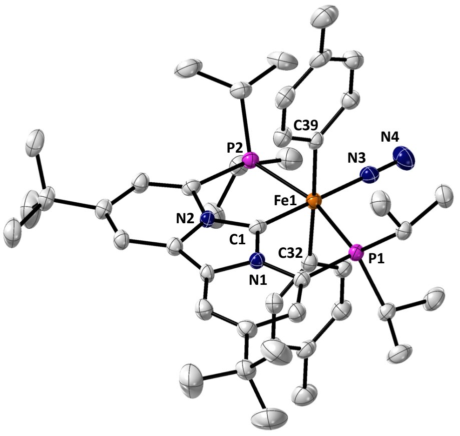


**Figure S52. Solid state structure of [((PC$_{NHC}$P)Fe(Tol)$_2$(N)$_2$] (4).** Ellipsoids are shown at the 30% probability level. Hydrogen atoms and co-crystallized solvent molecules are not shown for clarity.

**Special Refinement Details for 4.** Compound **4** crystallizes in the orthorhombic space group P $2_1$ $2_1$ $2_1$, with one molecule in the asymmetric unit. No further special refinement conditions were necessary.

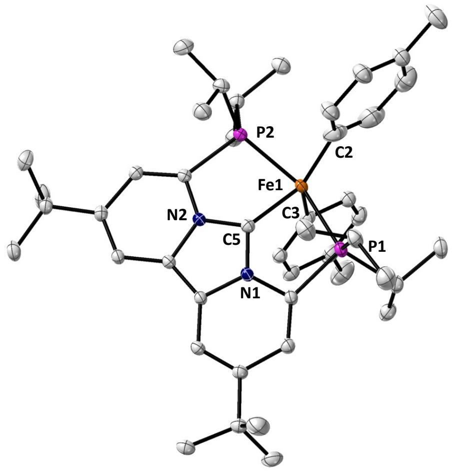


**Figure S53. Solid state structure of [((PC$_{NHC}$P)Fe(Tol)$_2$] (5).** Ellipsoids are shown at the 30% probability level. Hydrogen atoms and co-crystallized solvent molecules are not shown for clarity.

**Special Refinement Details for 5.** Compound **5** crystallizes in the orthorhombic space group P $2_1$ $2_1$ $2_1$, with one molecule in the asymmetric unit. No further special refinement conditions were necessary.

**Table S3**. Selected bond angles and distances for complexes **3**, and **5**.

| **Bond Distances (Å)** | **3** | **5** |
|---|---|---|
| $Fe_1 - C_1$ | 1.842(7) | 1.8133(2) |
| $Fe_1 - Cl$ | 2.447(10) | -- |
| $Fe_1 - P_1$ | 2.2631(16) | 2.2493(4) |
| $Fe_1 - P_2$ | 2.2632(16) | 2.2493(4) |
| $Fe_1 - C_{17}$ | 1.869(9) | 1.969(2) |
| $Fe_1 - C_{17A}$ | -- | 2.014(3) |
| **Bond Angles (°)** | | |
| $P_1$ $Fe_1$ $P_2$ | 159.6(3) | 160.98(2) |
| $C_1$ $Fe_1$ $P_1$ | 80.55(11) | 81.026(12) |
| $C_{17}$ $Fe_1$ $C_1$ | 90.4(3) | 88.40(9) |
| $C_{17}$ $Fe_1$ $C_{17A}$ | -- | 139.61(13) |
| $C_1$ $Fe_1$ Cl | 139.9(5) | -- |
| $C_{17}$ $Fe_1$ Cl | 129.7(5) | -- |

**Table S4**. Selected bond angles and distances for complex 4

| **Bond Distances (Å)** | **4** |
|---|---|
| $Fe_1 - C_1$ | 1.844(3) |
| $Fe_1 - P_1$ | 2.2788(14) |
| $Fe_1 - P_2$ | 2.2737(14) |
| $Fe_1 - C_{32}$ | 2.069(3) |
| $Fe_1 - C_{39}$ | 2.075(3) |
| $Fe_1 - N_3$ | 1.817(3) |
| **Bond Angles (°)** | |
| $P_1$ $Fe_1$ $P_2$ | 162.47(4) |
| $C_1$ $Fe_1$ $P_1$ | 81.50(15) |
| $C_1$ $Fe_1$ $P_2$ | 80.99(15) |
| $C_{32}$ $Fe_1$ $C_1$ | 88.34 (14) |
| $C_{39}$ $Fe_1$ $C_1$ | 88.94(14) |
| $N_3$ $Fe_1$ $C_1$ | 179.11(17) |
| $N_3$ $Fe_1$ $C_{32}$ | 90.34(13) |
| $N_3$ $Fe_1$ $C_{39}$ | 92.37(12) |
| $C_{32}$ $Fe_1$ $C_{39}$ | 177.26(14) |

**Table S5**. Crystal and refinement data for complexes **3**, **4,** and **5**.

| | **3** | **4** | **5** |
|---|---|---|---|
| Identification code (CCDC) | 2512961 | 2512962 | 2512963 |
| Empirical formula | $C_{50}H_{69}Br_{0.42}Cl_{0.58}FeN_2P_2$ | $C_{51}H_{70}FeN_4P_2$ | $C_{51}H_{73}FeN_2P_2$ |
| Formula weight | 869.76 | 856.90 | 831.9 |
| Temperature/K | 100.15 | 100.15 | 150.00(10) |
| Crystal system | orthorhombic | orthorhombic | orthorhombic |
| Space group | Pnma | $P2_12_12_1$ | Pnma |
| a/Å | 20.2826(18) | 14.4884(9) | 24.6523(2) |
| b/Å | 14.6106(16) | 15.3950(9) | 14.55450(10) |
| c/Å | 16.4306(15) | 21.3959(13) | 13.08320(10) |
| α/° | 90 | 90 | 90 |
| β/° | 90 | 90 | 90 |
| γ/° | 90 | 90 | 90 |
| Volume/Å$^3$ | 4869.1(8) | 4772.3(5) | 4694.28(6) |
| Z | 4 | 4 | 4 |
| $\rho_{calc}$g/cm$^3$ | 1.186 | 1.193 | 1.173 |
| μ/mm$^{-1}$ | 0.782 | 0.421 | 3.471 |
| F(000) | 1854 | 1840.0 | 1796.0 |
| Crystal size/mm$^3$ | 0.24 × 0.21 × 0.18 | 0.21 × 0.15 × 0.12 | 0.24 × 0.19 × 0.11 |
| Radiation | MoKα (λ = 0.71073) | MoKα (λ = 0.71073) | CuKα (λ = 1.5418) |
| 2Θ range for data collection/° | 3.19 to 50.224 | 3.26 to 50.148 | 7.172 TO148.98 |
| Index ranges | $-23 \leq h \leq 24$, $-17 \leq k \leq 16$, $-19 \leq l \leq 19$ | $-16 \leq h \leq 17$, $-18 \leq k \leq 18$, $-25 \leq l \leq 25$ | $-30 \leq h \leq 26$, $-18 \leq k \leq 18$, $-16 \leq l \leq 16$ |
| Reflections collected | 22443 | 38154 | 54656 |
| Independent reflections | 4518 [$R_{int}$ = 0.0677, $R_{sigma}$ = 0.0542] | 8425 [$R_{int}$ = 0.0617, $R_{sigma}$ = 0.0568] | 5004 [$R_{int}$ = 0.0379, $R_{sigma}$ = 0.0176 ] |
| Data/restraints/parameters | 4518/332/299 | 8425/533/539 | 5004/437/309 |
| Goodness-of-fit on F$^2$ | 1.066 | 1.010 | 1.024 |
| Final R indexes [I>=2σ (I)] | $R_1$ = 0.0640, $wR_2$ = 0.1613 | $R_1$ = 0.0388, $wR_2$ = 0.0773 | $R_1$ = 0.0343, $wR_2$ = 0.0971 |
| Final R indexes [all data] | $R_1$ = 0.1107, $wR_2$ = 0.1914 | $R_1$ = 0.0607, $wR_2$ = 0.0858 | $R_1$ = 0.0380, $wR_2$ = 0.1008 |
| Largest diff. peak/hole / e Å$^{-3}$ | 1.29/-0.60 | 0.28/-0.36 | 0.50/-0.36 |
| Flack parameter | -- | -0.024(10) | -- |

## Computational Details

### General Computational Details.

The geometry optimization of all intermediates and transition states across all spin states were performed using ORCA 6.0.1.[9, 10] For optimization, the M06L[11] functional was employed, using the resolution of identity and chain-of-spheres approximations to evaluate the Coulomb and exchange integrals (RIJCOSX),[12] Grimme's empirical D3 dispersion correction incorporating Becke-Johnson damping.[13, 14] The def2-TZVP basis set was used for Fe atoms and def2-SVP was used for all other elements.[15-17] Wavefunction stability tests were performed for all intermediates and transition states along the singlet, triplet and quintet PESs using the keywords "StabPerform True". Frequencies analyses on the optimized geometries were performed at the same level of theory to confirm true minima for intermediates ($N_{imag} = 0$) and saddle-points ($N_{imag} = 1$) for transition states, and to obtain zero-point energy, enthalpy, and entropic corrections.

Energies for the optimized geometries were obtained with single-point calculations at the M06L-D3/def2-TZVP level of theory with the SMD[18] solvation model for benzene. All reported energies include the zero-point energy, enthalpy, and entropic corrections obtained from the vibrational frequency calculations mentioned above.

**Input Templates**

## 1. Full Geometry Optimisation

### i) Intermediates

```
! opt freq m06l d3zero def2-svp def2/J RIJCOSX Largeprint
%basis newgto Fe "def2-TZVP" end end
%geom Trust -0.1 end
%scf MaxIter 200 end
* xyz 0 <m>
coordinates
*
```

<m> is a placeholder for multiplicity, *coordinates* are the Cartesian coordinates in XMOL format.

### ii) Transition states

```
! OptTS freq m06l d3zero def2-svp def2/J RIJCOSX Largeprint
%basis newgto Fe "def2-TZVP" end end
%geom
 Trust -0.1
 Calc_Hess true
 end
%scf MaxIter 200 end
* xyz 0 <m>
coordinates
*
```

<m> is a placeholder for multiplicity, *coordinates* are the Cartesian coordinates in XMOL format.

## 2. Single-point Calculations including solvation

```
! m06l d3zero def2-tzvp def2/J RIJCOSX SMD(benzene)
%scf MaxIter 200 end
* xyz 0 <m>
coordinates
*
```

<m> is a placeholder for multiplicity, *coordinates* are the Cartesian coordinates in XMOL format.

## Benchmarking

To choose the appropriate computational method, we benchmarked complexes **2**, **3** and **6** against their crystal structure geometries using different levels of theory:

- PBE-D3BJ (modified to include 15% HF exchange),
- PBE0-D3BJ (modified to include 15% HF exchange),
- ωB97xD (modified to include 15% HF exchange),
- B3PW91**-D3BJ (modified to include 15% HF exchange),
- B3LYP (modified to include 15% HF exchange),
- PBE-D3BJ (default)
- PBE0-D3BJ (default)
- B3LYP (default)
- M06-2X (default)
- M06-L (default)

As noted above, some of the functionals were specifically altered to modify the percentage of HF exchange, either by increasing or decreasing the default value. By default, PBE has 0% HF exchange, PBE0 has 25%, B3LYP and B3PW91 have 20%, and ωB97xD has a short-range HF exchange percentage between 22-15%. This modification was made because the addition of HF exchange leads to an artifactual systematic lowering of the high-spin states, due to the inclusion of Fermi correlations but not Coulomb correlations. Previous studies[29-32] indicate that using a hybrid functional without modifying the exact HF exchange leads to over-stabilization of the triplet and quintet spin-states relative to the singlet state. Setting the HF exchange to approximately 10–15% has been demonstrated to predict the ground state correctly (as validated experimentally), while maintaining the accuracy of the original functional.[33]

In all cases, the def2SVP basis set was used for C, H, N, P, O, and def2TZVP was used for Fe.[19-28] The root mean square deviation of atomic positions (RMSD) between the crystal structure and the optimized geometry of the complex (in its ground state) were compared We also considered that the most relevant part of the molecule, and the one where method dependency is most likely to occur, is around the Fe center. Therefore, we also calculated the RMSD only around the Fe center. The RMSD values are detailed in **Tables S6–S8**. The values are color-coded, such that the darker the color, the greater the RMSD.

**Table S6**. RMSD values for complex **2**, compared to the crystal structure (ground state = quintet).

| **Tested Functional** | **RMSD (all atoms)** | **RMSD (Fe, $P_1$, $P_2$, $C_1$, $Cl_1$, $Cl_2$)** |
|---|---|---|
| PBE-D3BJ (with 15% HF exchange) | 0.714 | 0.326 |
| PBE0-D3BJ (with 15% HF exchange) | 0.501 | 0.217 |
| ωB97xD (with 15% HF exchange) | 0.475 | 0.207 |
| B3PW91**-D3BJ (with 15% HF exchange) | 0.528 | 0.215 |
| B3LYP (with 15% HF exchange) | 0.461 | 0.194 |
| PBE-D3BJ | 0.45 | 0.198 |
| PBE0-D3BJ | 0.455 | 0.189 |
| B3LYP | 0.457 | 0.188 |
| M06-2X | 0.494 | 0.187 |
| M06-L | 0.394 | 0.196 |

**Table S7**. RMSD values for complex **3**, compared to the crystal structure (ground state = singlet).

| **Tested Functional** | **RMSD (all atoms)** | **RMSD (Fe, $P_1$, $P_2$, $C_1$, $Cl_1$, $C_{17}$)** |
|---|---|---|
| PBE-D3BJ (with 15% HF exchange) | 0.312 | 0.074 |
| PBE0-D3BJ (with 15% HF exchange) | 0.334 | 0.099 |
| ωB97xD (with 15% HF exchange) | 0.362 | 0.098 |
| B3PW91**-D3BJ (with 15% HF exchange) | 0.229 | 0.098 |
| B3LYP (with 15% HF exchange) | 0.337 | 0.083 |
| PBE-D3BJ | 0.321 | 0.077 |
| PBE0-D3BJ | 0.237 | 0.101 |
| B3LYP | 0.243 | 0.085 |
| M06-2X | 0.332 | 0.198 |
| M06-L | 0.289 | 0.086 |

**Table S8**. RMSD values for complex **5**, compared to the crystal structure (ground state = singlet).

| **Tested Functional** | **RMSD (all atoms)** | **RMSD (Fe, $P_1$, $P_2$, $C_1$, $Cl_1$, $Cl_2$)** |
|---|---|---|
| PBE-D3BJ (with 15% HF exchange) | 0.399 | 0.074 |
| PBE0-D3BJ (with 15% HF exchange) | 0.411 | 0.129 |
| ωB97xD (with 15% HF exchange) | 0.396 | 0.087 |
| B3PW91**-D3BJ (with 15% HF exchange) | 0.425 | 0.13 |
| B3LYP (with 15% HF exchange) | 0.409 | 0.123 |
| PBE-D3BJ | 0.39 | 0.039 |
| PBE0-D3BJ | 0.447 | 0.123 |
| B3LYP | 0.409 | 0.092 |
| M06-2X | 0.483 | 0.152 |
| M06-L | 0.453 | 0.126 |

We further compared the bond lengths around the Fe center (**Tables S9-S11**). In these tables, the data are color-coded, such that darker color indicates greater the discrepancy between the computed and measured values.

**Table S9**. Calculated and experimentally determined bond distances for complex **2** (spin-state: quintet).

| **Complex 2 (Quintet)** | **Bond Distances (Å)** | | | | |
|---|---|---|---|---|---|
| | **Fe-P$_1$** | **Fe-P$_2$** | **Fe-C$_1$** | **Fe-Cl$_1$** | **Fe-Cl$_2$** |
| **Crystal Structure** | 2.782 | 2.765 | 2.064 | 2.265 | 2.293 |
| | | | | | |
| **Tested Functional** | | | | | |
| PBE-D3BJ (with 15% HF exchange) | 2.402 | 2.403 | 1.902 | 2.132 | 2.071 |
| PBE0-D3BJ (with 15% HF exchange) | 2.577 | 2.577 | 1.986 | 2.221 | 2.199 |
| ωB97xD (with 15% HF exchange) | 2.575 | 2.575 | 1.981 | 2.247 | 2.227 |
| B3PW91**-D3BJ (with 15% HF exchange) | 2.564 | 2.564 | 1.968 | 2.194 | 2.171 |
| B3LYP (with 15% HF exchange) | 2.672 | 2.667 | 2.053 | 2.306 | 2.297 |
| PBE-D3BJ | 2.607 | 2.609 | 1.998 | 2.293 | 2.285 |
| PBE0-D3BJ | 2.673 | 2.67 | 2.067 | 2.306 | 2.298 |
| B3LYP | 2.696 | 2.689 | 2.071 | 2.311 | 2.302 |
| M06-2X | 2.771 | 2.766 | 2.162 | 2.341 | 2.326 |
| M06-L | 2.696 | 2.729 | 2.079 | 2.32 | 2.289 |

**Table S10**. Calculated and experimentally determined bond distances for complex **3** (spin-state: singlet).

| **Complex 3 (Singlet)** | **Bond Distances (Å)** | | | | |
|---|---|---|---|---|---|
| | **Fe-P$_1$** | **Fe-P$_2$** | **Fe-C$_1$** | **Fe-Cl$_1$** | **Fe-C$_{17}$** |
| **Crystal Structure** | 2.264 | 2.263 | 1.842 | 2.312 | 1.869 |
| | | | | | |
| **Tested Functional** | | | | | |
| PBE-D3BJ (with 15% HF exchange) | 2.272 | 2.271 | 1.815 | 2.294 | 1.923 |
| PBE0-D3BJ (with 15% HF exchange) | 2.231 | 2.229 | 1.797 | 2.262 | 1.894 |
| ωB97xD (with 15% HF exchange) | 2.318 | 2.317 | 1.846 | 2.303 | 1.954 |
| B3PW91**-D3BJ (with 15% HF exchange) | 2.24 | 2.239 | 1.809 | 2.273 | 1.903 |
| B3LYP (with 15% HF exchange) | 2.265 | 2.264 | 1.816 | 2.288 | 1.919 |
| PBE-D3BJ | 2.24 | 2.239 | 1.794 | 2.278 | 1.901 |
| PBE0-D3BJ | 2.257 | 2.256 | 1.817 | 2.276 | 1.914 |
| B3LYP | 2.279 | 2.281 | 1.829 | 2.295 | 1.929 |
| M06-2X | 2.431 | 2.425 | 1.941 | 2.315 | 1.995 |
| M06-L | 2.277 | 2.275 | 1.816 | 2.309 | 1.923 |

**Table S11**. Calculated and experimentally determined bond distances for complex **5** (spin-state: singlet).

| **Complex 6 (Singlet)** | **Bond Distances (Å)** | | | | |
|---|---|---|---|---|---|
| | **Fe-P$_1$** | **Fe-P$_2$** | **Fe-C$_1$** | **Fe-Cl$_1$** | **Fe-C$_{17}$** |
| **Crystal Structure** | 2.249 | 2.249 | 1.814 | 1.970 | 2.017 |
| | | | | | |
| **Tested Functional** | | | | | |
| PBE-D3BJ (with 15% HF exchange) | 2.263 | 2.263 | 1.815 | 1.982 | 1.997 |
| PBE0-D3BJ (with 15% HF exchange) | 2.231 | 2.23 | 1.804 | 1.99 | 1.939 |
| ωB97xD (with 15% HF exchange) | 2.308 | 2.31 | 1.845 | 2.01 | 2.017 |
| B3PW91**-D3BJ (with 15% HF exchange) | 2.236 | 2.237 | 1.812 | 1.99 | 1.944 |
| B3LYP (with 15% HF exchange) | 2.265 | 2.265 | 1.817 | 2.006 | 1.958 |
| PBE-D3BJ | 2.229 | 2.226 | 1.798 | 1.929 | 2.013 |
| PBE0-D3BJ | 2.253 | 2.254 | 1.821 | 2 | 1.951 |
| B3LYP | 2.27 | 2.271 | 1.826 | 1.989 | 1.99 |
| M06-2X | 2.413 | 2.415 | 1.944 | 2.059 | 2.036 |
| M06-L | 2.278 | 2.272 | 1.822 | 2.026 | 1.971 |

Based on the benchmarking results, we elected to continue with the M06L functional, which satisfactorily reproduced the experimental data. While several functionals produced comparable results, M06L showed the lowest RMSD for complex **2** in its quintet ground state, which led us to prioritize it. In addition, there is ample literature precedent, which has shown that M06L is known to explicitly handle both main-group and transition-metal bonding, kinetics, and noncovalent interactions and also shows efficiency in large transition metal chemistry.[11, 34, 35] Thus, we settled on the DFT computational method *M06L-D3/def2SVP* for C, H, N, P, O and *def2TZVP* for Fe throughout our calculations.

## Electronic Structure determination of Complex 5

**Table S12**. RMSD values for complex **5**, compared to the crystal structure, for various spin-states.

| Tested Functional | RMSD (all atoms) | | |
|---|---|---|---|
| | Singlet | Triplet | Quintet |
| PBE-D3BJ (with 15% HF exchange) | 0.399 | 0.501 | 0.84 |
| PBE0-D3BJ (with 15% HF exchange) | 0.411 | 0.512 | 0.812 |
| ωB97xD (with 15% HF exchange) | 0.396 | 0.492 | 0.976 |
| B3PW91**-D3BJ (with 15% HF exchange) | 0.425 | 0.534 | 0.84 |
| B3LYP (with 15% HF exchange) | 0.409 | 0.523 | 0.926 |
| PBE-D3BJ | 0.39 | 0.476 | 0.69 |
| PBE0-D3BJ | 0.447 | 0.521 | 0.918 |
| B3LYP | 0.409 | 0.531 | 0.921 |
| M06-2X | 0.483 | 0.567 | 0.412 |
| M06-L | 0.453 | 0.562 | 0.962 |

**Table S13**. Calculated and experimentally determined bond distances for complex **5** for various spin-states

| Complex-5 | Relative Electronic Energy (kcal $mol^{-1}$) | RMSD (all atoms) | RMSD (Fe, P2, P3, C18, C19, C65) | Fe-$P_2$ | Fe-$P_3$ | Fe-$C_{18}$ | Fe-$C_{19}$ | Fe-$C_{65}$ |
|---|---|---|---|---|---|---|---|---|
| **Singlet** | 0 | 0.453 | 0.126 | 2.278 | 2.272 | 1.822 | 2.026 | 1.971 |
| **Triplet** | 6.12 | 0.562 | 0.140 | 2.242 | 2.259 | 1.953 | 2.049 | 2.022 |
| **Quintet** | 7.89 | 0.962 | 0.370 | 2.821 | 2.670 | 2.097 | 2.072 | 2.066 |
| **Crystal Structure 5** | | | | 2.249 | 2.249 | 1.814 | 1.970 | 2.017 |

## Energetics of the Proposed Catalytic Cycle

**Table S14**. Calculated free energy (G) and total enthalpy (H) in Hartree for the proposed cross-coupling.

**Transmetalation**

| Complex | Mg-assisted Solvent Electronic Energy ($E_{SCF}$) (Hartree) | Mg-assisted Thermal Correction (Hartree) | Mg-assisted Gibbs Free Energy (G) (Hartree) | Mg-assisted Total Enthalpy (H) (Hartree) | Non Mg-assisted Solvent Electronic Energy ($E_{SCF}$) (Hartree) | Non Mg-assisted Thermal Correction (Hartree) | Non Mg-assisted Gibbs Free Energy (G) (Hartree) | Non Mg-assisted Total Enthalpy (H) (Hartree) |
|---|---|---|---|---|---|---|---|---|
| **2** | --- | --- | --- | --- | -4188.260789 | 0.67716931 | -4187.58362 | -4185.517078 |
| **Int1** | --- | --- | --- | --- | -7233.551763 | 0.78848082 | -7232.763283 | -7230.004731 |
| **TS1** | --- | --- | --- | --- | -7233.530678 | 0.79140083 | -7232.739278 | -7229.988101 |
| **Int2** | --- | --- | --- | --- | -7233.557993 | 0.78706024 | -7232.770933 | -7230.006976 |
| **3** | --- | --- | --- | --- | -3999.007275 | 0.79564677 | -3998.211628 | -3996.002605 |
| **Int3** | --- | --- | --- | --- | -7044.296374 | 0.90093962 | -7043.395434 | -7040.484398 |
| **TS2** | --- | --- | --- | --- | -7044.27636 | 0.9067364 | -7043.369624 | -7040.469241 |
| **Int4** | --- | --- | --- | --- | -7044.287254 | 0.9023103 | -7043.384944 | -7040.483146 |
| **5** | --- | --- | --- | --- | -3809.738308 | 0.90546847 | -3808.832839 | -3806.474296 |

**Homo-Coupling and Radical Formation**

| Complex | Mg-assisted Solvent Electronic Energy ($E_{SCF}$) (Hartree) | Mg-assisted Thermal Correction (Hartree) | Mg-assisted Gibbs Free Energy (G) (Hartree) | Mg-assisted Total Enthalpy (H) (Hartree) | Non Mg-assisted Solvent Electronic Energy ($E_{SCF}$) (Hartree) | Non Mg-assisted Thermal Correction (Hartree) | Non Mg-assisted Gibbs Free Energy (G) (Hartree) | Non Mg-assisted Total Enthalpy (H) (Hartree) |
|---|---|---|---|---|---|---|---|---|
| **5** | --- | --- | --- | --- | -3809.738308 | 0.90546847 | -3808.832839 | -3806.474296 |
| **TS3** | -11963.862 | 0.98800322 | -11962.874 | -9845.5441 | -6615.4590 | 0.98769822 | -6614.4713 | -6611.5359 |
| **7** | -11732.253 | 0.89835884 | -11731.355 | -11720.066 | -6383.8413 | 0.89992213 | -6382.9413 | -6380.2598 |
| **TS4** | -11732.244 | 0.89734185 | -11731.346 | -11727.950 | -6383.8230 | 0.89960048 | -6382.9234 | -6380.2265 |
| **8** | -11190.249 | 0.67803583 | -11189.571 | -11186.781 | -5841.8366 | 0.68054608 | -5841.1560 | -5839.0732 |
| **TS5** | -13995.997 | 0.75963016 | -13995.238 | -13991.870 | -8647.5749 | 0.76329068 | -8646.8116 | -8644.1545 |
| **2** | --- | --- | --- | --- | -8415.9687 | 0.6773315 | -8415.2914 | -8412.8916 |

**Cross-Coupling**

| Complex | Mg-assisted Solvent Electronic Energy ($E_{SCF}$) (Hartree) | Mg-assisted Thermal Correction (Hartree) | Mg-assisted Gibbs Free Energy (G) (Hartree) | Mg-assisted Total Enthalpy (H) (Hartree) | Non Mg-assisted Solvent Electronic Energy ($E_{SCF}$) (Hartree) | Non Mg-assisted Thermal Correction (Hartree) | Non Mg-assisted Gibbs Free Energy (G) (Hartree) | Non Mg-assisted Total Enthalpy (H) (Hartree) |
|---|---|---|---|---|---|---|---|---|
| **3** | --- | --- | --- | --- | -6112.8627 | 0.79462023 | -6112.0681 | -6109.6926 |
| **Int9 ($3-MgBr_2$)** | -11461.270 | 0.78843579 | -11460.481 | -11457.387 | --- | --- | --- | --- |
| **TS6** | -11692.881 | 0.87389937 | -11692.007 | -11688.657 | -6344.4726 | 0.87828533 | -6343.5943 | -6340.9624 |
| **7’** | -11692.930 | 0.87356061 | -11692.057 | -11688.705 | -6344.5190 | 0.87641186 | -6343.6426 | -6341.0076 |
| **TS7** | -11692.918 | 0.87357174 | -11692.044 | -11688.704 | -6344.4960 | 0.87515041 | -6343.6208 | -6340.9883 |
| **8’** | -11190.249 | 0.67803583 | -11189.571 | -11186.781 | -5841.8366 | 0.68054608 | -5841.1560 | -5839.0732 |
| **TS8** | -13995.997 | 0.75963016 | -13995.238 | -13991.870 | -8647.5749 | 0.76329068 | -8646.8116 | -8644.1545 |
| **2** | --- | --- | --- | --- | -8415.9687 | 0.6773315 | -8415.2914 | -8412.8916 |

**Table S15**. Calculated free energy profiles (ΔG) in kcal/mol for the proposed cross-coupling

| Transmetallation | | | |
|---|---|---|---|
| **Complex** | **ΔG (Mg-assisted) (kcal/mol)** | **ΔG (Non Mg-assisted) (kcal/mol)** | **Spin-state** |
| **2** | --- | 0 | quintet |
| **Int1** | --- | -12.22 | quintet |
| **TS1** | --- | 2.85 | quintet |
| **Int2** | --- | 17.01 | quintet |
| **3** | --- | -4.61 | singlet |
| **Int3** | --- | 19.42 | singlet |
| **TS2** | --- | -3.23 | singlet |
| **Int4** | --- | -12.84 | singlet |
| **5** | --- | -4.95 | singlet |
| **Homo-Coupling and Radical Formation** | | | |
| **Complex** | **ΔG (Mg-assisted) (kcal/mol)** | **ΔG (Non Mg-assisted) (kcal/mol)** | **Spin-state** |
| **5** | --- | -4.95 | singlet |
| **TS3** | 25.10 | 38.4 | triplet |
| **7** | 0.44 | 20.481 | quartet |
| **TS4** | 5.91 | 31.7 | quartet |
| **8** | -52.42 | -31.23 | doublet |
| **TS5** | -26.4 | 1.4 | doublet |
| **2** | -42.03 | -22.6 | quintet |
| **Cross-Coupling** | | | |
| **Complex** | **ΔG (Mg-assisted) (kcal/mol)** | **ΔG (Non Mg-assisted) (kcal/mol)** | **Spin-state** |
| **3** | 0 | 0 | singlet |
| **Int-9 ($3\text{-}MgBr_2$)** | -19.7 | --- | singlet |
| **TS6** | 1.01 | 20.3 | doublet |
| **7’** | -30.4 | -10.01 | quartet |
| **TS7** | -22.5 | 3.6 | quartet |
| **8’** | -80.6 | -59.8 | doublet |
| **TS8** | -54.9 | -27.1 | doublet |
| **2** | -70.6 | -51.2 | quintet |

**Energetics of the Alternative Mechanistic Proposal for Iron Catalyzed Aryl-Aryl Cross-Coupling.**

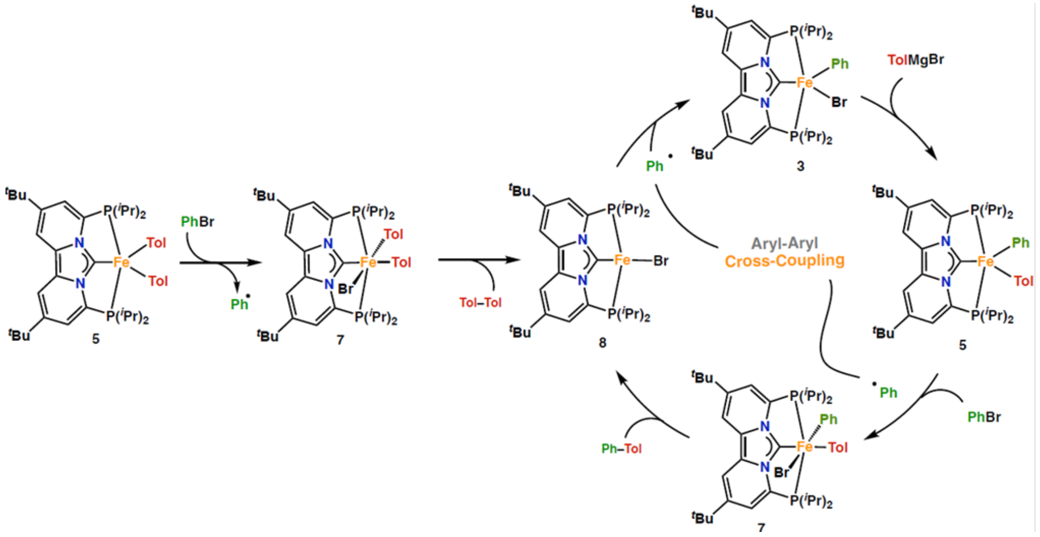


**Figure S54**. Schematic representation of alternative catalytic cycle, where homolytic bond cleavage and radical propagation is facilitated by the heteroleptic iron bis-aryl species (**5**)

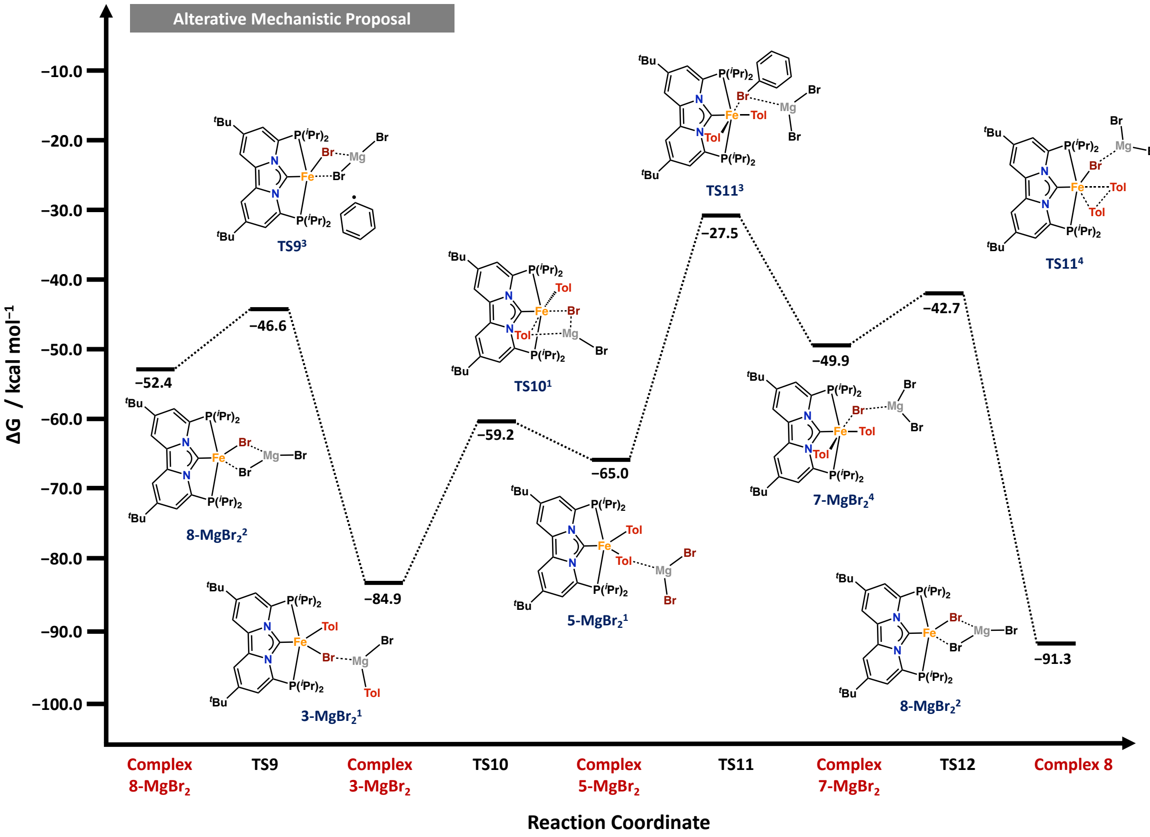


**Scheme S1**. Calculated free energy profile (ΔG) in kcal mol$^{-1}$ for the iron catalyzed aryl-aryl cross-coupling via the alternative mechanistic proposal (**Figure S54**). Reported energies are relative to the energy **Complex 2** set at 0.0 kcal/mol. See the supporting information for more computational details.

**Table S16**. Calculated free energy (G) and total enthalpy (H) in Hartree for the proposed alternative cross-coupling cycle (**Figure S54**)

| Homo-Coupling and Radical Formation | | | | | | | | |
|---|---|---|---|---|---|---|---|---|
| | Mg-assisted | | | | Non Mg-assisted | | | |
| **Complex** | **Solvent Electronic Energy ($E_{SCF}$) (Hartree)** | **Thermal Correction (Hartree)** | **Gibbs Free Energy (G) (Hartree)** | **Total Enthalpy (H) (Hartree)** | **Solvent Electronic Energy ($E_{SCF}$) (Hartree)** | **Thermal Correction (Hartree)** | **Gibbs Free Energy (G) (Hartree)** | **Total Enthalpy (H) (Hartree)** |
| **5** | --- | --- | --- | --- | -3809.738308 | 0.90546847 | -3808.832839 | -3806.474296 |
| **TS3** | -11963.862 | 0.98800322 | -11962.874 | -9845.5441 | -6615.4590 | 0.98769822 | -6614.4713 | -6611.5359 |
| **7** | -11732.253 | 0.89835884 | -11731.355 | -11720.066 | -6383.8413 | 0.89992213 | -6382.9413 | -6380.2598 |
| **TS4** | -11732.244 | 0.89734185 | -11731.346 | -11727.950 | -6383.8230 | 0.89960048 | -6382.9234 | -6380.2265 |
| **8** | -11190.249 | 0.67803583 | -11189.571 | -11186.781 | -5841.8366 | 0.68054608 | -5841.1560 | -5839.0732 |

| Cross-Coupling | | | | | | | | |
|---|---|---|---|---|---|---|---|---|
| | Mg-assisted | | | | Non Mg-assisted | | | |
| **Complex** | **Solvent Electronic Energy ($E_{SCF}$) (Hartree)** | **Thermal Correction (Hartree)** | **Gibbs Free Energy (G) (Hartree)** | **Total Enthalpy (H) (Hartree)** | **Solvent Electronic Energy ($E_{SCF}$) (Hartree)** | **Thermal Correction (Hartree)** | **Gibbs Free Energy (G) (Hartree)** | **Total Enthalpy (H) (Hartree)** |
| **8** | -11190.249 | 0.67803583 | -11189.571 | -11186.781 | -5841.8366 | 0.68054608 | -5841.1560 | -5839.0732 |
| **TS9** | -11421.880 | 0.75962878 | -11421.121 | -11418.072 | --- | --- | --- | --- |
| **3** | --- | --- | --- | --- | -6073.5371 | 0.767109 | -6072.7700 | -6070.4377 |
| **TS10** | --- | --- | --- | --- | -9118.7991 | 0.87991916 | -9117.9192 | -9114.8956 |
| **5** | --- | --- | --- | --- | -3770.4129 | 0.88078025 | -3769.5322 | -3767.2205 |
| **TS11** | -11924.536 | 0.96050563 | -11923.576 | -11919.974 | -6576.1331 | 0.96183082 | -6575.1713 | -6572.2810 |
| **7** | -11692.926 | 0.87323812 | -11692.053 | -11688.703 | -6344.5153 | 0.87655318 | -6343.6387 | -6341.0050 |
| **TS12** | -11692.916 | 0.87448079 | -11692.042 | -11688.693 | -6344.4960 | 0.87515041 | -6343.6208 | -6340.9717 |
| **8** | -11190.249 | 0.67803583 | -11189.571 | -11186.781 | -5841.8366 | 0.68054608 | -5841.1560 | -5839.0732 |

**Table S17**. Calculated free energy profiles (ΔG) in kcal/mol for the proposed alternative cross-coupling cycle (**Figure S54**)

| Homo-Coupling and Radical Formation | | | |
|---|---|---|---|
| **Complex** | **ΔG (Mg-assisted) (kcal/mol)** | **ΔG (Non Mg-assisted) (kcal/mol)** | **Spin-state** |
| **5** | --- | -4.95 | singlet |
| **TS3** | 25.10 | 38.4 | triplet |
| **7** | 0.44 | 20.481 | quartet |
| **TS4** | 5.91 | 31.7 | quartet |
| **8** | -52.42 | -31.23 | doublet |
| **Cross-Coupling** | | | |
| **Complex** | **ΔG (Mg-assisted) (kcal/mol)** | **ΔG (Non Mg-assisted) (kcal/mol)** | **Spin-state** |
| **8** | 0 | --- | doublet |
| **TS9** | 5.80 | --- | triplet |
| **3** | --- | -13.6 | singlet |
| **TS10** | --- | -6.74 | singlet |
| **5** | --- | -3.64 | singlet |
| **TS11** | 24.9 | 39.3 | triplet |
| **7** | 2.5 | 23.03 | quartet |
| **TS12** | 9.76 | 34.27 | quartet |
| **8** | -38.83 | -17.6 | doublet |

## References


[1] S. Garhwal, A. Kaushansky, N. Fridman, L. J. W. Shimon, G. d. Ruiter, *J. Am. Chem. Soc.* **2020**, *142*, 17131-17139.

[2] I. S. Weitz, M. Rabinovitz, *J. Chem. Soc., Perkin Trans. 1* **1993**, 117-120.

[3] S. Garhwal, A. Kaushansky, N. Fridman, G. de Ruiter, *Chem. Catal.* **2021**, *1*, 631-647.

[4] C. N. Pierson, J. F. Hartwig, *Nat. Chem.* **2024**, *16*, 930-937.

[5] J. Klett, Ł. Woźniak, N. Cramer, *Angew. Chem. Int. Ed.* **2022**, *61*, e202202306.

[6] T. Kratz, P. Steinbach, S. Breitenlechner, G. Storch, C. Bannwarth, T. Bach, *J. Am. Chem. Soc.* **2022**, *144*, 10133-10138.

[7] M. D. R. Lutz, S. Roediger, M. A. Rivero-Crespo, B. Morandi, *J. Am. Chem. Soc.* **2023**, *145*, 26657-26666.

[8] G. Winter, D. W. Thompson, J. R. Loehe, in *Inorganic Syntheses*, **1973**, pp. 99-104.

[9] F. Neese, *WIREs Computational Molecular Science* **2012**, *2*, 73-78.

[10] F. Neese, *WIREs Computational Molecular Science* **2025**, *15*, e70019.

[11] Y. Zhao, D. G. Truhlar, *Theoretical Chemistry Accounts* **2008**, *120*, 215-241.

[12] R. Izsák, F. Neese, *The Journal of Chemical Physics* **2011**, *135*.

[13] S. Grimme, S. Ehrlich, L. Goerigk, *Journal of Computational Chemistry* **2011**, *32*, 1456-1465.

[14] S. Grimme, J. Antony, S. Ehrlich, H. Krieg, *J. Chem. Phys* **2010**, *132*, 154104.

[15] F. Weigend, R. Ahlrichs, *Physical Chemistry Chemical Physics* **2005**, *7*, 3297-3305.

[16] M. Steinmetz, S. Grimme, *ChemistryOpen* **2013**, *2*, 115-124.

[17] K. P. Jensen, B. O. Roos, U. Ryde, *The Journal of Chemical Physics* **2007**, *126*.

[18] A. V. Marenich, C. J. Cramer, D. G. Truhlar, *The Journal of Physical Chemistry B* **2009**, *113*, 6378-6396.

[19] A. D. Becke, *J. Chem. Phys.* **1993**, *98*, 5648-5652.

[20] C. Adamo, V. Barone, *J. Chem. Phys.* **1999**, *110*, 6158-6170.

[21] J.-D. Chai, M. Head-Gordon, *Phys. Chem. Chem. Phys.* **2008**, *10*, 6615-6620.

[22] Y. Zhao, D. G. Truhlar, *Theor. Chem. Acc.* **2008**, *120*, 215-241.

[23] A. D. Becke, *J. Chem. Phys.* **1996**, *104*, 1040-1046.

[24] M. Ernzerhof, G. E. Scuseria, *J. Chem. Phys.* **1999**, *110*, 5029-5036.

[25] R. H. Hertwig, W. Koch, *Chem. Phys. Lett.* **1997**, *268*, 345-351.

[26] J. P. Perdew, K. Burke, M. Ernzerhof, *Phys. Rev. Lett.* **1996**, *77*, 3865-3868.

[27] A. D. Becke, *Phys. Rev. A* **1988**, *38*, 3098-3100.

[28] V. Fock, *Zeitschrift für Physik* **1930**, *61*, 126-148.

[29] M. Besora, J.-L. Carreón-Macedo, A. J. Cowan, M. W. George, J. N. Harvey, P. Portius, K. L. Ronayne, X.-Z. Sun, M. Towrie, *Journal of the American Chemical Society* **2009**, *131*, 3583-3592.

[30] M. Reiher, O. Salomon, B. Artur Hess, *Theoretical Chemistry Accounts* **2001**, *107*, 48-55.

[31] M. Reiher, *Inorganic Chemistry* **2002**, *41*, 6928-6935.

[32] G. Chen, R. Liu, I. Silaghi-Dumitrescu, G. Espinosa-Perez, A. Zentella-Dehesa, F. Lara-Ochoa, *International Journal of Quantum Chemistry* **2001**, *83*, 60-69.

[33] J. N. Harvey, in *Principles and Applications of Density Functional Theory in Inorganic Chemistry I*, Springer Berlin Heidelberg, Berlin, Heidelberg, **2004**, pp. 151-184.

[34] Y. Zhao, D. G. Truhlar, *The Journal of Chemical Physics* **2006**, *125*.

[35] T. Husch, L. Freitag, M. Reiher, *Journal of Chemical Theory and Computation* **2018**, *14*, 2456-2468.
